# Ancestral Sequence Reconstruction to Accelerate Non-heme Iron-dependent Biocatalyst Engineering

**DOI:** 10.1021/acscentsci.5c01137

**Published:** 2025-09-26

**Authors:** José R. Hernández-Meléndez, Alexandra E. Paton, Jonathan C. Perkins, Di Yang, Chang-Hwa Chiang, Alison R. H. Narayan

**Affiliations:** †Department of Chemistry, ‡Life Science Institute, §Program in Chemical Biology, University of Michigan, Ann Arbor, Michigan 48109, United States

## Abstract

Nature provides access to biological catalysts that can expand the chemical transformations accessible to synthetic chemists. Among these, α-ketoglutarate, non-heme iron-dependent (NHI) enzymes stand out as scalable biocatalysts for catalyzing selective oxidation reactions. Many NHI enzymes require protein engineering to improve their activity, selectivity, or stability. However, the reliance of this strategy on the innate stability of the enzyme can thwart the success of the engineering campaign. Harnessing innately stable enzymes can overcome these challenges and accelerate biocatalyst engineering. Herein, we highlight the use of ancestral sequence reconstruction (ASR) to mine for thermostable enzymes that can serve as superior starting points for protein engineering. In our effort to develop a biocatalytic route to tropolones, we identified an NHI enzyme that demonstrated poor stability, diminished activity at high substrate concentrations, and a limited substrate scope. We compared the in-lab evolution of the modern NHI enzyme and its ancestor, demonstrating the improved evolvability profile of the latter. By engineering the ancestral protein, we accessed variants with enhanced thermostability and expression, increased rates, and a substrate scope broader than those of their modern counterparts. Altogether, this work provides a strategy to rapidly access enzyme backbones that can accelerate engineering of more robust and synthetically useful NHI enzymes.

## Graphical abstract

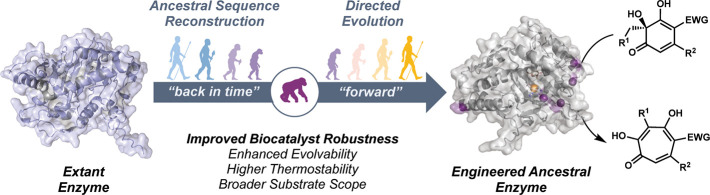

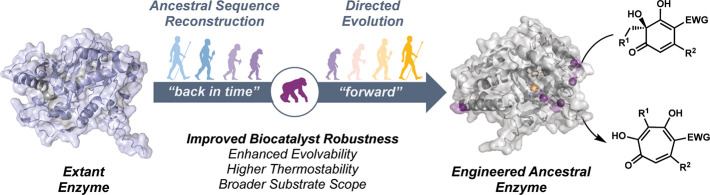

## Introduction

The chemistry achieved by Nature has historically impacted the field of organic chemistry, inspiring biomimetic routes to complex molecules and, more recently, providing access to a plethora of biological catalysts with a breadth of unique chemical reactivity.^1,2^ Enzymes have been evolved to perform valuable chemical reactions within the context of metabolism, utilizing submolar substrate concentrations to selectively produce a range of essential metabolites with impressive architectures and biological properties.^3–5^ Among the major classes of biocatalysts, the α-ketoglutarate, non-heme iron-dependent (NHI) enzyme family stands out as a dependable class for performing selective oxidation reactions on diverse substrates (Figure 1A).^6^ In a recent report, Renata and co-workers demonstrated the use of the natural NHI enzyme AneA, from the aculene A biosynthetic pathway in *Aspergillus aculeatus*, for desaturation and hydroxylation reactions, enabling the chemoenzymatic synthesis of feruginin.^7^ Importantly, industrial applications of NHI enzymes, such as engineered hydroxylases, demonstrate their utility on process scale.^8–11^ Moreover, the Abe^12^ and Ogasawara^13^ groups have recently discovered α-ketoglutarate-dependent NHI enzymes with unique reactivity such as cyclopropanation and aziridination which have been mechanistically investigated by Chang and co-workers.^14,15^ Seminal work from the Bollinger group demonstrated the utility of the α-ketoglutarate-dependent NHI halogenase SyrB2 toward nitration and azidation of aliphatic carbons.^16^ The latter reaction was also explored by the Lewis group in their efforts to engineer an α-ketoglutarate-dependent NHI hydroxylase to perform azidation chemistry.^17^ Beyond natural reactivity, the Fasan group discovered nitrene transfer activity with several characterized α-ketoglutarate-dependent NHI enzymes such as TauD, AsqJ, and WelO5 that led to the formation of sultam and oxazolidinone products.^18^ Arnold and co-workers also explored this new-to-nature chemistry by successfully evolving an α-ketoglutarate-dependent NHI enzyme for catalyzing new-to-nature nitrene transfer reactions to generate aziridines.^19^ The Huang group recently also expanded on the synthetic utility of this class of enzymes by engineering them to perform new chemistry such as abiotic Conia-ene reactions^20^ and C­(sp^2^)–S coupling^21^ upon substituting the Fe­(II) cofactor for Cu­(II) and Ni­(II) respectively.^22^ The exemplified prowess and versatility for scalable processes and diverse reactivity of this class of enzymes thus render them as valuable biocatalysts to develop further.

**Figure 1:**
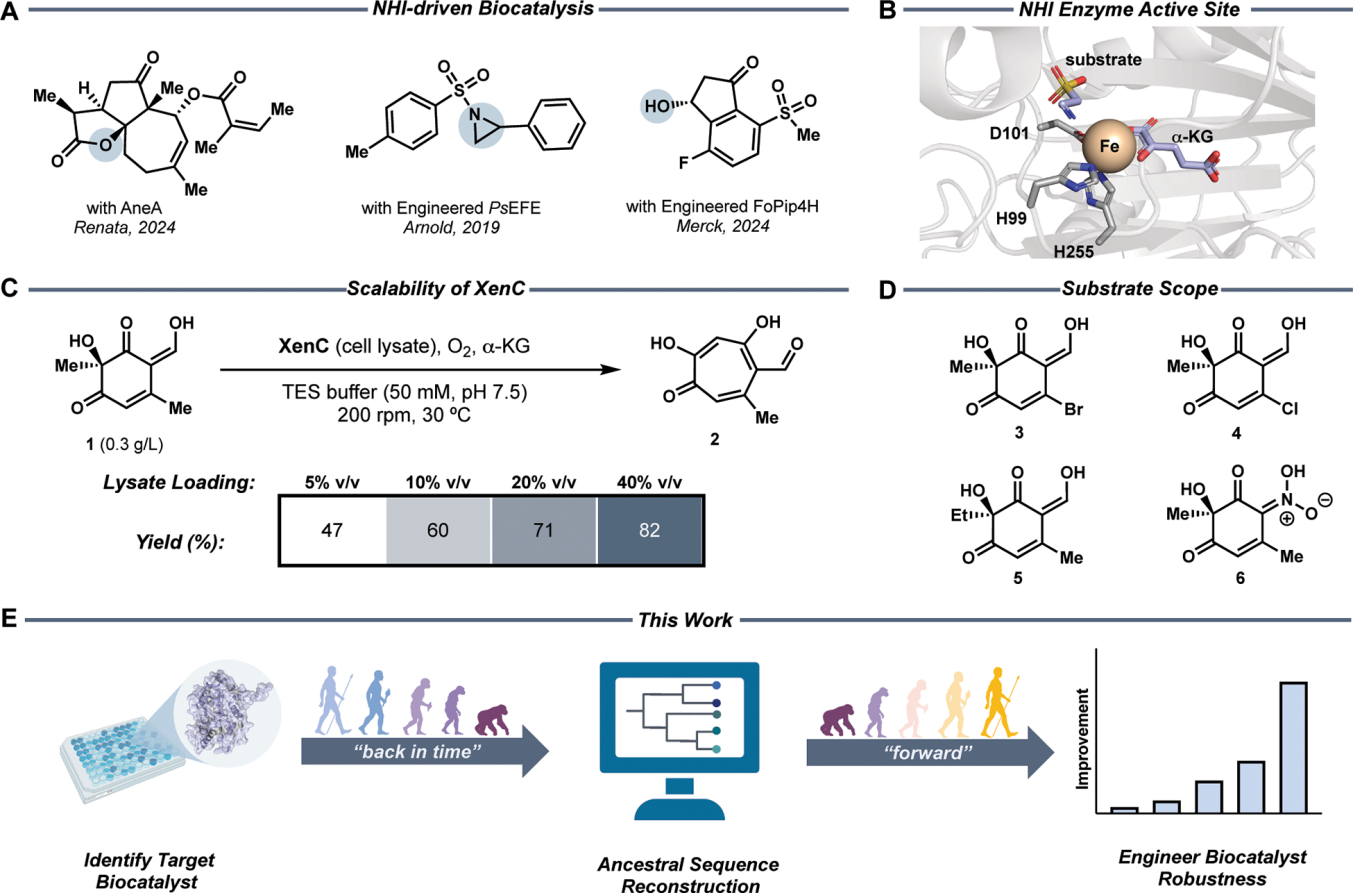
Engineering robustness of NHI biocatalysts. (A) Applications of NHI enzymes in biocatalytic strategies. (B) Active site of TauD (PDB 1OS7), a well-studied NHI enzyme. (C) Target tropolone-yielding enzymatic reaction selected for engineering enzyme robustness. Initial investigations of ideal lysate (40% v/v) and substrate concentrations (0.3 g/L). (D) Panel of substrates tolerated by XenC. (E) This work entails the identification of an enzyme that performs a desired reaction engineered by going “back in time” and intercepting traditional directed evolution strategies with ASR before continuing “forward” evolution toward superior biocatalyst robustness.

Although several NHI enzyme-driven biocatalytic strategies have been reported, many enzymes in this family can often be challenged by poor stability, scalability, and limited substrate scope. For example, Ma and co-workers engineered a (*trans*)-proline-4-hydroxylase from *uncultured bacterium* esnapd13 (*Ub*P4H) for the industrial bioproduction of (*trans*)-4-hydroxy-l-proline building blocks.^23^ Specifically, researchers wanted to use *Ub*P4H in fermentation cultures which required incubation at 37 °C. However, when surveying the thermostability of the enzyme, they found that upon incubation at 37 °C for 1 h, *Ub*P4H lost 48% of its activity. To improve upon its thermostability, Ma and co-workers used protein engineering to arrive at a *Ub*P4H variant which afforded 3.3-fold higher yield of (*trans*)-4-hydroxy-l-proline and a half-life 7.6-fold greater than the wild-type enzyme for biotransformations performed at 37 °C.^23^ Moreover, while investigating the NHI halogenase AdaV for nucleoside and nucleotide chlorination, Cheng and co-workers discovered that AdaV demonstrated limited tolerance of substrates with variation in the nucleobase, limiting the utility of this enzyme for the production of antiviral and anticancer nucleosides and nucleotides.^14^ By identifying alternate enzymes that possessed complementary substrate scope and using protein engineering to tune substrate selectivity, chemistry on nucleobases beyond adenine was achieved.^24^ Additionally, Merck’s effort to execute an improved route to belzutifan included an NHI hydroxylase which required 16 rounds of directed evolution to reach a robust enzyme capable of catalyzing kilogram-scale reactions. Eight of these rounds targeted thermostability improvements and the remainder focused on achieving a scalable and selective reaction.^8^ Identifying an NHI enzyme that possesses improved scalability, stability, and broad substrate tolerance is hence uncommon and can present a barrier to carrying out novel chemoenzymatic synthetic approaches that rely on this enzyme class.

These examples highlight the use of directed evolution to access synthetically useful NHI enzymes; however, successful examples of NHI enzyme engineering are limited compared to other classes of enzymes. In the case of NHI enzymes, one potential challenge is limited enzyme plasticity and stability, as these traits are proven to be important for the evolvabilty of proteins.^25,26^ This class of enzymes consists of intricate active site architectures (Figure 1B), characterized by a facial triad that reversibly binds iron and unique binding sites for the substrate and α-ketoglutarate (α-KG).^27^ For example, mutations on the secondary coordination sphere amino acids of factor inhibiting HIF (FIH), an NHI enzyme that serves as a sensor for hypoxia in human cells by hydroxylating N803 of the hypoxia inducible factor, unveiled the importance of hydrogen bond networks with secondary sphere residues to enable O_2_-activation and substrate positioning.^28^ Additionally, conformational changes are often necessary for productive substrate binding and catalysis, as demonstrated for TauD, among other NHI enzymes such as AusE, PrhA, and CurA.^29–31^ Therefore, small perturbations in the primary structure can have a substantial impact on binding and catalysis, which can lead to challenges in engineering this enzyme class.

To overcome these challenges, one can start with enzyme backbones that can better support amino acid substitutions without major disruptions in activity and stability. An effective strategy can be to engineer for thermal stability.^32^ Although this approach can deliver the desired properties, it may require extensive and labor-intensive engineering campaigns. Alternatively, selecting thermally stable enzyme backbones can bypass the need for engineering stability and allow for evolution focused on activity or selectivity. For example, mining for enzymes from thermophilic organisms can be a successful strategy, as highlighted by the efforts from Zheng and co-workers at engineering a glucose isomerase from the thermophilic bacterium *Thermoanaerobacter ethanalicus* toward higher yields of d-fructose.^33^ Additionally, lactate dehydrogenase from the thermophilic bacteria *Geobacillus stearothermophilus* was used by Clarke and co-workers as a starting point to remove substrate inhibition and change the substrate specificity of the enzyme from lactate to oxaloacetate through protein engineering.^34^ Alternatively, computational approaches provide a way to identify enzyme backbones that are more amenable to accessing robust biocatalysts. For example, Zalatan and co-workers harnessed ProteinMPNN^35^ to successfully produce *de novo* NHI enzymes with enhanced thermostability, enabling a more efficient protein engineering campaign of a proline-4-hydroxylase.^36^ Ancestral sequence reconstruction (ASR), an ancestral gene sequence prediction method that has historically yielded enzymes with enhanced thermostability, promiscuity (catalytic and substrate), and solubility, also provides a complementary computation-based approach.^37–50^

We recently encountered an α-ketoglutarate dependent NHI enzyme with limited robustness during the development of a biocatalytic approach toward tropolone products.^51^ Motivated by the biological properties^52–55^ of these seven-membered nonbenzenoid aromatic rings that are present in a plethora of natural products,^56,57^ Cox and co-workers first discovered the gene of the enzyme XenC which is an NHI enzyme involved in the biosynthesis of xenovulene A.^48^ Specifically, XenC produces stipitaldehyde (**2**) from dearomatized intermediate **1** through a skeletal rearrangement initiated by enzymatic C–H abstraction similar to the proposal with its homolog TropC (Figure 1C).^58,59^ The Ye group harnessed this transformation through the fermentation of *Nigrospora chinensis* GGY-3, allowing for the isolation of **2** to provide insight into its antimicrobial and antibacterial activity, albeit in low yields (0.005 g/L isolated).^60^ The potential to realize rapid access to diverse tropolone-containing products through a two-step enzymatic sequence from resorcinol substrates inspired us to pursue a scalable biocatalytic approach toward densely functionalized tropolones that could be carried out *in vitro* to access meaningful quantities of natural and unnatural tropolones (Figure 1C–D).^41^ However, we quickly identified limitations in using XenC as a catalyst. For example, the use of XenC in vitro was hindered by its low T_50_ (35 °C), requirement for 40% v/v cell lysate loading to turnover 0.3 g/L of substrate (Figure 1C), and limited substrate tolerance (**3**–**6**, Figure 1D). To overcome these shortcomings, we initially surveyed a library of XenC homologs, however, no enzymes were identified with improved biocatalytic performance compared to XenC.^41^ Therefore, we anticipated that protein engineering could improve these qualities. Herein, we detail efforts to engineer XenC and its predicted ancestor, Anc1, toward gaining qualities of enzyme robustness. By comparing these two approaches, the utility of ASR as a strategy to access evolvable NHI enzyme backbones and more rapidly engineer synthetically robust tropolone-forming NHI enzymes is showcased (Figure 1E).

## Results/Discussion

### Directed Evolution of Extant NHI Enzyme XenC

To begin improving the scalability profile of XenC, we aimed to engineer better tolerance of higher loadings of **1** using the 1.2 g/L substrate as the pressuring condition (Figure 2A). Using the AlphaFold2 ^61,62^ predicted model of XenC combined with the crystal structure of its homolog TropC (PDB: 6XJJ),^63^ we designed a site saturation mutagenesis (SSM) library targeting 96 residues within 17 Å of the active site and <95% residue conservation (Figure S6A, Table S2). The selected sites for saturation included residues in the active site, second coordination sphere, and surface of the enzyme to have a broad representation of all regions in proximity to the active site. With the libraries generated, we screened the variants of the first round of evolution using RapidFire Mass Spectrometry (RF-MS) as the detection platform.

**Figure 2:**
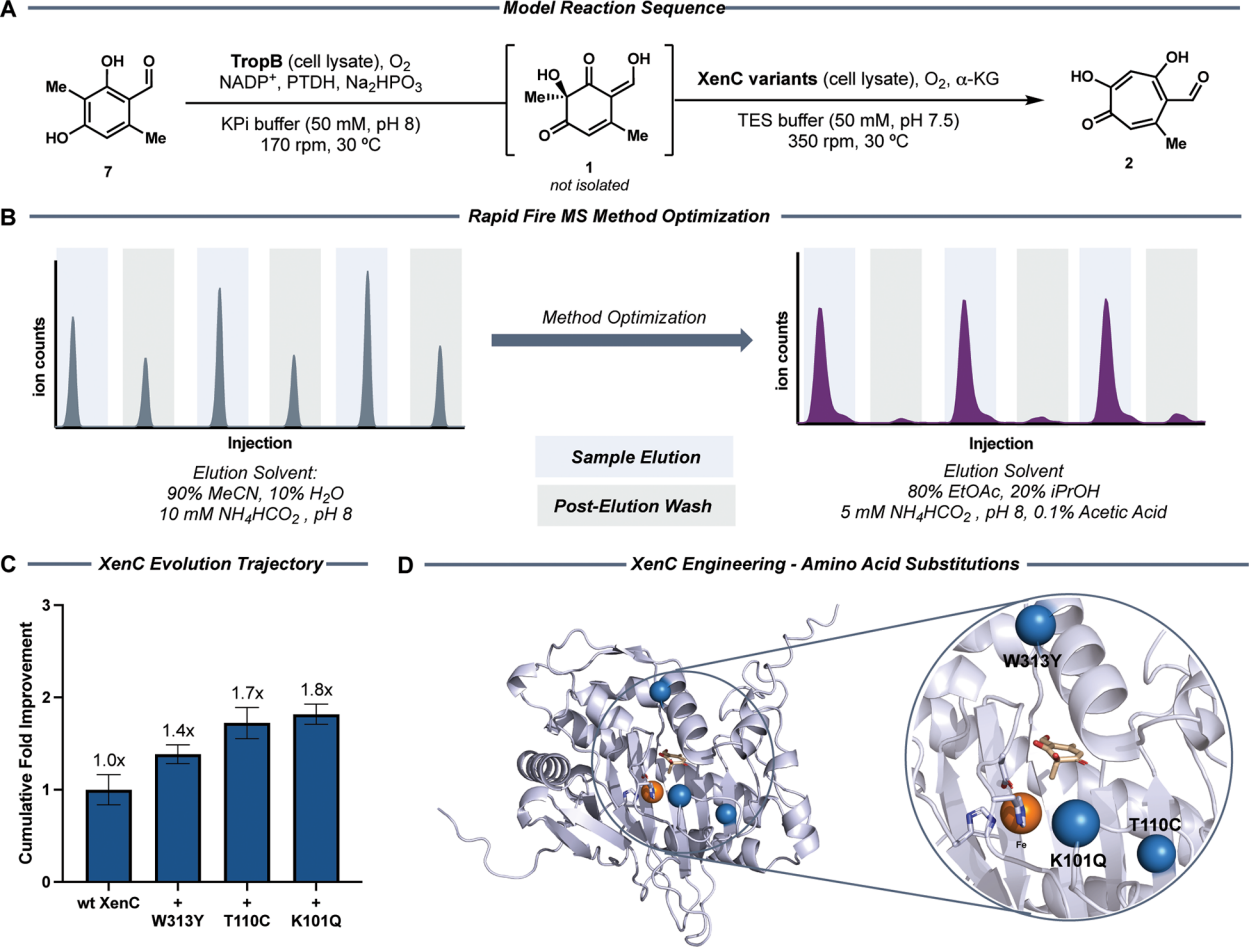
Directed evolution of XenC. (A) Model reaction sequence for screening XenC variant libraries. (B) Optimization of high-throughput screening method to detect tropolone products. (C) The evolution trajectory of XenC shows negligible improvements in tropolone formation over three rounds of protein engineering. Clarified cell lysate used in enzymatic reactions for variant comparison was incubated for 1 h at 37 °C with shaking at 350 rpm. Biocatalytic reactions were performed with 5% v/v clarified lysate and 1.2 g/L substrate **1** at 30 °C and 350 rpm shaking for 1 h. D. AlphaFold predicted model of XenC with substrate (beige) and iron (orange) docked, demonstrating the location of the incorporated amino acid substitutions (blue).

During the primary analysis of our SSM library, we noted that there was significant tropolone carryover between sample injections on the RF-MS instrument, which led to inaccurate product quantification. Although we have previously used RF-MS methods to successfully screen a variety of compounds, the standardized methods previously developed did not address tropolone carryover. A similar effect was observed when using highly sensitive chromatographic methods such as LC-MS (Figure S10). We suspected that this observation is due to the chelating abilities of tropolones,^55,64,65^ which could be leading to strong interactions with the stationary phases of our analysis platforms. To overcome this challenge, we optimized a method for our RF-MS platform that decreased carryover using stipitaldehyde (**2**) as a model analyte. A screen of elution solvents led us to identify a buffered ethyl acetate/isopropanol-based solvent system that takes advantage of the previously investigated solubility of stipitaldehyde^51^ and led to decreased tropolone carryover (Figure 2B). This optimized method resulted in reliable quantification of **2** and provided an improved platform to screen our tropolone-yielding enzyme libraries with greater accuracy (Figure S9).

Using the optimized RF-MS method, we successfully screened the initial SSM library and identified XenC_W313Y (XenCRd1), a surface residue, as the best performing variant with a 1.4-fold increase in tropolone formation activity over that of wild-type XenC (wt-XenC, Figure 2C). Although a slight improvement in conversion was obtained, only 28% of variants showed detectable tropolone-forming activity. To bypass this setback, we turned to the screening data from our SSM library to inform an alternate mutagenesis route. We identified three sites (S128, V247, and T110) that repeatedly showed a range of mutations with comparable activity to the template of each library. Due to the recurrence of substitutions at these sites, we targeted S128, V247, and T110 for saturation and recombination simultaneously (combi-SSM) on the *xenCRd1* gene to explore combinations that could provide more significant improvement. To our surprise, we identified the single-substitution variant XenC_W313Y/T110C (XenCRd2), which incorporated a substitution on the active site residue T110, as the top hit from the combi-SSM library (Figure 2C), and none of the variants carried on for hit validation showed more than one mutation, although full incorporation and recombination of all sites was observed during our library quality assessment. These findings led us to believe that we could be facing a challenge of enzyme stability, preventing the identification of variants with improved performance due to the loss in overall activity as a result of the incorporation of mutations.^25,66^

To confirm this hypothesis, we investigated the temperature at which 50% of enzyme activity is lost (T_50_) for wt-XenC, XenCRd1, and XenCRd2. Indeed, wt-XenC lost half of its activity at 34–38 °C, and variants XenCRd1 and XenCRd2 also showed comparable T_50_ values of 37.5 and 38.5 °C, respectively (Table S12), supporting the hypothesis that the stability of the enzymes could be hindering their evolvability. Previous reports have identified that surface residues can often be targeted in protein engineering campaigns to improve enzyme stability.^67–69^ For this reason, we built a new library by selecting 96 surface residues using the surface residue function on PyMol (Figure S6B, Table S4). With this new SSM library, we aimed to improve the thermostability of XenCRd2 alongside improving the conversion at a high substrate concentration. To make sure we selected thermally stable enzymes during our screening, we applied a temperature pressure (40 °C preincubation, 1 h) on the crude lysate before screening reactions under high substrate conditions. Although 29% of enzyme variants showed detectable activity after temperature treatment, no significant improvement in conversion was observed. The best variant identified, XenC_W313Y/T110C/K101Q (XenCRd3), showed 1.8-fold improvement in tropolone formation activity over wt-XenC and incorporated a glutamine substitution in place of the surface lysine, K101 (Figure 2C). We investigated the thermal stability of this final variant and found a comparable T_50_ to XenCRd2 (Table S12), suggesting that no improvements in stability were achieved although temperature was used as an evolution pressure. These results suggest that evolving XenC is challenging, with only minor improvements in stability and expression being conducive to the overall enzyme robustness goal, and thus, several more rounds of evolution would likely be required to access a catalyst with the desired traits.

### Directed Evolution of Ancestral NHI Enzymes

The attempted evolution of wt-XenC highlights the important relationship between stability and evolvability and its effect on the success of an evolution campaign. We anticipated, however, that the innate stability of proteins discovered through ASR could provide a more evolvable starting point to access more robust tropolone-forming enzymes. To investigate this, we surveyed a panel of XenC ancestral proteins generated in our laboratory (Figure 3A) to find an enzyme with greater stability than wt-XenC that could potentially allow for better incorporation of beneficial mutations. Previously, we achieved a change in the dominant reaction pathway for an NHI benzylic hydroxylase (BHO), to enable the XenC-like ring-expansion.^70^ We used ASR to resurrect the sequences of ancestral enzymes related to XenC and BHO to determine which amino acid residues could control the dominant reaction pathway. Upon surveying the thermostability and reactivity of these ancestral NHI enzymes, we identified that Anc1 retained tropolone forming activity comparable to XenC and showed a T_50_ of 53.5 °C (Figure 3B). Whereas these protein sequences, XenC and Anc1, share 68.9% sequence identity (Figure S4), their stability profiles are significantly different (∼18 °C difference). Therefore, based on the enhanced thermostability and comparable activity of Anc1 in relation to XenC, we selected the former as a backbone to evolve overall robustness and compare to the evolution trajectory of wt-XenC.

**Figure 3:**
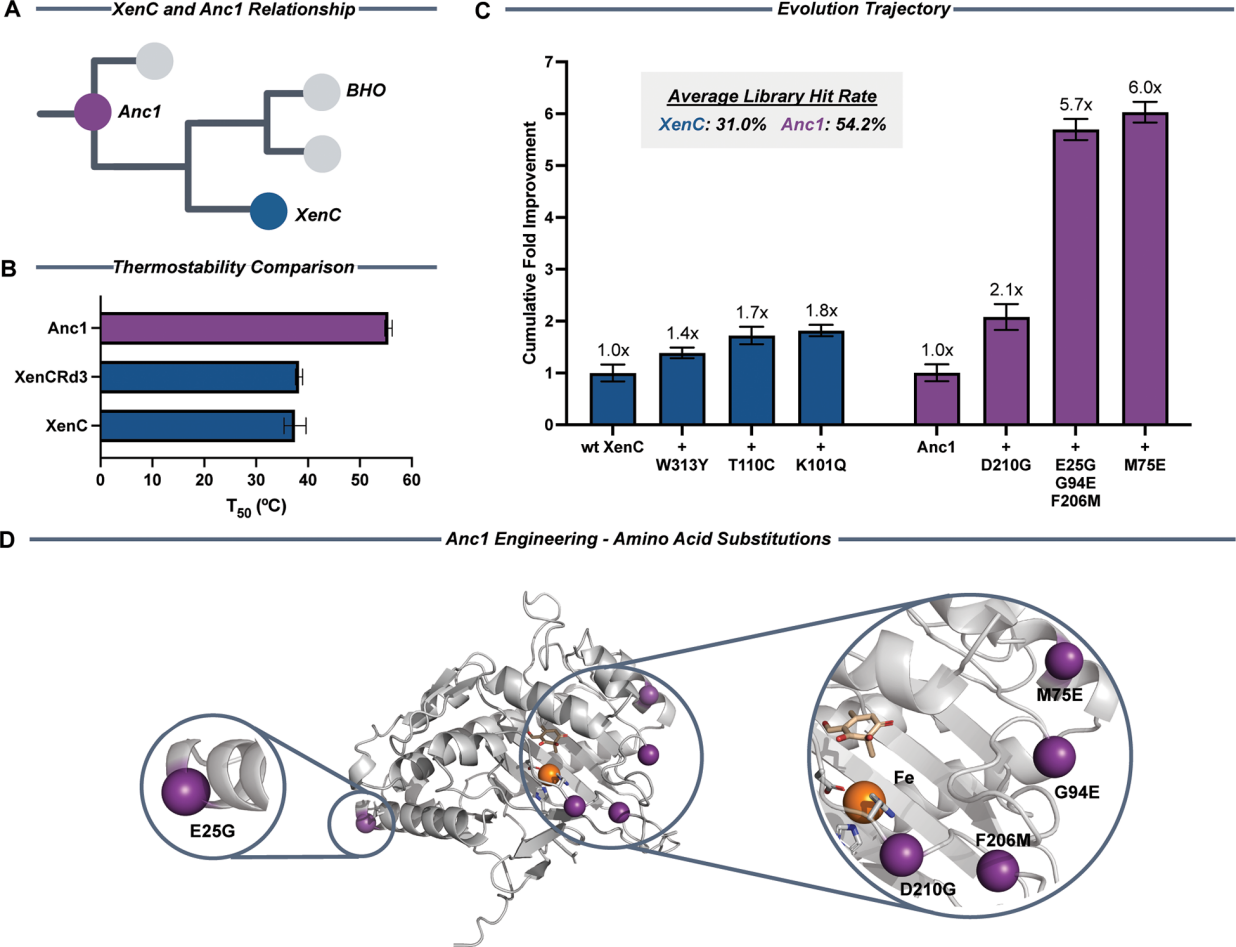
Directed evolution of Anc1. (A) Phylogenetic relationship of XenC and Anc1 based on the ancestral sequence reconstruction of NHI benzylic hydroxylase (BHO). (B) Comparison of the T_50_ values of wt-XenC, XenCRd3 and Anc1 demonstrates more than 10 °C in thermostability gained from ASR. (C) Evolution trajectory of Anc1 compared to the activity of wt-XenC and XenCRd3. Clarified cell lysate used in enzymatic reactions for variant comparison was incubated for 1 h at 37 °C with shaking at 350 rpm. Biocatalytic reactions were performed with 5% v/v clarified lysate and 1.2 g/L substrate **1** at 30 °C and 350 rpm shaking for 1 h. (D) AlphaFold predicted model of Anc1 with docked substrate (beige) and iron (orange) and all amino acid substitutions identified (purple).

From the amino acid sequence of Anc1, we first modeled its tertiary structure by using AlphaFold2. The proposed structure shows the characteristic “jellyroll” architecture and the catalytic triad of NHI enzymes, matching with the modeled structure of wt-XenC and the crystal structure of TropC (Figure S5). With this structural model of Anc1, we designed a SSM library that targeted 96 residues across the active site, second coordination sphere, and surface of the enzyme with <95% conservation, similarly to what was performed with wt-XenC (Figure S6C, Table S5). As Anc1 already possessed ideal thermostability, we focused on evolving for scalability using substrate **1**, screening for variants that showed improvement in stipitaldehyde (**2**) formation at low catalyst loadings (5–10% v/v lysate compared to initial conditions at 40% v/v) and high substrate concentration (1.2 g/L). To maintain the innate stability of the ancestor, we also preincubated all reactions for 1 h at temperatures >40 °C to select for variants with retained or enhanced thermal stability.

For the first round of evolution, we aimed to increase conversion after a 50 °C heat treatment for an hour followed by reactions with 1.2 g/L substrate concentration and 10% v/v lysate loading. Upon profiling variants, we identified twice as many active enzymes as compared to our XenC evolution, suggesting greater overall enzyme backbone tunability (Table S7). Anc1_D210G (Anc1Rd1) incorporated a substitution on the active site residue D210 and was selected as the top hit to move forward, which showed higher improvement (2.2-fold) in conversion with a single amino acid substitution than what was obtained with the final triple mutant of XenC (Figure 3C). Because we also found a surfeit of variants that showed >1.5-fold enhancement in stipitaldehyde formation, we hypothesized that designing a Combi-SDM library could yield a variant with accumulative improvements as we had originally proposed with XenC. Using the *anc1Rd1* gene as a template, we prepared a combinatorial library using primers that encoded for E25G, F206M, F206S, F206K, G94E, Q28G, E230T, and R204E with the goal of probing the library for improvements in conversion as a result of recombination of mutations. In tandem, we surveyed another SSM library that targeted the same sites as our first round of ancestor-based directed evolution.

For this second round of evolution, we further challenged the enzyme by lowering the lysate loading to 5% v/v while continuing to load 1.2 g/L of substrate and preheat at 50 °C for 1 h. After screening and validation, we identified Anc1_D210G/E25G/G94E/F206M (Anc1Rd2) from the combinatorial library as our top hit, showing a 5.7-fold improvement in conversion over Anc1 (Figure 3C). The series of substitutions incorporated included surface (E25 and G94) and second-sphere (F206) residues based on our AlphaFold model. A final round of SSM targeting the same sites as previous rounds led us to the identification of Anc1_D210G/E25G/G94E/F206M/M75E (Anc1Rd3), which incorporated the surface residue substitution M75E and showed 6-fold improvement in tropolone formation over Anc1 and wt-XenC (Figure 3C). During this final round, we also surveyed another combinatorial library that included N21R, Q28G, G94F, P138A, R166G, R204E, and E230T substitutions but did not identify a better variant than Anc1Rd3. Because we screened under low lysate loadings, we suspected that part of the improvements observed were related to increased amounts of soluble protein in the lysate. To support this, we estimated the relative amount of soluble enzyme in lysate by SDS-PAGE gel densitometry and found 2.2-fold more enzyme in Anc1Rd3 lysate than wt-XenC and Anc1 (Table S1). It is interesting to note that the substitutions incorporated in Anc1Rd3 cluster close to the active site of the enzyme except for E25G (Figure 3D). However, reversion of this E25G mutation led to a 50% loss in product formation (Figure S14).

Next, we turned our attention to better understand the individual effects that lead to the observed improvements in conversion with Anc1Rd3 during our evolution as compared to XenC. Since the Anc1Rd3 lineage provided more stable enzymes, we initially considered an improvement in catalyst stability over the course of the reaction. Time course reactions were performed with XenC and Anc1Rd3 in purified form to determine the intrinsic stability of the biocatalyst over the course of the reaction. From this data, we did not find any differences in the longevity of both catalysts, with both enzymes reaching a plateau within 1–2 h (Figure S15). As no improvements in longevity were observed, we then considered whether an enhancement in the initial rate was contributing to the observed improvements in conversion (Figure 4A). Reactions with pure enzyme were run to determine the initial rates of product formation. For XenC, an initial velocity of 0.20 s^–1^ was measured, whereas, Anc1Rd3 showed an initial velocity of 0.72 s^–1^, demonstrating that Anc1Rd3 has 3.6-fold higher initial rate than XenC. In combination with the enhanced thermostability and expression, these findings could account for the overall improvements in conversion observed through the evolution. Additionally, we determined the initial rate of Anc1 and found that this enzyme displays a lower initial rate than its evolved variant Anc1Rd3 (0.45 s^–1^ versus 0.72 s^–1^, respectively), providing evidence that this catalytic property can be improved through protein engineering. Finally, to contextualize the improvements in thermostability and expression in the reaction yield, we preincubated cell lysates bearing XenC and Anc1Rd3 at room temperature and 50 °C prior to performing reactions with 2% v/v treated lysate and 1.2 g/L substrate **1** and compared the yield of product (Figure 4B). As expected, we observed higher concentration of stipitaldehyde product in reactions containing Anc1Rd3 as compared to XenC. We also found that this activity can be retained after treating Anc1Rd3 with high temperature (50 °C), whereas, XenC did not show any detectable product, showcasing the broad tolerance to temperature of the ancestral variant.

**Figure 4:**
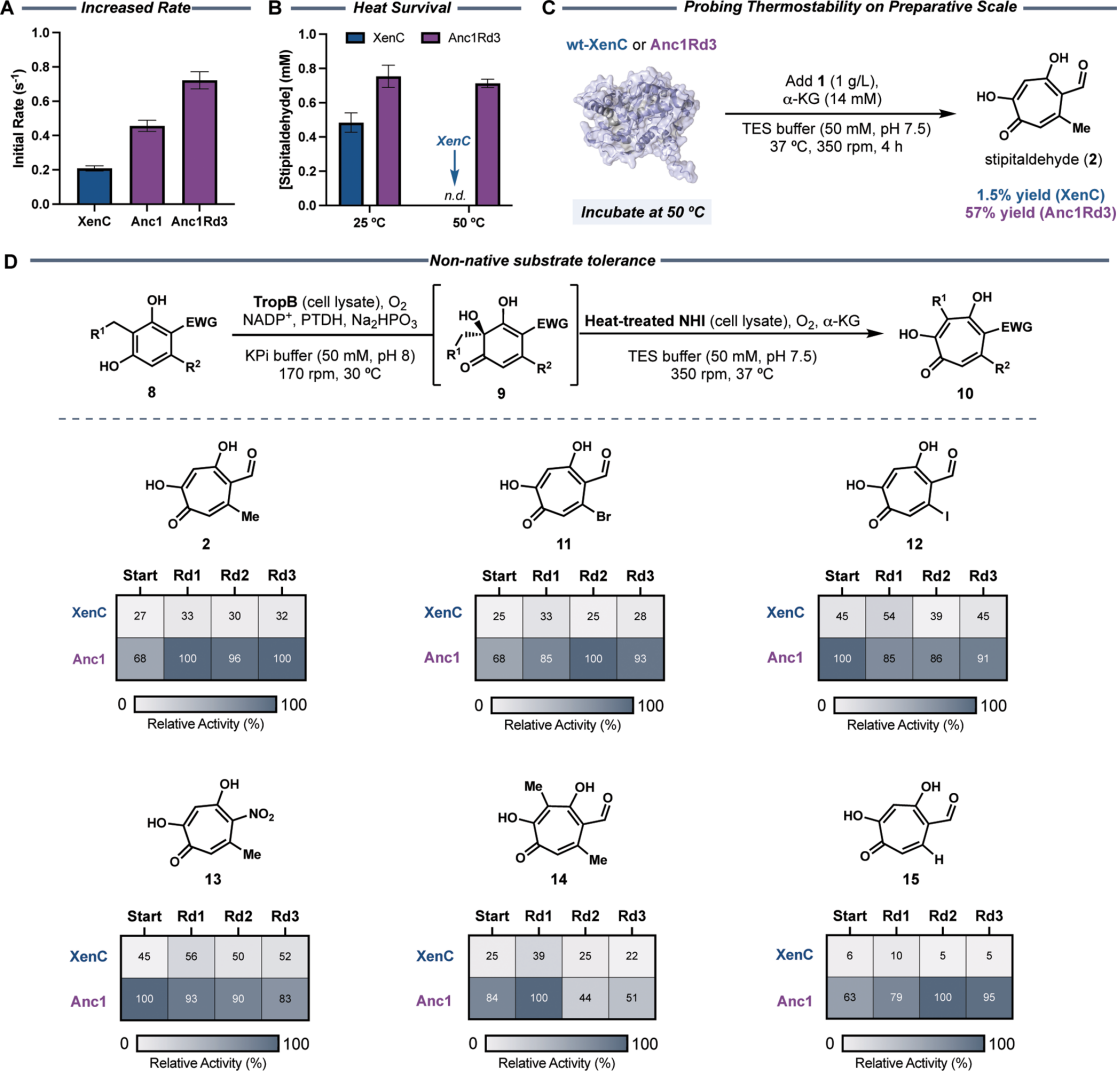
Surveying the improvements and synthetic utility of engineered ancestral enzymes. (A) Anc1 demonstrates enhanced initial velocity compared to XenC, which was further improved upon with Anc1Rd3. (B) Reactions with lysate bearing Anc1Rd3 show retention of enzymatic activity upon drastic heat challenges compared to XenC. Reactions were performed at 30 °C with 2% v/v cell lysate preincubated at 25 or 50 °C, and 1.2 g/L substrate **1** for 1 h. (C) Probing the thermostability of XenC and Anc1Rd3 on preparative scale demonstrates that Anc1Rd3 portrays more robust qualities. Clarified cell lysates harboring wt-XenC and Anc1Rd3 were incubated at 50 °C for 15 min prior to adding reaction components. Biocatalytic reactions were performed at 37 °C with 30% v/v cell lysate and 1 g/L substrate **1**. (D) Substrate scope of XenC, Anc1, and variants. For all reactions, empty vector (pET28a) lysate was used as the negative control. Reactions were performed by adding 0.3–0.6 g/L substrate and 50% v/v heat-treated clarified cell lysate. All reactions were performed in three biological replicates. The relative activity is reported in relation to the best performing enzyme for each substrate.

### Investigating the Synthetic Utility of Anc1 and Variants

From our profiling and engineering efforts, we have gained access to a robust tropolone-forming biocatalytic method with enhanced stability, increased rate, and evolvability. To probe whether the robustness of the evolved ancestral enzyme translates to larger reaction scales, we investigated the behavior of Anc1Rd3 and wt-XenC under preparative-scale conditions that simultaneously challenged their stability and substrate loading tolerance (Figure 4C). To test the survival of the enzymes under high temperatures, we preincubated cell lysates containing Anc1Rd3 and XenC at 50 °C for 15 min. After the temperature treatment, we observed drastic precipitation of wt-XenC, whereas Anc1Rd3 remained in solution. Analysis of the precipitate by SDS-PAGE showed a major band with the corresponding size of XenC (Figure S26). With this heat-treated lysate sample, reactions with 30% v/v lysate, 1 g/L substrate (>3 times more substrate than the original conditions), and a higher reaction temperature (37 °C) were run for 4 h. Stipitaldehyde was successfully isolated in 57% yield when using Anc1Rd3 as the catalyst, 38-fold more product than what was obtained with wt-XenC (Figure 4C). These findings render cell lysates harboring Anc1Rd3 more synthetically robust biocatalytic systems that are able to perform under conditions that traditionally hinder the preparative-scale application of enzymatic reactions.

Beyond the scalability of tropolone-forming NHI enzymes, we investigated the utility of XenC and Anc1 variants with a panel of substrates, as ASR can provide enzymes with enhanced substrate promiscuity.^71^ Therefore, we expected that Anc1 and the generated variants could possess an expanded substrate scope compared to wt-XenC. Heat treated cell lysates with Anc1 and variants showed higher reactivity with all of the substrates previously oxidized by wt-XenC, showing good retention of substrate tolerance (Figure 4D). To further explore the substrate scope, we also investigated the ability of Anc1 to produce tropolone **15**, a compound not accessible with wt-XenC (Figure 4D). Gratifyingly, Anc1 and variants yielded tropolone product **15**, demonstrating the enhanced promiscuity of ancestral proteins and expanding on the chemical space accessible by these tropolone-forming NHI enzymes. These results, combined with the improved scalability, stability, and tunability of the ancestral enzymes evolved herein, provide a potential platform for further evolution to enhance this newly accessed reactivity and expand the substrate scope of this reaction.

## Conclusion

Overall, our efforts toward the evolution of XenC and Anc1 reveal that the latter enzyme is a more fruitful starting point to evolve enzyme robustness and expand the substrate scope of a ring-expansion reaction. The enhanced stability of Anc1 provided a backbone that showed superb evolvability, incorporating a total of five mutations through single-site and combinatorial mutagenesis without trade-offs in stability. The libraries generated from Anc1 also provided higher average hit rates compared to those of XenC libraries, providing further support for the improved evolvability of the former. The additive effects of the amino acid substitutions allowed the optimization of a biocatalytic method that can tolerate higher loadings of substrate at higher temperatures. Moreover, accessing Anc1 and its evolved variants allowed for twice as much soluble enzyme and opened the door to explore reactivity with diverse substrates. Further engineering of these newly accessed enzyme backbones could enhance activity with these substrates and yield a wider array of synthetically useful tropolone products. More broadly, however, we believe that applying this ASR-based “*back in time*” protein engineering strategy followed by a “*forward*” evolution on other enzyme classes could prime biocatalysts for more labor-efficient in-lab evolution campaigns to provide enzymes with enhanced synthetic utility.

## Supplementary Material



## Notes

DNA and protein sequences, primers, chromatograms, additional experimental details, materials, methods, and NMR spectra of compounds (PDF)
