# Supplementary material for: Ancestral Sequence Reconstruction to Accelerate Non-heme Iron-dependent Biocatalyst Engineering
Source: ACS Cent Sci. 2025 Sep 26;11(11):2196–205. doi: 10.1021/acscentsci.5c01137 (PMC12670320; doi:10.1021/acscentsci.5c01137)
Supplement: Supplementary file 1 [file oc5c01137_si_001.pdf]

Supporting Information for

Ancestral Sequence Reconstruction to Accelerate Non-heme Iron-dependent Biocatalyst Engineering

José R. Hernández-Meléndez,[1,2] Alexandra E. Paton,[2] Jonathan C. Perkins,[1,2] Di Yang,[1,2] Chang-Hwa Chiang,[1,2] Alison R. H. Narayan[1,2,3]

Corresponding Author: arhardin@umich.edu

[1]Department of Chemistry,[2]Life Science Institute,[3]Program in Chemical Biology, University of Michigan, Ann Arbor, MI 48109, United States

Table of Contents

I. Chemical Synthesis **3**
General Information. 3
Substrate Synthesis. 4
II. Protein Sequences and Expression **6**
Plasmids and Amino Acid Sequences 6
Preparative-scale flavin-dependent monooxygenase (FDMO) protein production 10
Preparative-scale α-ketoglutarate non-heme dependent iron (NHI) enzyme protein production 10
Preparative-scale preparation of clarified cell lysate 10
Purification of α-ketoglutarate non-heme dependent iron (NHI) enzymes. 11
Quantification of soluble protein 12
III. Directed Evolution of NHI enzymes **14**
Generation of AlphaFold Structures. 14
Selection of target residues. 15
Site-saturation mutagenesis. 16
Multiple site-directed mutagenesis. 16
Site-directed mutagenesis 16
Protein library production in 96-well culture plates 24
Protein production for validation experiments in 96-well culture plates 24
IV. Biocatalytic Reactions **25**
Stock solutions.......... 25
Oxidative dearomatization with TropB for library experiments 25
NHI lysate reactions for library screening and validations 25
Calculating Relative Percent Conversion 26
High throughput RapidFire-MS method 26
In vitro analytical-scale NHI lysate reactions with variants 30
Time course reaction. 33
Initial Rate Determination. 34
Analytical-scale Heat Challenge Experiments. 35
Analytical-scale oxidative dearomatization of substrate panel with TropB 36
Analytical-scale NHI lysate reactions with substrate panel 36
Preparative-scale ring expansion reactions. 43
V. NMR Spectra **44**
VI. References **51**

I. Chemical Synthesis

General Information. All reagents were used as received unless otherwise noted. Reactions were carried out under a nitrogen atmosphere using standard Schlenk techniques unless otherwise noted. Solvents were degassed and dried over aluminum columns on an MBraun solvent system (Innovative Technology, inc., Model PS-00-3). Reactions were monitored by thin layer chromatography using Millipore 60 F254 pre-coated silica thin layer chromatography (TLC) plates (0.25 mm) which were visualized using ultraviolet light (UV), potassium permanganate ($KMnO_4$), cerium ammonium molybdate (CAM), or dinitrophenylhydrazine (DNP) stains or by high performance liquid chromatography (HPLC) analysis using an Agilent 1290 Series Infinity II HPLC equipped with a diode array detector. Flash column chromatography was performed using Machery-Nagel 60 µm (230-400 mesh) silica gel. All compounds purified by column chromatography were sufficiently pure for use in further experiments unless otherwise indicated. ^{1}H and ^{13}C NMR spectra were obtained in $CDCl_3$ or CD_3OD at rt (25 ºC), unless otherwise noted, on a Varian 600 MHz spectrometer equipped with a liquid nitrogen chilled Bruker Prodigy (^{1}H/^{19}F)-X broadband probe. Chemical shifts of ^{1}H NMR spectra were recorded in parts per million (ppm) on the δ scale. High resolution electrospray ionization (ESI) mass spectra were obtained on an Agilent 1290 Series Infinity II HPLC with a 6230 Series Time-of-Flight (TOF) mass spectrometer (MS) or an Agilent RapidFire 400 (RF) with a 6230 Series TOF-MS. UPLC-DAD chromatogram traces were obtained on an Agilent 1290 Series Infinity II HPLC instrument equipped with a diode array detector.

Substrate Synthesis. Substrates used in reactions with TropB were synthesized according to previously developed procedures unless otherwise noted. Substrate **S10** was purchased from Sigma Aldrich and was used without further purification.

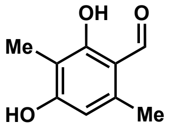

7

2,4-dihydroxy-3,6-dimethylbenzaldehyde (7) was prepared following protocols previously reported by our group.[1] All spectra obtained were consistent with literature values.**^{1}H NMR** (599 MHz, CD_3OD) δ (ppm) 10.04 (s, 1H), 6.22 (s, 1H), 2.47 (s, 3H), 1.98 (s, 3H).

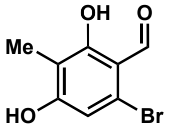

S1

6-bromo-2,4-dihydroxy-3-methylbenzaldehyde (S1) was prepared following protocols previously reported by our group.[2] All spectra obtained were consistent with literature values.**^{1}H NMR** (599 MHz, $CDCl_3$) δ (ppm) 12.68 (s, 1H), 10.11 (s, 1H), 6.69 (s, 1H), 5.43 (s, 1H), 2.08 (s, 3H).

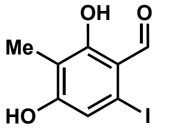

S2

2,4-dihydroxy-6-iodo-3-methylbenzaldehyde (S2) was prepared following protocols previously reported by our group.[2] All spectra obtained were consistent with literature values.**^{1}H NMR** (599 MHz, CD_3OD) δ (ppm) 9.76 (s, 1H), 6.97 (s, 1H), 1.97 (s, 3H).

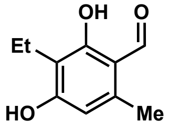

S3

3-ethyl-2,4-dihydroxy-6-methylbenzaldehyde (S3) was prepared following protocols previously reported by our group.[2] All spectra obtained were consistent with literature values.**^{1}H NMR** (599 MHz, $CDCl_3$) δ (ppm) 12.55 (s, 1H), 10.01 (s, 1H), 6.12 (s, 1H), 5.48 (s, 1 H), 2.54 (q, J = 7.5 Hz, 2H), 2.42 (s, 3H), 1.05 (t, J = 7.5 Hz, 3H).

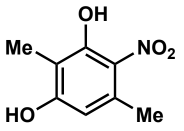

S4

2,5-dimethyl-4-nitrobenzene-1,3-diol (S4) was prepared following protocols previously reported by our group.[3] All spectra obtained were consistent with literature values.**^{1}H NMR** (599 MHz, CD_3OD) δ (ppm) 6.31 (s, 1H), 2.48 (s, 3H), 2.06 (s, 3H).

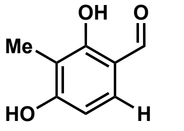

S5

2,4-dihydroxy-3-methylbenzaldehyde (S5) was prepared following protocols previously reported by our group.[2] All spectra obtained were consistent with literature values.**^{1}H NMR** (599 MHz, CD_3OD) δ (ppm) 9.63 (s, 1H), 7.31 (d, J = 8.51 Hz, 1H), 6.47 (d, J = 8.54 Hz, 1H), 2.04 (s, 3H).

II. Protein Sequences and Expression

Plasmids and Amino Acid Sequences

The gene encoding *tropB* (Accession ID - B8M9J8) was codon optimized for overexpression in *E. coli* and synthesized by GeneArt (Thermofisher). The synthesized sequence was cloned by GeneArt into a pET151 vector containing the T7 expression system, ampicillin resistance, and N-terminal 6xHis-tag encoded upstream from the inserted gene as previously reported.[2] The genes *xenC* (Accession ID - A0A2U8U2L3.1) and *anc1* were codon-optimized for overexpression in *E. coli* and synthesized by Twist Biosciences. The synthesized sequences were each cloned by Twist biosciences into a pET28a vector containing the T7 expression system, kanamycin resistance, and N-terminal 6xHis-tag encoded upstream from the insert gene as previously reported.[1,4]

TropB sequences:

```
ATGCCTGGTAGCCTGATTGATACCCGTCAGCAGCCGCTGAGCGTTGGTATTGTTGGTGGTGGTATTATTGGCGTTATTCTG
GCAGCAGGTCTGGTTCGTCGTGGTATTGATGTTAAAGTTTTTGAACAGGCACGTGGCTTTCGTGAAATTGGTGCAGGTATG
GCATTTACCGCAAATGCAGTTCGTTGTATGGAAATGCTGGATCCGGCAATTGTTTGGGCACTGCGTAGCAGCGGTGCAGTT
CCGATTAGCATTGGTGATCATCAGGCCGAAGCACGTGATTATCTGCGTTGGGTTGATGGTTATCATGAAAGCAGCAAACGT
CTGTATCAGCTGGATGCAGGTATTCGTGGTTTTGAAGCATGTCGTCGTGATCAGTTTCTGGAAGCACTGGTTAAAGTTCTG
CCGGAAGGTATTGTGGAATGTCAGAAACGTCTGCAGAAAATCCACGAAAAAAACGAAACCGAAAAAGTGACCCTGGAATTT
GCAGATGGCACCTTTGCACATGTTGATTGTGTTATTGGTGCCGATGGTATTCGTAGCCGTGTTCGTCAGCACCTGTTTGGT
GAAGATAGCCCGTATAGCCATCCGCATTATAGCCATAAATTTGCATTTCGTGGTCTGATCACCATGGAAAATGCAATTAGC
GCACTGGGCGAAGATAAAGCACGTACCCTGAATATGCATGTTGGTCCGAATGCACATCTGATTCATTATCCGGTTGCAAAT
GAAACCATGGTGAATATTGCAGCCTTTGTTAGCGATCCGGAAGAATGGCCTGATAAACTGAGCCTGGTTGGTCCGGCAACC
CGTGAAGAAGCAATGGGTTATTTTGCAAATTGGAATCCGGGTCTGCGTGCAGTTCTGGGTTTTATGCCGGAAAATATTGAT
CGTTGGGCAATGTTCGATACCTATGATTATCCGGCACCGTTTTTTAGCCGTGGTAAAATTTGTCTGGTTGGTGATGCAGCA
CATGCAGCAGTTCCGCATCATGGTGCCGGTGCATGTATTGGTATTGAAGATGCACTGTGTGCAACCGTTCTGCTGGCAGAA
GTTTTTGTTAGCACCCGTGGCAAAAGCAGCATTGTTCGTAATCGTGCAATTGCCGCAGCATTTGGTAGCTTTAATGCAGTG
CGTCGTGTTCGTGCACAGTGGTTTGTTGATAGCAGCCGTCGTGTTTGTGATCTGTATCAACAGCCGGAATGGGCAGATCCG
CAGAAACGTATTAAAGCCGAAAATTGCTTCGAAGAGATTAAAGATCGCAGCCATAAAATCTGGCACTTCGATTATAACTCC
ATGCTGCAAGAAGCCATCGAAAAATATCGTCATAATATGGGCAGCTAA
```

```
MPGSLIDTRQQPLSVGIVGGGIIGVILAAGLVRRGIDVKVFEQARGFREIGAGMAFTANAVRCMEMLDPAIVWALRSSGAV
PISIGDHQAEARDYLRWVDGYHESSKRLYQLDAGIRGFEACRRDQFLEALVKVLPEGIVECQKRLQKIHEKNETEKVTLEF
ADGTFAHVDCVIGADGIRSRVRQHLFGEDSPYSHPHYSHKFAFRGLITMENAISALGEDKARTLNMHVGPNAHLIHYPVAN
ETMVNIAAFVSDPEEWPDKLSLVGPATREEAMGYFANWNPGLRAVLGFMPENIDRWAMFDTYDYPAPFFSRGKICLVGDAA
HAAVPHHGAGACIGIEDALCATVLLAEVFVSTRGKSSIVRNRAIAAAFGSFNAVRRVRAQWFVDSSRRVCDLYQQPEWADP
QKRIKAENCFEEIKDRSHKIWHFDYNSMLQEAIEKYRHNMGS
```

XenC sequences

```
ATGGGCAGTTTGACCGACAATGCCGCGATTCCCACCGTGGATATCTCGGCGTTCTTAGATCCTAATGCCTCGCAGGAGGCA
CGTCAGGACGTTGTGAATGCGATGTCGAATGCCTGCCACGTATATGGTTTTTTCAATCTGGCGGGTCATGGCATTCCACAA
GAGACCTTGCGCGAAGCATTCGAACTTAATAAGATGTTTTTCGCCTTGCCAGAGGAATCAAAAAAAGAAGTGTTGATTAGT
AAGTCAATCGGACAATCATTCCGTGGTTACGAGCCGCCCGGCATCCAGACACATCACAAGGGCCTTCTTCCCGATATTAAA
GAGACGTTCATGGTCGGGCGTGAAGTACCGCTTGACGACCCTGATTGTGGCACATTCTCAACGGGTCCCAACCTTTGGCCC
TCATCTTTACCGAAAGAGAAATTTCAGGACCGCATCATGGCTTACCAGGGTAGCATGTTAGAACTTGTAAAGAATATTCTT
GCTATTTTGGCACAAGGGCTGCCAAAGGAATGGGGATGCTCACCGACCGTTTTCAACTCTTTATTGGATAAGCCGAGTATT
CCAATGCGTTTCTTACACTACGCCCCCGTCCCCTCACAACTTGAAGATGTACGCCAGTTTGGGGTAGCTGACCACACTGAC
TTTGGTTGTGTAAGTATTTTGTTGCAGGAGCCCGGAACTTCGGGCTTAGAGGTCTACTATCCCCCGTCGGACTCGTGGATT
CCTGTCCCAGTTATCGACGATGGATTCGTGATCAACATGGGTGATATGATGCAACGTTACACCGGGGGATATTACCGCTCA
GCTCGCCATCGCGTCCTTACAAACCGCGAGAAACATCGTCACAGTGTGGCGTTTTTTCTGAATGGGAACTTGGGATTAAAG
```

GCGAAGGCTTTAGATGGCAGCGAAACGGAGACAGTAGTTGGAGATTGGATTCGCGGGCGTCTGATTGACACTATGGGACAG
ACGGGGAAGTTATTGCAGCGTGAGAGTCCGAAACCGGTAGTACTGCCG

MGSLTDNAAIPTVDISAFLDPNASQEARQDVVNAMSNACHVYGFFNLAGHGIPQETLREAFELNKMFFALPEESKKEVLIS
KSIGQSFRGYEPPGIQTHHKGLLPDIKETFMVGREVPLDDPDCGTFSTGPNLWPSSLPKEKFQDRIMAYQGSMLELVKNIL
AILAQGLPKEWGCSPTVFNSLLDKPSIPMRFLHYAPVPSQLEDVRQFGVADHTDFGCVSILLQEPGTSGLEVYYPPSDSWI
PVPVIDDGFVINMGDMMQRYTGGYYRSARHRVLTNREKHRHSVAFFLNGNLGLKAKALDGSETETVVGDWIRGRLIDTMGQ
TGKLLQRESPKPVVLP

XenC_W313Y sequences

ATGGGCAGTTTGACCGACAATGCCGCGATTCCCACCGTGGATATCTCGGCGTTCTTAGATCCTAATGCCTCGCAGGAGGCA
CGTCAGGACGTTGTGAATGCGATGTCGAATGCCTGCCACGTATATGGTTTTTTCAATCTGGCGGGTCATGGCATTCCACAA
GAGACCTTGCGCGAAGCATTCGAACTTAATAAGATGTTTTTCGCCTTGCCAGAGGAATCAAAAAAAGAAGTGTTGATTAGT
AAGTCAATCGGACAATCATTCCGTGGTTACGAGCCGCCCGGCATCCAGACACATCACAAGGGCCTTCTTCCCGATATTAAA
GAGACGTTCATGGTCGGGCGTGAAGTACCGCTTGACGACCCTGATTGTGGCACATTCTCAACGGGTCCCAACCTTTGGCCC
TCATCTTTACCGAAAGAGAAATTTCAGGACCGCATCATGGCTTACCAGGGTAGCATGTTAGAACTTGTAAAGAATATTCTT
GCTATTTTGGCACAAGGGCTGCCAAAGGAATGGGGATGCTCACCGACCGTTTTCAACTCTTTATTGGATAAGCCGAGTATT
CCAATGCGTTTCTTACACTACGCCCCCGTCCCCTCACAACTTGAAGATGTACGCCAGTTTGGGGTAGCTGACCACACTGAC
TTTGGTTGTGTAAGTATTTTGTTGCAGGAGCCCGGAACTTCGGGCTTAGAGGTCTACTATCCCCCGTCGGACTCGTGGATT
CCTGTCCCAGTTATCGACGATGGATTCGTGATCAACATGGGTGATATGATGCAACGTTACACCGGGGGATATTACCGCTCA
GCTCGCCATCGCGTCCTTACAAACCGCGAGAAACATCGTCACAGTGTGGCGTTTTTTCTGAATGGGAACTTGGGATTAAAG
GCGAAGGCTTTAGATGGCAGCGAAACGGAGACAGTAGTTGGAGATTATATTCGCGGGCGTCTGATTGACACTATGGGACAG
ACGGGGAAGTTATTGCAGCGTGAGAGTCCGAAACCGGTAGTACTGCCG

MGSLTDNAAIPTVDISAFLDPNASQEARQDVVNAMSNACHVYGFFNLAGHGIPQETLREAFELNKMFFALPEESKKEVLIS
KSIGQSFRGYEPPGIQTHHKGLLPDIKETFMVGREVPLDDPDCGTFSTGPNLWPSSLPKEKFQDRIMAYQGSMLELVKNIL
AILAQGLPKEWGCSPTVFNSLLDKPSIPMRFLHYAPVPSQLEDVRQFGVADHTDFGCVSILLQEPGTSGLEVYYPPSDSWI
PVPVIDDGFVINMGDMMQRYTGGYYRSARHRVLTNREKHRHSVAFFLNGNLGLKAKALDGSETETVVGDYIRGRLIDTMGQ
TGKLLQRESPKPVVLP

XenC_W313Y/T110C sequences

ATGGGCAGTTTGACCGACAATGCCGCGATTCCCACCGTGGATATCTCGGCGTTCTTAGATCCTAATGCCTCGCAGGAGGCA
CGTCAGGACGTTGTGAATGCGATGTCGAATGCCTGCCACGTATATGGTTTTTTCAATCTGGCGGGTCATGGCATTCCACAA
GAGACCTTGCGCGAAGCATTCGAACTTAATAAGATGTTTTTCGCCTTGCCAGAGGAATCAAAAAAAGAAGTGTTGATTAGT
AAGTCAATCGGACAATCATTCCGTGGTTACGAGCCGCCCGGCATCCAGACACATCACAAGGGCCTTCTTCCCGATATTAAA
GAGTGTTTCATGGTCGGGCGTGAAGTACCGCTTGACGACCCTGATTGTGGCACATTCTCAACGGGTCCCAACCTTTGGCCC
TCATCTTTACCGAAAGAGAAATTTCAGGACCGCATCATGGCTTACCAGGGTAGCATGTTAGAACTTGTAAAGAATATTCTT
GCTATTTTGGCACAAGGGCTGCCAAAGGAATGGGGATGCTCACCGACCGTTTTCAACTCTTTATTGGATAAGCCGAGTATT
CCAATGCGTTTCTTACACTACGCCCCCGTCCCCTCACAACTTGAAGATGTACGCCAGTTTGGGGTAGCTGACCACACTGAC
TTTGGTTGTGTAAGTATTTTGTTGCAGGAGCCCGGAACTTCGGGCTTAGAGGTCTACTATCCCCCGTCGGACTCGTGGATT
CCTGTCCCAGTTATCGACGATGGATTCGTGATCAACATGGGTGATATGATGCAACGTTACACCGGGGGATATTACCGCTCA
GCTCGCCATCGCGTCCTTACAAACCGCGAGAAACATCGTCACAGTGTGGCGTTTTTTCTGAATGGGAACTTGGGATTAAAG
GCGAAGGCTTTAGATGGCAGCGAAACGGAGACAGTAGTTGGAGATTATATTCGCGGGCGTCTGATTGACACTATGGGACAG
ACGGGGAAGTTATTGCAGCGTGAGAGTCCGAAACCGGTAGTACTGCCG

MGSLTDNAAIPTVDISAFLDPNASQEARQDVVNAMSNACHVYGFFNLAGHGIPQETLREAFELNKMFFALPEESKKEVLIS
KSIGQSFRGYEPPGIQTHHKGLLPDIKECFMVGREVPLDDPDCGTFSTGPNLWPSSLPKEKFQDRIMAYQGSMLELVKNIL
AILAQGLPKEWGCSPTVFNSLLDKPSIPMRFLHYAPVPSQLEDVRQFGVADHTDFGCVSILLQEPGTSGLEVYYPPSDSWI
PVPVIDDGFVINMGDMMQRYTGGYYRSARHRVLTNREKHRHSVAFFLNGNLGLKAKALDGSETETVVGDYIRGRLIDTMGQ
TGKLLQRESPKPVVLP

XenC_W313Y/T110C/K101Q sequences

ATGGGCAGTTTGACCGACAATGCCGCGATTCCCACCGTGGATATCTCGGCGTTCTTAGATCCTAATGCCTCGCAGGAGGCA
CGTCAGGACGTTGTGAATGCGATGTCGAATGCCTGCCACGTATATGGTTTTTTCAATCTGGCGGGTCATGGCATTCCACAA
GAGACCTTGCGCGAAGCATTCGAACTTAATAAGATGTTTTTCGCCTTGCCAGAGGAATCAAAAAAAGAAGTGTTGATTAGT
AAGTCAATCGGACAATCATTCCGTGGTTACGAGCCGCCCGGCATCCAGACACATCACCAGGGCCTTCTTCCCGATATTAAA
GAGTGTTTCATGGTCGGGCGTGAAGTACCGCTTGACGACCCTGATTGTGGCACATTCTCAACGGGTCCCAACCTTTGGCCC
TCATCTTTACCGAAAGAGAAATTTCAGGACCGCATCATGGCTTACCAGGGTAGCATGTTAGAACTTGTAAAGAATATTCTT
GCTATTTTGGCACAAGGGCTGCCAAAGGAATGGGGATGCTCACCGACCGTTTTCAACTCTTTATTGGATAAGCCGAGTATT
CCAATGCGTTTCTTACACTACGCCCCCGTCCCCTCACAACTTGAAGATGTACGCCAGTTTGGGGTAGCTGACCACACTGAC
TTTGGTTGTGTAAGTATTTTGTTGCAGGAGCCCGGAACTTCGGGCTTAGAGGTCTACTATCCCCCGTCGGACTCGTGGATT
CCTGTCCCAGTTATCGACGATGGATTCGTGATCAACATGGGTGATATGATGCAACGTTACACCGGGGGATATTACCGCTCA
GCTCGCCATCGCGTCCTTACAAACCGCGAGAAACATCGTCACAGTGTGGCGTTTTTTCTGAATGGGAACTTGGGATTAAAG
GCGAAGGCTTTAGATGGCAGCGAAACGGAGACAGTAGTTGGAGATTATATTCGCGGGCGTCTGATTGACACTATGGGACAG
ACGGGGAAGTTATTGCAGCGTGAGAGTCCGAAACCGGTAGTACTGCCG

MGSLTDNAAIPTVDISAFLDPNASQEARQDVVNAMSNACHVYGFFNLAGHGIPQETLREAFELNKMFFALPEESKKEVLIS
KSIGQSFRGYEPPGIQTHHQGLLPDIKECFMVGREVPLDDPDCGTFSTGPNLWPSSLPKEKFQDRIMAYQGSMLELVKNIL
AILAQGLPKEWGCSPTVFNSLLDKPSIPMRFLHYAPVPSQLEDVRQFGVADHTDFGCVSILLQEPGTSGLEVYYPPSDSWI
PVPVIDDGFVINMGDMMQRYTGGYYRSARHRVLTNREKHRHSVAFFLNGNLGLKAKALDGSETETVVGDYIRGRLIDTMGQ
TGKLLQRESPKPVVLP

Anc1 sequences

ATGACCACCACCGCACCGGAAGCAATTCCGACCGTTGATATTAGCGCATGGCTGAGCCCGAATGCAAGCGAAGAAGCAAAA
CAGCAGGTTGTTGAAGCAATGCGTCATGCATGTACCACCTATGGTTTCTTTTATCTGGTTGGTCATGGTGTTAGTCCGGAA
GAACAGCAGAAAGCACTGGATTGTACCAAACTGTTTTTCGCACTGCCGATGGAAGAACGTATGGAAGTTTGGATTGGTAAA
AGCATGGGTAAAAGCTTTCGTGGTTATGAACCGCCTGGTATTCAGACCCATCATGAAGGTCTGCTGCCGGATACCAAAGAA
ACCTTTATGGTTGGTGCCGAAGTTCCGGCAGATGATCCGGATGCAGGCACCTTTAGCACCGGTCCGAATCTGTGGCCGAAA
AGCCTGCCGGATGAAGAATTTCGTACACCGGTTATGGAATATCAGGCCAAAATGGTTGAACTGGTTAAAGTGCTGCTGAAA
ATTCTGGCACGTGGTCTGCCGAAAGAATGGAATTGTCCGCCTGATGTTTTTGATGAACTGGCAGTTAATCCGAGCATTCCG
ATGCGTCTGCTGCATTATGCACCGCAGCCGGTTCGTGATGATCGTCAGTTTGGTGTTGCAGATCATACCGATTTTGGTTGC
ATTACCATTCTGCTGCAAGAACCGGGTACAAAAGGTCTGGAAGTGTGGTATCCGCCTACCGAAACCTGGATTCCGGTTCCG
GTTAAAGAAGGTGCATACGTTATTAACATGGGCGACATGATGCAGAAATGGACCGGTGGTTATTATCGTAGCGCACGTCAT
CGTGTTATTACCAGCAGCGATAAACATCGTTATAGCGTTGCATTTTTCCTGAACGGTAACCTGAAACTGAAATGTAAAGCC
CTGGATGGTAGCGGTGCAGAAACCGTTGTTGGTGAACATATTCGTCAGCGTCTGATTGAAACCATGGGTGAAACCGGTAAA
ATGCTGAAA

MTTTAPEAIPTVDISAWLSPNASEEAKQQVVEAMRHACTTYGFFYLVGHGVSPEEQQKALDCTKLFFALPMEERMEVWIGK
SMGKSFRGYEPPGIQTHHEGLLPDTKETFMVGAEVPADDPDAGTFSTGPNLWPKSLPDEEFRTPVMEYQAKMVELVKVLLK
ILARGLPKEWNCPPDVFDELAVNPSIPMRLLHYAPQPVRDDRQFGVADHTDFGCITILLQEPGTKGLEVWYPPTETWIPVP
VKEGAYVINMGDMMQKWTGGYYRSARHRVITSSDKHRYSVAFFLNGNLKLKCKALDGSGAETVVGEHIRQRLIETMGETGK
MLK

Anc1_D210G sequences

ATGACCACCACCGCACCGGAAGCAATTCCGACCGTTGATATTAGCGCATGGCTGAGCCCGAATGCAAGCGAAGAAGCAAAA
CAGCAGGTTGTTGAAGCAATGCGTCATGCATGTACCACCTATGGTTTCTTTTATCTGGTTGGTCATGGTGTTAGTCCGGAA
GAACAGCAGAAAGCACTGGATTGTACCAAACTGTTTTTCGCACTGCCGATGGAAGAACGTATGGAAGTTTGGATTGGTAAA
AGCATGGGTAAAAGCTTTCGTGGTTATGAACCGCCTGGTATTCAGACCCATCATGAAGGTCTGCTGCCGGATACCAAAGAA
ACCTTTATGGTTGGTGCCGAAGTTCCGGCAGATGATCCGGATGCAGGCACCTTTAGCACCGGTCCGAATCTGTGGCCGAAA
AGCCTGCCGGATGAAGAATTTCGTACACCGGTTATGGAATATCAGGCCAAAATGGTTGAACTGGTTAAAGTGCTGCTGAAA
ATTCTGGCACGTGGTCTGCCGAAAGAATGGAATTGTCCGCCTGATGTTTTTGATGAACTGGCAGTTAATCCGAGCATTCCG
ATGCGTCTGCTGCATTATGCACCGCAGCCGGTTCGTGATGATCGTCAGTTTGGTGTTGCAGGGCATACCGATTTTGGTTGC

ATTACCATTCTGCTGCAAGAACCGGGTACAAAAGGTCTGGAAGTGTGGTATCCGCCTACCGAAACCTGGATTCCGGTTCCG
GTTAAAGAAGGTGCATACGTTATTAACATGGGCGACATGATGCAGAAATGGACCGGTGGTTATTATCGTAGCGCACGTCAT
CGTGTTATTACCAGCAGCGATAAACATCGTTATAGCGTTGCATTTTTCCTGAACGGTAACCTGAAACTGAAATGTAAAGCC
CTGGATGGTAGCGGTGCAGAAACCGTTGTTGGTGAACATATTCGTCAGCGTCTGATTGAAACCATGGGTGAAACCGGTAAA
ATGCTGAAA

MTTTAPEAIPTVDISAWLSPNASEEAKQQVVEAMRHACTTYGFFYLVGHGVSPEEQQKALDCTKLFFALPMEERMEVWIGK
SMGKSFRGYEPPGIQTHHEGLLPDTKETFMVGAEVPADDPDAGTFSTGPNLWPKSLPDEEFRTPVMEYQAKMVELVKVLLK
ILARGLPKEWNCPPDVFDELAVNPSIPMRLLHYAPQPVRDDRQFGVAGHTDFGCITILLQEPGTKGLEVWYPPTETWIPVP
VKEGAYVINMGDMMQKWTGGYYRSARHRVITSSDKHRYSVAFFLNGNLKLKCKALDGSGAETVVGEHIRQRLIETMGETGK
MLK

Anc1_D210G/E25G/G94E/F206M sequences

ATGACCACCACCGCACCGGAAGCAATTCCGACCGTTGATATTAGCGCATGGCTGAGCCCGAATGCAAGCGAAGGGGCAAAA
CAGCAGGTTGTTGAAGCAATGCGTCATGCATGTACCACCTATGGTTTCTTTTATCTGGTTGGTCATGGTGTTAGTCCGGAA
GAACAGCAGAAAGCACTGGATTGTACCAAACTGTTTTTCGCACTGCCGATGGAAGAACGTATGGAAGTTTGGATTGGTAAA
AGCATGGGTAAAAGCTTTCGTGGTTATGAACCGCCTGAGATTCAGACCCATCATGAAGGTCTGCTGCCGGATACCAAAGAA
ACCTTTATGGTTGGTGCCGAAGTTCCGGCAGATGATCCGGATGCAGGCACCTTTAGCACCGGTCCGAATCTGTGGCCGAAA
AGCCTGCCGGATGAAGAATTTCGTACACCGGTTATGGAATATCAGGCCAAAATGGTTGAACTGGTTAAAGTGCTGCTGAAA
ATTCTGGCACGTGGTCTGCCGAAAGAATGGAATTGTCCGCCTGATGTTTTTGATGAACTGGCAGTTAATCCGAGCATTCCG
ATGCGTCTGCTGCATTATGCACCGCAGCCGGTTCGTGATGATCGTCAGATGGGTGTTGCAGGGCATACCGATTTTGGTTGC
ATTACCATTCTGCTGCAAGAACCGGGTACAAAAGGTCTGGAAGTGTGGTATCCGCCTACCGAAACCTGGATTCCGGTTCCG
GTTAAAGAAGGTGCATACGTTATTAACATGGGCGACATGATGCAGAAATGGACCGGTGGTTATTATCGTAGCGCACGTCAT
CGTGTTATTACCAGCAGCGATAAACATCGTTATAGCGTTGCATTTTTCCTGAACGGTAACCTGAAACTGAAATGTAAAGCC
CTGGATGGTAGCGGTGCAGAAACCGTTGTTGGTGAACATATTCGTCAGCGTCTGATTGAAACCATGGGTGAAACCGGTAAA
ATGCTGAAA

MTTTAPEAIPTVDISAWLSPNASEGAKQQVVEAMRHACTTYGFFYLVGHGVSPEEQQKALDCTKLFFALPMEERMEVWIGK
SMGKSFRGYEPPEIQTHHEGLLPDTKETFMVGAEVPADDPDAGTFSTGPNLWPKSLPDEEFRTPVMEYQAKMVELVKVLLK
ILARGLPKEWNCPPDVFDELAVNPSIPMRLLHYAPQPVRDDRQMGVAGHTDFGCITILLQEPGTKGLEVWYPPTETWIPVP
VKEGAYVINMGDMMQKWTGGYYRSARHRVITSSDKHRYSVAFFLNGNLKLKCKALDGSGAETVVGEHIRQRLIETMGETGK
MLK

Anc1_D210G/E25G/G94E/F206M/M75E sequences

ATGACCACCACCGCACCGGAAGCAATTCCGACCGTTGATATTAGCGCATGGCTGAGCCCGAATGCAAGCGAAGGGGCAAAA
CAGCAGGTTGTTGAAGCAATGCGTCATGCATGTACCACCTATGGTTTCTTTTATCTGGTTGGTCATGGTGTTAGTCCGGAA
GAACAGCAGAAAGCACTGGATTGTACCAAACTGTTTTTCGCACTGCCGATGGAAGAACGTGAGGAAGTTTGGATTGGTAAA
AGCATGGGTAAAAGCTTTCGTGGTTATGAACCGCCTGAGATTCAGACCCATCATGAAGGTCTGCTGCCGGATACCAAAGAA
ACCTTTATGGTTGGTGCCGAAGTTCCGGCAGATGATCCGGATGCAGGCACCTTTAGCACCGGTCCGAATCTGTGGCCGAAA
AGCCTGCCGGATGAAGAATTTCGTACACCGGTTATGGAATATCAGGCCAAAATGGTTGAACTGGTTAAAGTGCTGCTGAAA
ATTCTGGCACGTGGTCTGCCGAAAGAATGGAATTGTCCGCCTGATGTTTTTGATGAACTGGCAGTTAATCCGAGCATTCCG
ATGCGTCTGCTGCATTATGCACCGCAGCCGGTTCGTGATGATCGTCAGATGGGTGTTGCAGGGCATACCGATTTTGGTTGC
ATTACCATTCTGCTGCAAGAACCGGGTACAAAAGGTCTGGAAGTGTGGTATCCGCCTACCGAAACCTGGATTCCGGTTCCG
GTTAAAGAAGGTGCATACGTTATTAACATGGGCGACATGATGCAGAAATGGACCGGTGGTTATTATCGTAGCGCACGTCAT
CGTGTTATTACCAGCAGCGATAAACATCGTTATAGCGTTGCATTTTTCCTGAACGGTAACCTGAAACTGAAATGTAAAGCC
CTGGATGGTAGCGGTGCAGAAACCGTTGTTGGTGAACATATTCGTCAGCGTCTGATTGAAACCATGGGTGAAACCGGTAAA
ATGCTGAAA

MTTTAPEAIPTVDISAWLSPNASEGAKQQVVEAMRHACTTYGFFYLVGHGVSPEEQQKALDCTKLFFALPMEEREEVWIGK
SMGKSFRGYEPPEIQTHHEGLLPDTKETFMVGAEVPADDPDAGTFSTGPNLWPKSLPDEEFRTPVMEYQAKMVELVKVLLK
ILARGLPKEWNCPPDVFDELAVNPSIPMRLLHYAPQPVRDDRQMGVAGHTDFGCITILLQEPGTKGLEVWYPPTETWIPVP

VKEGAYVINMGDMMQKWTGGYYRSARHRVITSSDKHRYSVAFFLNGNLKLKCKALDGSGAETVVGEHIRQRLIETMGETGK
MLK

Anc1_D210G/G25E/G94E/F206M/M75 sequences

ATGACCACCACCGCACCGGAAGCAATTCCGACCGTTGATATTAGCGCATGGCTGAGCCCGAATGCAAGCGAAGAAGCAAAA
CAGCAGGTTGTTGAAGCAATGCGTCATGCATGTACCACCTATGGTTTCTTTTATCTGGTTGGTCATGGTGTTAGTCCGGAA
GAACAGCAGAAAGCACTGGATTGTACCAAACTGTTTTTCGCACTGCCGATGGAAGAACGTGAGGAAGTTTGGATTGGTAAA
AGCATGGGTAAAAGCTTTCGTGGTTATGAACCGCCTGAGATTCAGACCCATCATGAAGGTCTGCTGCCGGATACCAAAGAA
ACCTTTATGGTTGGTGCCGAAGTTCCGGCAGATGATCCGGATGCAGGCACCTTTAGCACCGGTCCGAATCTGTGGCCGAAA
AGCCTGCCGGATGAAGAATTTCGTACACCGGTTATGGAATATCAGGCCAAAATGGTTGAACTGGTTAAAGTGCTGCTGAAA
ATTCTGGCACGTGGTCTGCCGAAAGAATGGAATTGTCCGCCTGATGTTTTTGATGAACTGGCAGTTAATCCGAGCATTCCG
ATGCGTCTGCTGCATTATGCACCGCAGCCGGTTCGTGATGATCGTCAGATGGGTGTTGCAGGGCATACCGATTTTGGTTGC
ATTACCATTCTGCTGCAAGAACCGGGTACAAAAGGTCTGGAAGTGTGGTATCCGCCTACCGAAACCTGGATTCCGGTTCCG
GTTAAAGAAGGTGCATACGTTATTAACATGGGCGACATGATGCAGAAATGGACCGGTGGTTATTATCGTAGCGCACGTCAT
CGTGTTATTACCAGCAGCGATAAACATCGTTATAGCGTTGCATTTTTCCTGAACGGTAACCTGAAACTGAAATGTAAAGCC
CTGGATGGTAGCGGTGCAGAAACCGTTGTTGGTGAACATATTCGTCAGCGTCTGATTGAAACCATGGGTGAAACCGGTAAA
ATGCTGAAA

MTTTAPEAIPTVDISAWLSPNASEEAKQQVVEAMRHACTTYGFFYLVGHGVSPEEQQKALDCTKLFFALPMEEREEVWIGK
SMGKSFRGYEPPEIQTHHEGLLPDTKETFMVGAEVPADDPDAGTFSTGPNLWPKSLPDEEFRTPVMEYQAKMVELVKVLLK
ILARGLPKEWNCPPDVFDELAVNPSIPMRLLHYAPQPVRDDRQMGVAGHTDFGCITILLQEPGTKGLEVWYPPTETWIPVP
VKEGAYVINMGDMMQKWTGGYYRSARHRVITSSDKHRYSVAFFLNGNLKLKCKALDGSGAETVVGEHIRQRLIETMGETGK
MLK

Preparative-scale flavin-dependent monooxygenase (FDMO) protein production. Chemically competent BL21(DE3) cells were transformed with a pET151 plasmid containing *tropB* using a standard heat-shock protocol. Overexpression of *tropB* was achieved using 4% glycerol (v/v) Terrific Broth (TB) in 2.8 L flasks. 500 mL portions of autoclaved media were inoculated with 5 mL of overnight culture prepared from a single colony in Luria Broth (LB) and 100 μg/mL ampicillin (Gold Biotechnology). Cultures were grown at 37 ºC and 200 rpm until the optical density (at 600 nm) reached 0.6–0.8. The cultures were then cooled to 20 ºC and gene expression was induced with 0.5 mM isopropyl-β-D-1-thiogalactopyranoside (IPTG, Gold Biotechnology). Expression was continued at 20 ºC overnight (approx. 18 h) at 200 rpm. The typical yield for one 500 mL culture cell pellet (wet cell pellet) was ~20 g.

Preparative-scale α-ketoglutarate non-heme dependent iron (NHI) enzyme protein production. Chemically competent BL21(DE3) cells were transformed with a pET28a plasmid containing each NHI gene using a standard heat-shock protocol. Overexpression of each NHI gene was achieved using 4% glycerol (v/v) Terrific Broth (TB) in 2.8 L flasks. 500 mL portions of autoclaved media were inoculated with 5 mL of overnight culture prepared from a single colony in Luria Broth (LB) and 50 μg/mL kanamycin (Gold Biotechnology). Cultures were grown at 37 ºC and 200 rpm until the optical density (at 600 nm) reached 0.6–0.8. The cultures were then cooled to 20 ºC and gene expression was induced with 0.5 mM IPTG. Expression was continued at 20 ºC overnight (approx. 18 h) at 200 rpm. The typical yield for one 500 mL culture cell pellet (wet cell pellet) was ~20 g.

Preparative-scale preparation of clarified cell lysate. 50–60 g of cell pellet containing TropB or an NHI enzyme was resuspended to a concentration of 200 mg/mL in lysis buffer containing 50 mM TES (pH 7.5) and 100 μM phenylmethylsulfonyl fluoride (PMSF). The resuspended cells were homogenized using a handheld dounce homogenizer. Cells were lysed by sonication of the total cell lysate in 100 mL batches on ice. Each cycle of sonication was 10 s sonication, followed by a 30 s rest period, for a total time of 5 min at 40% power. The total cell lysate was centrifuged at 30,000 x g for 30 min and the supernatant was removed. Aliquots of lysate were used fresh for enzymatic reactions or were flash frozen in liquid nitrogen and stored at -80 ºC until needed.

Purification of **α-ketoglutarate non-heme dependent iron (NHI) enzymes.** 20 g of pellet were resuspended in 100 mL of lysis buffer containing 50 mM Tris pH 7.5, 300 mM NaCl, 10 mM imidazole, and 10% v/v glycerol. To the lysis buffer, phenylmethylsulfonyl fluoride (PMSF) in ispropanol was added to a final concentration of 100 µM. The resuspended cells were homogenized using a handheld dounce homogenizer. Cells were lysed by sonication of the total cell lysate in 100 mL batches on ice. Each cycle of sonication was 10 s sonication, followed by a 30 s rest period, for a total time of 5 min at 40% power. The total cell lysate was centrifuged at 30,000 x g for 30 min and the supernatant was removed. The cell lysate was then batch bound to 2–3 mL of Ni-NTA resin (ThermoFisher) for 2 h at 4 ºC with continuous gentle rocking. The cell lysate containing the Ni-NTA resin was filtered through a fritted 12 mL plastic column (Gold Biotechnologies) to retain the Ni-NTA resin. The resin was then washed with 10 mL of wash buffer containing 50 mM Tris pH 7.5, 300 mM NaCl, 20 mM imidazole and 10% v/v glycerol. The protein was then eluted with 5 mL of elution buffer containing 50 mM Tris pH 7.5, 300 mM NaCl, 500 mM imidazole, and 10% glycerol. The resulting eluent was concentrated using a 30 kDa molecular weight cutoff ultrafiltration device (Amicon) to a final volume of 2.5 mL. The concentrated sample was then desalted using a PD-10 desalting column that was preequilibrated with storage buffer containing 50 mM Tris pH 7.5, 300 mM NaCl, and 10% glycerol. The protein was eluted using 3.5 mL of storage buffer, aliquoted and flash frozen with liquid nitrogen to store at –80 ºC until needed.

Figure S1. SDS PAGE gels of the purified NHI variants generated during the directed evolution campaigns of XenC and Anc1 stained with Coomassie Blue stain.

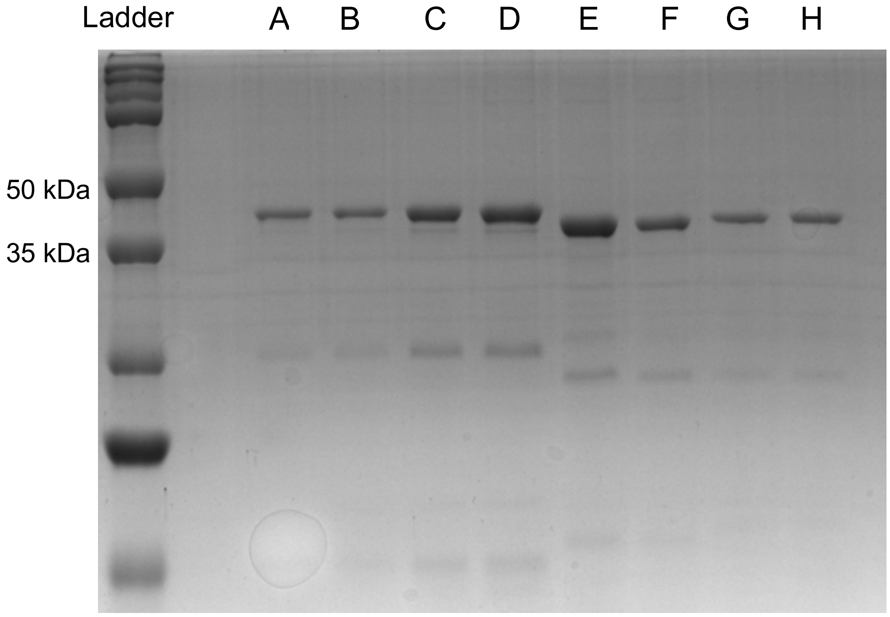

Lane	Sample
A	XenC
B	XenCRd1
C	XenCRd2
D	XenCRd3
E	Anc1
F	Anc1Rd1
G	Anc1Rd2
H	Anc1Rd3

Quantification of soluble protein. Clarified lysate of all the variants generated was diluted 10-fold with 50 mM TES pH 7.5 before adding an equal volume of loading dye (2x Laemmli Sample Buffer). The samples were heated at 95 ºC for 10 min before aliquoting 10 µL into the wells of a 10% Polyacrylamide SDS-PAGE gel. The electrophoresis was set up to run for 45 min at 180 V and 400 mA, and the InVision His-tag In-Gel Stain protocol provided by Novex was followed to selectively stain His-tagged proteins. After staining, the gels were visualized using UV302 on an Azure c600 Gel Imager and ImageJ was used to determine the relative intensity of each band.

Figure S2. SDS PAGE gels of the lysate of the variants generated during the directed evolution campaigns of XenC and Anc1 stained with Coomassie Blue (left) stain and His-tag selective stain (right) to determine protein expression and band intensity.

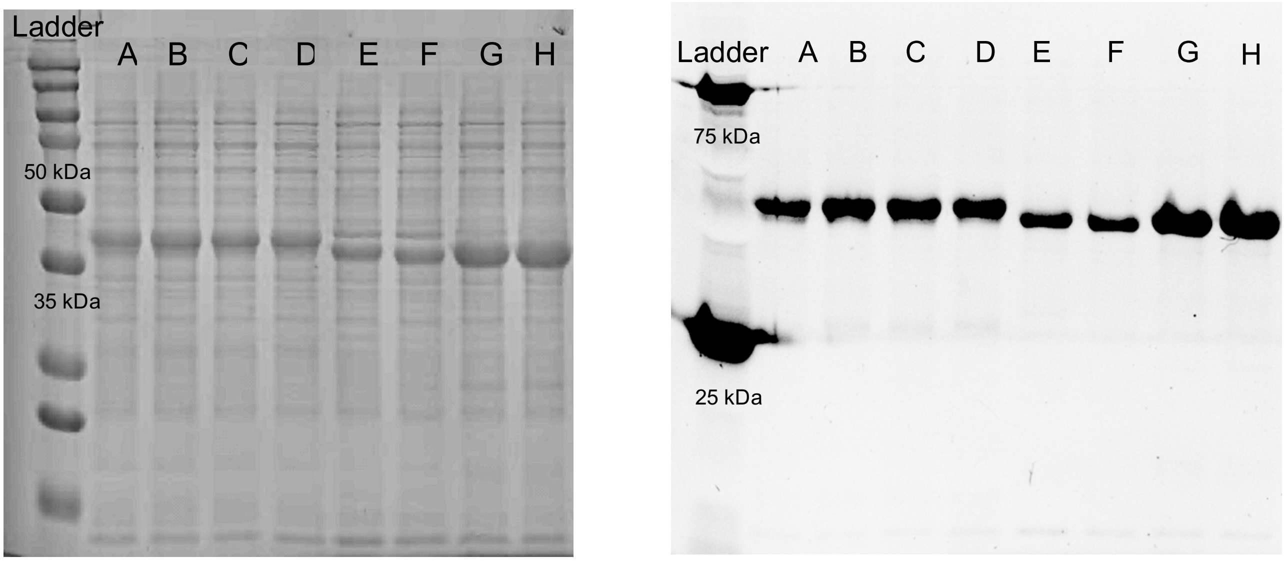

Table S1. Intensity of protein bands on His-tag selective stained gel and fold improvement in soluble protein over wt-XenC

Lane	Enzyme	Band Intensity	Fold Improvement
A	wt-XenC	5864.861	1.00
B	XenC_W313Y	7319.154	1.25
C	XenC_W313Y/T110C	7363.154	1.26
D	XenC_W313Y/T110C/K101Q	6998.033	1.19
E	Anc1	4425.79	0.75
F	Anc1_D210G	3940.184	0.67
G	Anc1_D210G/E25G/Q94E/F206M	11450.075	1.95
H	Anc1_D210G/E25G/Q94E/F206M/M75E	13062.075	2.23

Figure S3. Protein sequence alignment of wt-XenC, Anc1, and TropC. Conserved regions shown in dark blue.

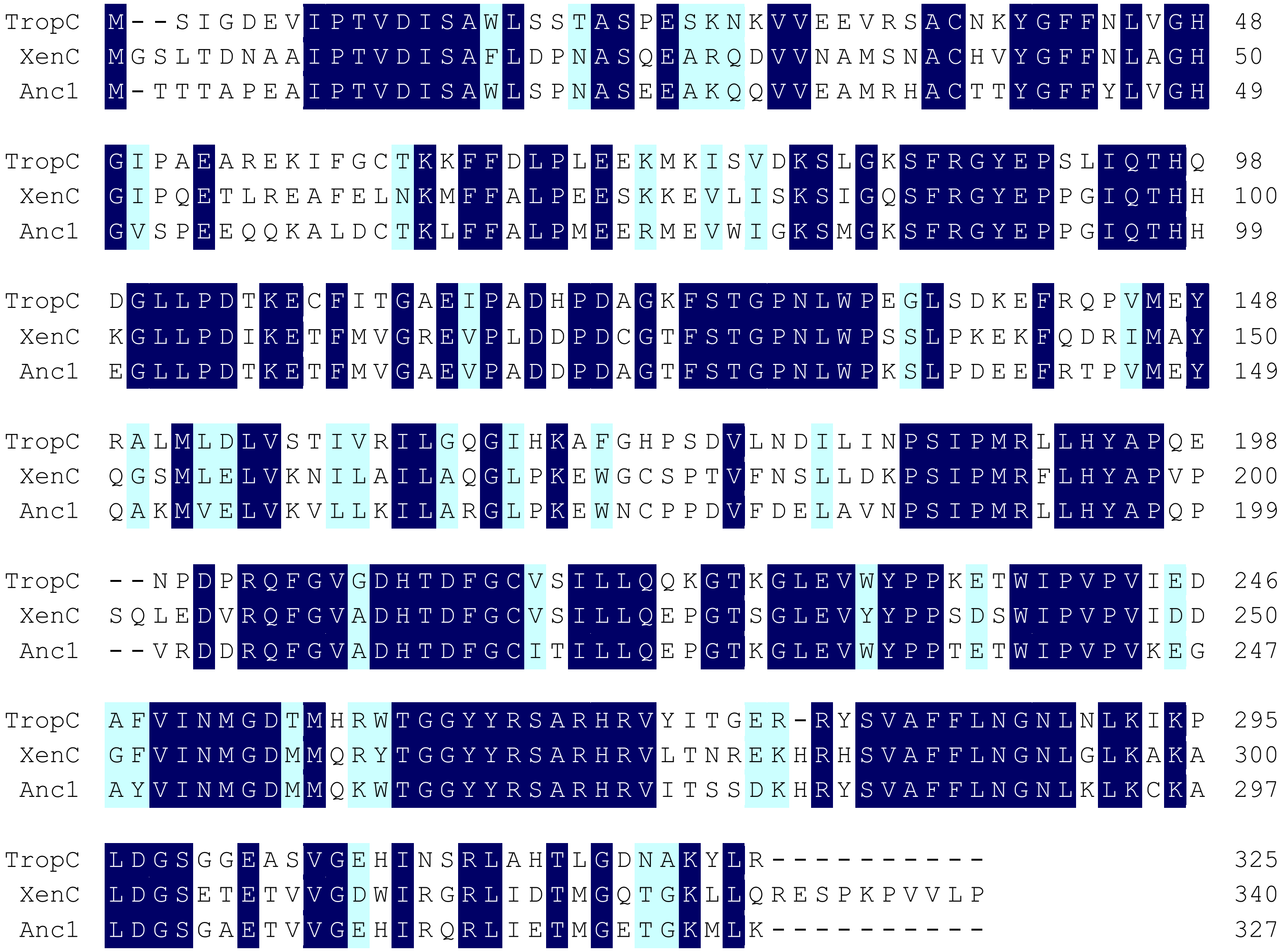

Figure S4. Protein sequence identity (% ID) between TropC, XenC, and Anc1.

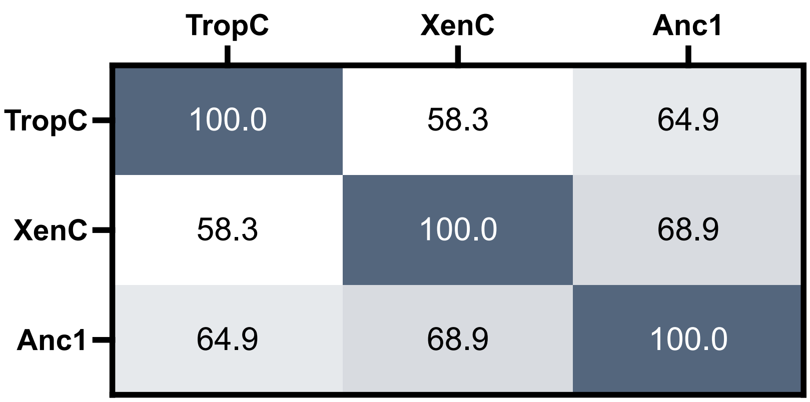

III. Directed Evolution of NHI enzymes

Generation of AlphaFold Structures. The AlphaFold predicted models of XenC and Anc1 were generated using the ColabFold v1.5.5: AlphaFold2 using MMseqs2 Google Colab notebook.[5] The highest-ranking models (Figure S5) were used for target residue selection for our evolution campaigns.

Figure S5. (A) Crystal structure of TropC (PDB 6XJJ) with iron (orange sphere).[6] (B) AlphaFold2 predicted structure of XenC with substrate **1** and iron modeled into the active site based on homology with the TropC crystal structure. (C) AlphaFold2 predicted structure of Anc1 with substrate **1** and iron modeled into the active site based on homology with the TropC crystal structure.

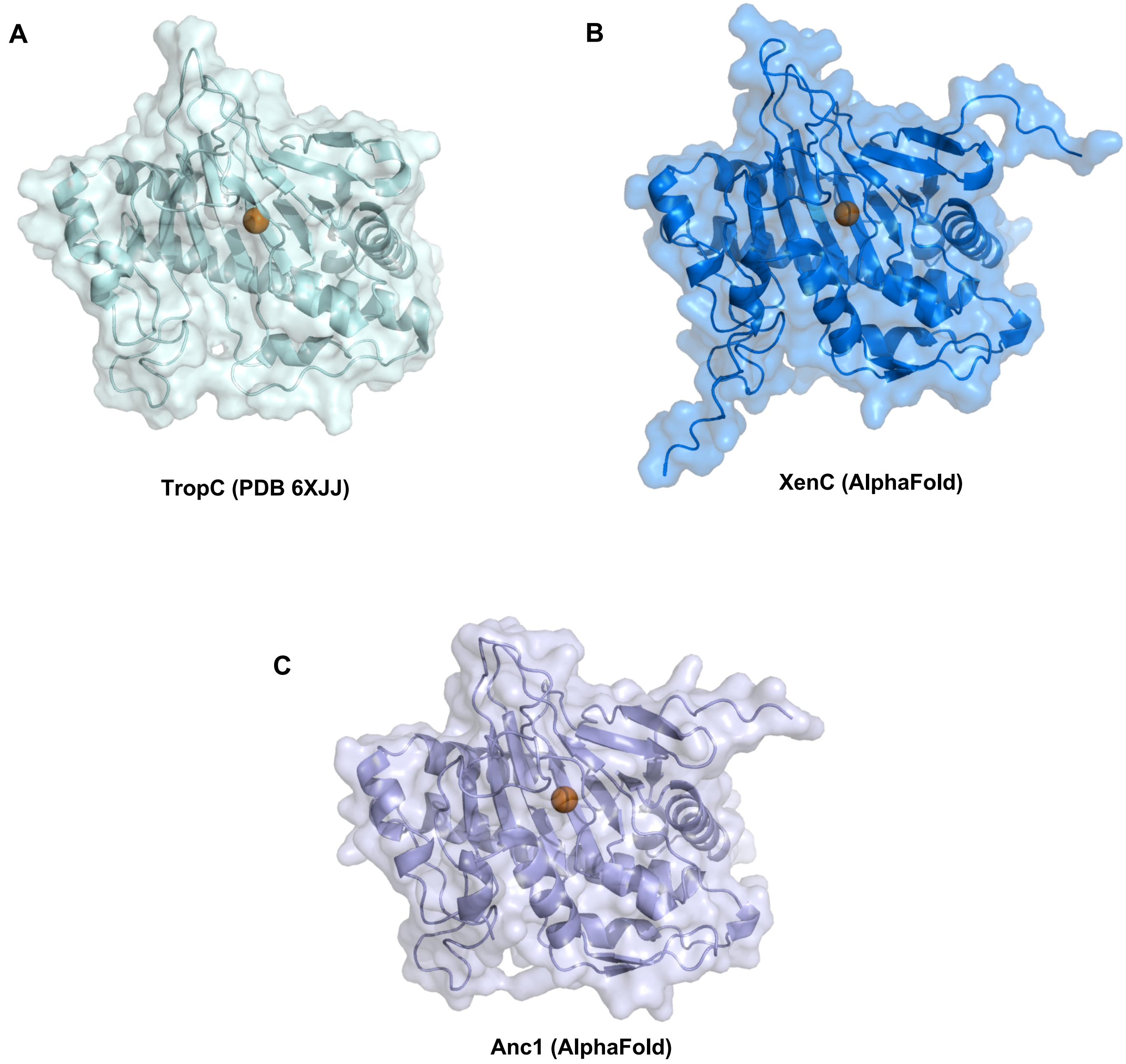

Selection of target residues. Using the AlphaFold2 predicted NHI models and homology with TropC, all residues within ~17 Å of the iron binding region in the NHI homology model active site were selected in PyMOL (command: "select bca. NHI within 17 of substrate"). Surface residues were selected in PyMOL using the command: findSurfaceResidues, doShow=1, cutoff=2.5 (Figure S6). Any residues with >95% conservation based on a MAFFT alignment with up to 5,000 other sequences were removed from the pool of selected residues to minimize selection of essential amino acids. A total of 96 residues were selected for mutagenesis for each round of SSM.

Figure S6. Residue selection on AlphaFold2 predicted structures of XenC and Anc1. (A) Active site, second coordination sphere and surface residues selected for round 1 (Rd1) of evolution of XenC. (B) Residues on second coordination sphere and surface selected for round 3 (Rd3) of evolution of XenC. (C) Active site, second coordination sphere and surface residues selected on Anc1.

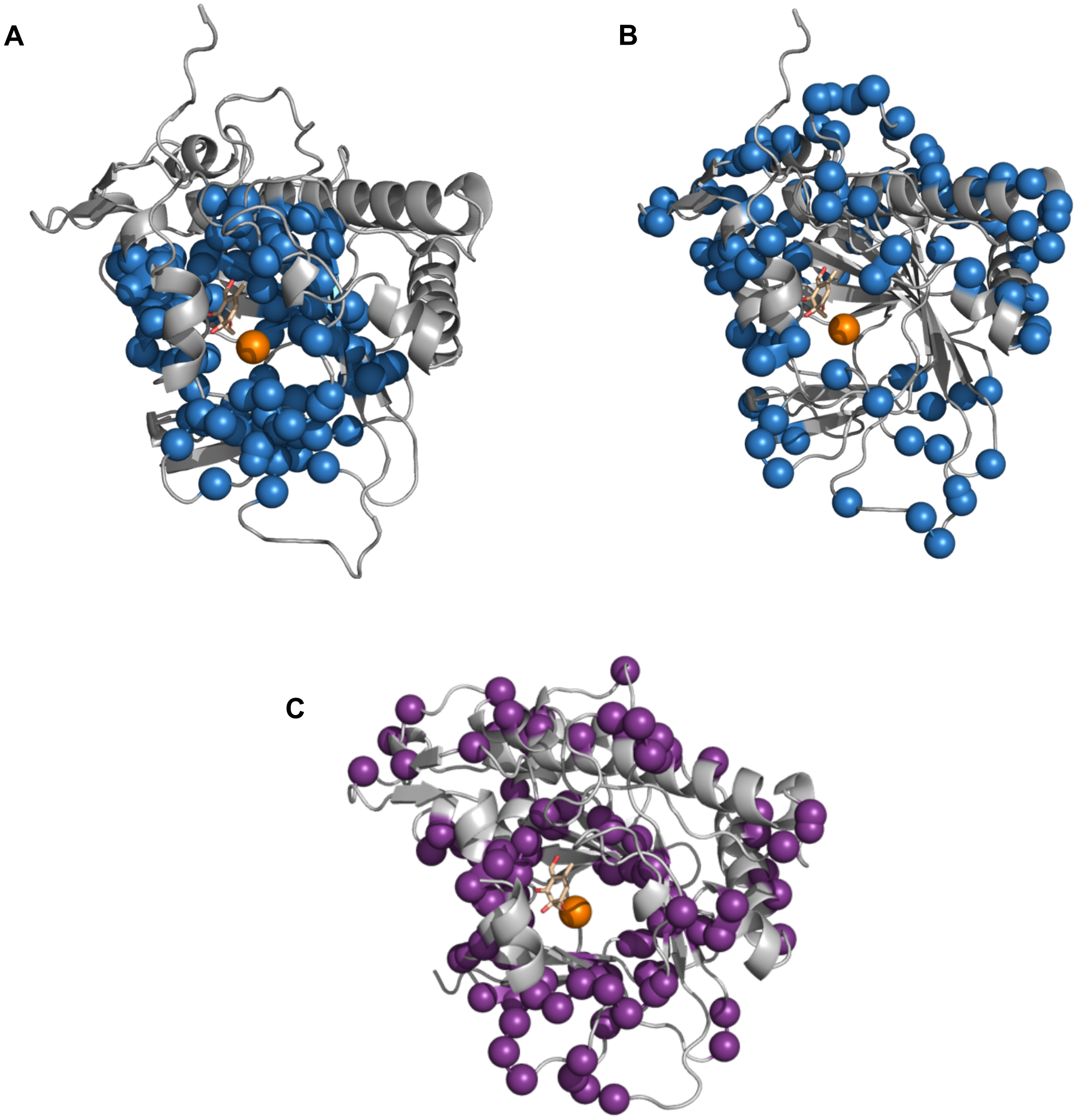

Site-saturation mutagenesis. Degenerate (NNK) forward and reverse primers were designed for each of the 96 residues being targeted in each library and purchased from IDT Technologies (Tables S2, S4, and S5). Inverse PCR reactions were set up in a 96-well PCR plate, with each 25 µL reaction containing 1X HF GC-rich Phusion buffer, 4% DMSO, 400 µM dNTPs, 0.1 ng/µL template plasmid, 0.4 units of Phusion DNA polymerase, 3 µM degenerate forward primer, and 3 µM reverse primer. The reaction conditions were programmed as follows: 98 ºC denaturation for 2 min; 25 cycles of 98 ºC for 20 s, 56 ºC for 20 s, 72 ºC for 4 min; and a final 72 ºC extension for 10 min. After amplification, 10 µL of each PCR product was pooled in a single tube. The crude PCR product mixture was purified by gel extraction from a 0.8% agarose gel using Qiagen spin columns. Residual template DNA was digested in 1X Cutsmart buffer with 2 µL DpnI (NEB) for 18 h at 37 ºC and removed with a PCR clean-up using Qiagen spin columns. The linear DNA fragments were phosphorylated in 1X T4 DNA ligase (NEB) buffer with 2 µL PNK enzyme (NEB) at 37 ºC for 60 min. Quick ligase enzyme (1 µL, NEB) was added directly to the phosphorylation reaction and incubated at room temperature for 15 min. Salts were removed from the ligation reaction with a PCR clean-up step using Qiagen spin columns. High transformation efficiency electrocompetent 10-beta *E. coli* cells (NEB) were transformed with 2 µL of the clean DNA using standard electroporation protocols. Resulting colonies were grown up and miniprepped to give a plasmid DNA library which was used to transform BL21(DE3*) E. coli* cells through a standard heat shock protocol. Resulting colonies were used directly in library screening.

Multiple site-directed mutagenesis. Each forward primer was designed to flank the desired mutation with >15 complementary nucleotides on either side of mutation and were purchased from IDT Technologies (Table S3 and Table S6). Equimolar amounts of each of the forward primers were combined and diluted down to 10 µM. PCRs were performed with the QuikChange Lightning Multi Site-Directed Mutagenesis Kit (Agilent) according to their recommended protocols. After amplification, 1 µL of the supplied DpnI was added and the digestion reaction mixture was incubated at 37 ºC for 1 h. High efficiency electrocompetent 10-beta *E. coli* cells (NEB) were transformed with 2 µL of the digested reaction using standard electroporation protocols. Resulting colonies were grown up and miniprepped to give a plasmid DNA library which was used to transform BL21(DE3*) E. coli* cells through a standard heat shock protocol. Resulting colonies were used directly in library screening.

Site-directed mutagenesis. Each forward primer was designed to flank the desired mutation with >15 complementary nucleotides on either side of mutation and were purchased from IDT Technologies (Table S6). PCRs were performed with the QuikChange Lightning Multi Site-Directed Mutagenesis Kit (Agilent) according to their recommended protocols. After amplification, 1 µL of the supplied DpnI was added and the digestion reaction was incubated at 37 ºC for 1 h. High efficiency electrocompetent 10-beta *E. coli* cells (NEB) were transformed with 2 µL of the digested reaction using standard electroporation protocols. Resulting colonies were grown up and miniprepped to give a clean plasmid DNA which was used to transform BL21(DE3*) E. coli* cells through a standard heat shock protocol.

Table S2. Primers used for site-saturation mutagenesis of XenC round 1 (Rd1). The sites targeted are listed on the "Mutation" column of the table.

Mutation	Forward Primer (5'-3')	Reverse Primer (5'-3')
I10X	NNKCCCACCGTGGATATCTCGGC	CGCGGCATTGTCGGTCAAAC
C39X	NNKCACGTATATGGTTTTTTCAATCTGGCG	GGCATTCGACATCGCATTCAC
Y42X	NNKGGTTTTTTCAATCTGGCGGG	TACGTGGCAGGCATTCGAC
F45X	NNKAATCTGGCGGGTCATGG	AAAACCATATACGTGGCAGGC
N46X	NNKCTGGCGGGTCATGGCATTC	GAAAAAACCATATACGTGGCAGGC
L47X	NNKGCGGGTCATGGCATTCCAC	ATTGAAAAAACCATATACGTGGCAGGC
F88X	NNKCGTGGTTACGAGCCGC	TGATTGTCCGATTGACTTACTAATCAAC
E92X	NNKCCGCCCGGCATCCAG	GTAACCACGGAATGATTGTCCG
P94X	NNKGGCATCCAGACACATCACAAG	CGGCTCGTAACCACGGAATG

G95X	NNKATCCAGACACATCACAAGGGC	GGGCGGCTCGTAACCAC
I96X	NNKCAGACACATCACAAGGGCCTTC	GCCGGGCGGCTCGTAAC
Q97X	NNKACACATCACAAGGGCCTTCTTC	GATGCCGGGCGGCTC
T98X	NNKCATCACAAGGGCCTTCTTCCC	CTGGATGCCGGGCGG
H99X	NNKCACAAGGGCCTTCTTCCCG	TGTCTGGATGCCGGGC
H100X	NNKAAGGGCCTTCTTCCCGATATTAAAG	ATGTGTCTGGATGCCGGG
L103X	NNKCTTCCCGATATTAAAGAGACGTTCATG	GCCCTTGTGATGTGTCTGGATG
P105X	NNKGATATTAAAGAGACGTTCATGGTCGGG	AAGAAGGCCCTTGTGATGTGTC
D106X	NNKATTAAAGAGACGTTCATGGTCGGG	GGGAAGAAGGCCCTTGTGATG
I107X	NNKAAAGAGACGTTCATGGTCGGG	ATCGGGAAGAAGGCCCTTG
K108X	NNKGAGACGTTCATGGTCGGGC	AATATCGGGAAGAAGGCCCTTG
T110X	NNKTTCATGGTCGGGCGTGAAG	CTCTTTAATATCGGGAAGAAGGCC
F111X	NNKATGGTCGGGCGTGAAGTAC	CGTCTCTTTAATATCGGGAAGAAGGC
M112X	NNKGTCGGGCGTGAAGTACCG	GAACGTCTCTTTAATATCGGGAAGAAG
V113X	NNKGGGCGTGAAGTACCGCTTG	CATGAACGTCTCTTTAATATCGGGAAG
G114X	NNKCGTGAAGTACCGCTTGACGAC	GACCATGAACGTCTCTTTAATATCGGG
S128X	NNKACGGGTCCCAACCTTTGG	GAATGTGCCACAATCAGGGTC
M154X	NNKTTAGAACTTGTAAAGAATATTCTTGCTATTTTGGCAC	GCTACCCTGGTAAGCCATGATG
V158X	NNKAAGAATATTCTTGCTATTTTGGCACAAG	AAGTTCTAACATGCTACCCTGG
S188X	NNKATTCCAATGCGTTTCTTACACTACG	CGGCTTATCCAATAAAGAGTTGAAAACG
I189X	NNKCCAATGCGTTTCTTACACTACGC	ACTCGGCTTATCCAATAAAGAGTTG
P190X	NNKATGCGTTTCTTACACTACGCCC	AATACTCGGCTTATCCAATAAAGAGTTG
M191X	NNKCGTTTCTTACACTACGCCCCC	TGGAATACTCGGCTTATCCAATAAAGAG
F193X	NNKTTACACTACGCCCCCGTC	ACGCATTGGAATACTCGGCTTATC
L194X	NNKCACTACGCCCCCGTCC	GAAACGCATTGGAATACTCGGC
H195X	NNKTACGCCCCCGTCCC	TAAGAAACGCATTGGAATACTCGG
Q208X	NNKTTTGGGGTAGCTGACCACAC	GCGTACATCTTCAAGTTGTGAGGG
F209X	NNKGGGGTAGCTGACCACACTG	CTGGCGTACATCTTCAAGTTGTG
G210X	NNKGTAGCTGACCACACTGACTTTGG	AAACTGGCGTACATCTTCAAGTTG
V211X	NNKGCTGACCACACTGACTTTGG	CCCAAACTGGCGTACATCTTC
A212X	NNKGACCACACTGACTTTGGTTGTG	TACCCCAAACTGGCGTACATC
D213X	NNKCACACTGACTTTGGTTGTGTAAGTATTTTG	AGCTACCCCAAACTGGCGTAC
T215X	NNKGACTTTGGTTGTGTAAGTATTTTGTTGCAG	GTGGTCAGCTACCCCAAACTG
F217X	NNKGGTTGTGTAAGTATTTTGTTGCAGG	GTCAGTGTGGTCAGCTACCC
G218X	NNKTGTGTAAGTATTTTGTTGCAGGAGC	AAAGTCAGTGTGGTCAGCTACC
C219X	NNKGTAAGTATTTTGTTGCAGGAGCCC	ACCAAAGTCAGTGTGGTCAGCTAC
V220X	NNKAGTATTTTGTTGCAGGAGCCC	ACAACCAAAGTCAGTGTGGTC
S221X	NNKATTTTGTTGCAGGAGCCCG	TACACAACCAAAGTCAGTGTGGTC
I222X	NNKTTGTTGCAGGAGCCCGG	ACTTACACAACCAAAGTCAGTGTG
L224X	NNKCAGGAGCCCGGAACTTCG	CAAAATACTTACACAACCAAAGTCAGTGTGG
Q225X	NNKGAGCCCGGAACTTCGGG	CAACAAAATACTTACACAACCAAAGTCAGTG
E226X	NNKCCCGGAACTTCGGGCTTAG	CTGCAACAAAATACTTACACAACCAAAGTC
S230X	NNKGGCTTAGAGGTCTACTATCCCCC	AGTTCCGGGCTCCTGC
E233X	NNKGTCTACTATCCCCCGTCGGAC	TAAGCCCGAAGTTCCGGG
V234X	NNKTACTATCCCCCGTCGGACTC	CTCTAAGCCCGAAGTTCCGG
Y235X	NNKTATCCCCCGTCGGACTCG	GACCTCTAAGCCCGAAGTTCC
Y236X	NNKCCCCCGTCGGACTCG	GTAGACCTCTAAGCCCGAAGTTC
I243X	NNKCCTGTCCCAGTTATCGACGATG	CCACGAGTCCGACGGG
P244X	NNKGTCCCAGTTATCGACGATGGATTC	AATCCACGAGTCCGACGG

V245X	NNKCCAGTTATCGACGATGGATTCG	AGGAATCCACGAGTCCGAC
P246X	NNKGTTATCGACGATGGATTCGTGATC	GACAGGAATCCACGAGTCCG
V247X	NNKATCGACGATGGATTCGTGATCAAC	TGGGACAGGAATCCACGAGTC
F252X	NNKGTGATCAACATGGGTGATATGATGC	TCCATCGTCGATAACTGGGACAG
V253X	NNKATCAACATGGGTGATATGATGCAAC	GAATCCATCGTCGATAACTGGGAC
I254X	NNKAACATGGGTGATATGATGCAACG	CACGAATCCATCGTCGATAACTG
M256X	NNKGGTGATATGATGCAACGTTACACC	GTTGATCACGAATCCATCGTCG
G257X	NNKGATATGATGCAACGTTACACCGG	CATGTTGATCACGAATCCATCGTC
D258X	NNKATGATGCAACGTTACACCGG	ACCCATGTTGATCACGAATCC
M259X	NNKATGCAACGTTACACCGGGG	ATCACCCATGTTGATCACGAATCC
M260X	NNKCAACGTTACACCGGGGGATATTAC	CATATCACCCATGTTGATCACGAATCC
Q261X	NNKCGTTACACCGGGGGATATTACC	CATCATATCACCCATGTTGATCACG
R262X	NNKTACACCGGGGGATATTACCGC	TTGCATCATATCACCCATGTTGATCAC
Y268X	NNKCGCTCAGCTCGCCATC	ATATCCCCCGGTGTAACGTTG
R269X	NNKTCAGCTCGCCATCGCG	GTAATATCCCCCGGTGTAACG
S270X	NNKGCTCGCCATCGCGTCC	GCGGTAATATCCCCCGGTG
A271X	NNKCGCCATCGCGTCCTTACAAAC	TGAGCGGTAATATCCCCCGG
R272X	NNKCATCGCGTCCTTACAAACCG	AGCTGAGCGGTAATATCCCC
V275X	NNKCTTACAAACCGCGAGAAACATCG	GCGATGGCGAGCTGAG
L276X	NNKACAAACCGCGAGAAACATCGTC	GACGCGATGGCGAGCTG
T277X	NNKAACCGCGAGAAACATCGTCAC	AAGGACGCGATGGCGAG
H284X	NNKAGTGTGGCGTTTTTTCTGAATGG	ACGATGTTTCTCGCGGTTTG
V286X	NNKGCGTTTTTTCTGAATGGGAACTTG	ACTGTGACGATGTTTCTCGC
A287X	NNKTTTTTTCTGAATGGGAACTTGGGATTAAAG	CACACTGTGACGATGTTTCTCGC
F288X	NNKTTTCTGAATGGGAACTTGGGATTAAAG	CGCCACACTGTGACGATG
L290X	NNKAATGGGAACTTGGGATTAAAGGCG	AAAAAACGCCACACTGTGACG
N291X	NNKGGGAACTTGGGATTAAAGGCG	CAGAAAAAACGCCACACTGTG
G292X	NNKAACTTGGGATTAAAGGCGAAGGC	ATTCAGAAAAAACGCCACACTGTG
W313X	NNKATTCGCGGGCGTCTGATTG	ATCTCCAACTACTGTCTCCGTTTC
I314X	NNKCGCGGGCGTCTGATTG	CCAATCTCCAACTACTGTCTCCG
R315X	NNKGGGCGTCTGATTGACACTATGG	AATCCAATCTCCAACTACTGTCTCCG
G316X	NNKCGTCTGATTGACACTATGGGACAG	GCGAATCCAATCTCCAACTACTGTC
R317X	NNKCTGATTGACACTATGGGACAGACG	CCCGCGAATCCAATCTCCAAC
L318X	NNKATTGACACTATGGGACAGACGG	ACGCCCGCGAATCCAATC
I319X	NNKGACACTATGGGACAGACGGG	CAGACGCCCGCGAATCC
D320X	NNKACTATGGGACAGACGGGGAAG	AATCAGACGCCCGCGAATC
T321X	NNKATGGGACAGACGGGGAAG	GTCAATCAGACGCCCGCG
M322X	NNKGGACAGACGGGGAAGTTATTGC	AGTGTCAATCAGACGCCCG

Table S3. Primers used for CombiSSM of XenC in round 2 (Rd2).

Mutation	Primer (5'-3')
T110X	GGGCCTTCTTCCCGATATTAAAGAGNNKTTCATGGTCGGGCGTGAA
S128X	CGACCCTGATTGTGGCACATTCNNKACGGGTCCCAACCTTTGG
V247X	CTCGTGGATTCCTGTCCCANNKATCGACGATGGATTCGTGATCAAC

Table S4. Primers used for site-saturation mutagenesis of XenC in round 3 (Rd3). The sites targeted are listed on the "Mutation" column of the table.

Mutation	Forward Primer (5'-3')	Reverse Primer (5'-3')
D20X	NNKCCTAATGCCTCGCAGGAGG	TAAGAACGCCGAGATATCCACG
P21X	NNKAATGCCTCGCAGGAGGC	ATCTAAGAACGCCGAGATATCCAC
N22X	NNKGCCTCGCAGGAGGCAC	AGGATCTAAGAACGCCGAGATATCC
A23X	NNKTCGCAGGAGGCACGTC	ATTAGGATCTAAGAACGCCGAGATATC
S24X	NNKCAGGAGGCACGTCAGGAC	GGCATTAGGATCTAAGAACGCCG
E26X	NNKGCACGTCAGGACGTTGTG	CTGCGAGGCATTAGGATCTAAGAAC
A27X	NNKCGTCAGGACGTTGTGAATGC	CTCCTGCGAGGCATTAGGATC
V31X	NNKGTGAATGCGATGTCGAATGCC	GTCCTGACGTGCCTCCTG
A34X	NNKATGTCGAATGCCTGCCACG	ATTCACAACGTCCTGACGTGC
N37X	NNKGCCTGCCACGTATATGGTTTTTTC	CGACATCGCATTCACAACGTC
V41X	NNKTATGGTTTTTTCAATCTGGCGGG	GTGGCAGGCATTCGACATC
G49X	NNKCATGGCATTCCACAAGAGACC	CGCCAGATTGAAAAAACCATATACGTG
G51X	NNKATTCCACAAGAGACCTTGCGC	ATGACCCGCCAGATTGAAAAAAC
P53X	NNKCAAGAGACCTTGCGCGAAG	AATGCCATGACCCGCCAG
E59X	NNKGCATTCGAACTTAATAAGATGTTTTTCG	GCGCAAGGTCTCTTGTGG
M66X	NNKTTTTTCGCCTTGCCAGAGGAATC	CTTATTAAGTTCGAATGCTTCGCGC
A69X	NNKTTGCCAGAGGAATCAAAAAAAGAAGTG	GAAAAACATCTTATTAAGTTCGAATGCTTC
P71X	NNKGAGGAATCAAAAAAAGAAGTGTTGATTAGTAAG	CAAGGCGAAAAACATCTTATTAAGTTCG
S74X	NNKAAAAAAGAAGTGTTGATTAGTAAGTCAATCG	TTCCTCTGGCAAGGCGAAAAAC
E77X	NNKGTGTTGATTAGTAAGTCAATCGGACAATC	TTTTTTTGATTCCTCTGGCAAGGC
S81X	NNKAAGTCAATCGGACAATCATTCCGTG	AATCAACACTTCTTTTTTTGATTCCTCTGG
K82X	NNKTCAATCGGACAATCATTCCGTGG	ACTAATCAACACTTCTTTTTTTGATTCC
I84X	NNKGGACAATCATTCCGTGGTTACGAG	TGACTTACTAATCAACACTTCTTTTTTTGATTC
G85X	NNKCAATCATTCCGTGGTTACGAGCC	GATTGACTTACTAATCAACACTTCTTTTTTTG
G95X	NNKATCCAGACACATCACAAGGGC	GGGCGGCTCGTAACCAC
Q97X	NNKACACATCACAAGGGCCTTCTTC	GATGCCGGGCGGCTC
H100X	NNKAAGGGCCTTCTTCCCGATATTAAAG	ATGTGTCTGGATGCCGGG
K101X	NNKGGCCTTCTTCCCGATATTAAAGAGTG	GTGATGTGTCTGGATGCCGG
G102X	NNKCTTCTTCCCGATATTAAAGAGTGTTTCATG	CTTGTGATGTGTCTGGATGCCG
P118X	NNKCTTGACGACCCTGATTGTGGC	TACTTCACGCCCGACCATG
D120X	NNKGACCCTGATTGTGGCACATTCTC	AAGCGGTACTTCACGCCC
D121X	NNKCCTGATTGTGGCACATTCTCAAC	GTCAAGCGGTACTTCACGCC
P122X	NNKGATTGTGGCACATTCTCAACGG	GTCGTCAAGCGGTACTTCACG

G125X	NNKACATTCTCAACGGGTCCCAAC	ACAATCAGGGTCGTCAAGCG
S136X	NNKTCTTTACCGAAAGAGAAATTTCAGGAC	GGGCCAAAGGTTGGGACC
S137X	NNKTTACCGAAAGAGAAATTTCAGGACCG	TGAGGGCCAAAGGTTGGG
P139X	NNKAAAGAGAAATTTCAGGACCGCATCATG	TAAAGATGAGGGCCAAAGGTTGG
K140X	NNKGAGAAATTTCAGGACCGCATCATG	CGGTAAAGATGAGGGCCAAAGG
K142X	NNKTTTCAGGACCGCATCATGGC	CTCTTTCGGTAAAGATGAGGGCC
D145X	NNKCGCATCATGGCTTACCAGGG	CTGAAATTTCTCTTTCGGTAAAGATGAGG
A149X	NNKTACCAGGGTAGCATGTTAGAACTTG	CATGATGCGGTCCTGAAATTTCTC
G152X	NNKAGCATGTTAGAACTTGTAAAGAATATTCTTG	CTGGTAAGCCATGATGCGGTC
S153X	NNKATGTTAGAACTTGTAAAGAATATTCTTGCTATTTTG	ACCCTGGTAAGCCATGATGC
A163X	NNKATTTTGGCACAAGGGCTGC	AAGAATATTCTTTACAAGTTCTAACATGCTAC
Q167X	NNKGGGCTGCCAAAGGAATGG	TGCCAAAATAGCAAGAATATTCTTTACAAGTTC
G168X	NNKCTGCCAAAGGAATGGGGATG	TTGTGCCAAAATAGCAAGAATATTCTTTAC
P170X	NNKAAGGAATGGGGATGCTCACC	CAGCCCTTGTGCCAAAATAGC
K171X	NNKGAATGGGGATGCTCACCGAC	TGGCAGCCCTTGTGCC
E172X	NNKTGGGGATGCTCACCGAC	CTTTGGCAGCCCTTGTGC
W173X	NNKGGATGCTCACCGACCGTTTTC	TTCCTTTGGCAGCCCTTGTG
G174X	NNKTGCTCACCGACCGTTTTCAAC	CCATTCCTTTGGCAGCCC
C175X	NNKTCACCGACCGTTTTCAACTCTTTATTG	TCCCCATTCCTTTGGCAGC
S176X	NNKCCGACCGTTTTCAACTCTTTATTGG	GCATCCCCATTCCTTTGGC
P177X	NNKACCGTTTTCAACTCTTTATTGGATAAGC	TGAGCATCCCCATTCCTTTGG
V179X	NNKTTCAACTCTTTATTGGATAAGCCGAG	GGTCGGTGAGCATCCCC
S182X	NNKTTATTGGATAAGCCGAGTATTCCAATG	GTTGAAAACGGTCGGTGAGC
D185X	NNKAAGCCGAGTATTCCAATGCGTTTC	CAATAAAGAGTTGAAAACGGTCGGTG
K186X	NNKCCGAGTATTCCAATGCGTTTCTTAC	ATCCAATAAAGAGTTGAAAACGGTCGG
S188X	NNKATTCCAATGCGTTTCTTACACTACGC	CGGCTTATCCAATAAAGAGTTGAAAAC
P200X	NNKTCACAACTTGAAGATGTACGCCAG	GACGGGGGCGTAGTGTAAG
Q202X	NNKCTTGAAGATGTACGCCAGTTTGG	TGAGGGGACGGGGG
L203X	NNKGAAGATGTACGCCAGTTTGGGG	TTGTGAGGGGACGGGG
V206X	NNKCGCCAGTTTGGGGTAGCTG	ATCTTCAAGTTGTGAGGGGACG
G218X	NNKTGTGTAAGTATTTTGTTGCAGGAGCC	AAAGTCAGTGTGGTCAGCTACC
G228X	NNKACTTCGGGCTTAGAGGTCTACTATC	GGGCTCCTGCAACAAAATACTTAC
T229X	NNKTCGGGCTTAGAGGTCTACTATCC	TCCGGGCTCCTGCAAC
S230X	NNKGGCTTAGAGGTCTACTATCCCCC	AGTTCCGGGCTCCTGC
P238X	NNKTCGGACTCGTGGATTCCTG	GGGATAGTAGACCTCTAAGCCCG
S239X	NNKGACTCGTGGATTCCTGTCCC	CGGGGGATAGTAGACCTCTAAGC
S241X	NNKTGGATTCCTGTCCCAGTTATCG	GTCCGACGGGGGATAGTAGAC
P246X	NNKGTTATCGACGATGGATTCGTGATCAAC	GACAGGAATCCACGAGTCCG
D250X	NNKGGATTCGTGATCAACATGGGTGATATG	GTCGATAACTGGGACAGGAATCC
R262X	NNKTACACCGGGGGATATTACCGC	TTGCATCATATCACCCATGTTGATCAC
G265X	NNKGGATATTACCGCTCAGCTCGC	GGTGTAACGTTGCATCATATCACCC
G266X	NNKTATTACCGCTCAGCTCGCC	CCCGGTGTAACGTTGCATCATATC
Y267X	NNKTACCGCTCAGCTCGCC	TCCCCCGGTGTAACGTTG
T277X	NNKAACCGCGAGAAACATCGTCAC	AAGGACGCGATGGCGAG
N278X	NNKCGCGAGAAACATCGTCACAGTG	TGTAAGGACGCGATGGCG
E280X	NNKAAACATCGTCACAGTGTGGCG	GCGGTTTGTAAGGACGCG
G295X	NNKTTAAAGGCGAAGGCTTTAGATGGC	CAAGTTCCCATTCAGAAAAAACGCC
L296X	NNKAAGGCGAAGGCTTTAGATGGC	TCCCAAGTTCCCATTCAGAAAAAAC
L301X	NNKGATGGCAGCGAAACGGAGAC	AGCCTTCGCCTTTAATCCCAAG

G303X	NNKAGCGAAACGGAGACAGTAGTTG	ATCTAAAGCCTTCGCCTTTAATCCC
S304X	NNKGAAACGGAGACAGTAGTTGGAG	GCCATCTAAAGCCTTCGCC
T306X	NNKGAGACAGTAGTTGGAGATTATATTCGC	TTCGCTGCCATCTAAAGCCTTC
G311X	NNKGATTATATTCGCGGGCGTCTGATTG	AACTACTGTCTCCGTTTCGCTG
Y313X	NNKATTCGCGGGCGTCTGATTG	ATCTCCAACTACTGTCTCCGTTTC
G316X	NNKCGTCTGATTGACACTATGGGACAG	GCGAATATAATCTCCAACTACTGTCTC
D320X	NNKACTATGGGACAGACGGGGAAG	AATCAGACGCCCGCGAATATAATC
T321X	NNKATGGGACAGACGGGGAAG	GTCAATCAGACGCCCGCG
M322X	NNKGGACAGACGGGGAAGTTATTGC	AGTGTCAATCAGACGCCCG
G323X	NNKCAGACGGGGAAGTTATTGCAGC	CATAGTGTCAATCAGACGCCCG
Q324X	NNKACGGGGAAGTTATTGCAGCG	TCCCATAGTGTCAATCAGACGC
T325X	NNKGGGAAGTTATTGCAGCGTGAGAG	CTGTCCCATAGTGTCAATCAGACG
Q330X	NNKCGTGAGAGTCCGAAACCGG	CAATAACTTCCCCGTCTGTCCC
R331X	NNKGAGAGTCCGAAACCGGTAGTAC	CTGCAATAACTTCCCCGTCTGTC

Table S5. Primers used for site-saturation mutagenesis of Anc1 Rd1. Primers from Rd1 were reused in subsequent rounds unless the sequence lost complementarity to the template DNA due to new mutations being carried forward. Codons that were edited to accommodate mutations added to the template DNA are highlighted in red. The sites targeted are listed on the "Mutation" column of the table.

Mutation	Library	Forward Primer (5'-3')	Library	Reverse Primer (5'-3')
N21X	Rd1-fwd	NNKGCAAGCGAAGAAGCAAAACAGCAG	Rd1-rev	CGGGCTCAGCCATGCG
	Rd3-fwd	NNKGCAAGCGAAGGGGCAAAAC		
E25X	Rd1-fwd	NNKGCAAAACAGCAGGTTGTTGAAGCAATG	Rd1-rev	TTCGCTTGCATTCGGGCTCAG
Q28X	Rd1-fwd	NNKCAGGTTGTTGAAGCAATGCGTCATG	Rd1-rev	TTTTGCTTCTTCGCTTGCATTCGG
			Rd3-rev	TTTTGCCCCTTCGCTTGC
E32X	Rd1-fwd	NNKGCAATGCGTCATGCATGTACCAC	Rd1-rev	AACAACCTGCTGTTTTGCTTCTTCG
			Rd3-rev	AACAACCTGCTGTTTTGCCCC
T39X	Rd1-fwd	NNKACCTATGGTTTCTTTTATCTGGTTGGTC	Rd1-rev	ACATGCATGACGCATTGCTTCAAC
T40X	Rd1-fwd	NNKTATGGTTTCTTTTATCTGGTTGGTCATG	Rd1-rev	GGTACATGCATGACGCATTGCTTC
Y41X	Rd1-fwd	NNKGGTTTCTTTTATCTGGTTGGTCATGG	Rd1-rev	GGTGGTACATGCATGACGCATTG
Y45X	Rd1-fwd	NNKCTGGTTGGTCATGGTGTTAGTCCG	Rd1-rev	AAAGAAACCATAGGTGGTACATGCATG
E54X	Rd1-fwd	NNKGAACAGCAGAAAGCACTGGATTGTACC	Rd1-rev	CGGACTAACACCATGACCAACCAG
K58X	Rd1-fwd	NNKGCACTGGATTGTACCAAACTGTTTTTC	Rd1-rev	CTGCTGTTCTTCCGGACTAACACC
D61X	Rd1-fwd	NNKTGTACCAAACTGTTTTTCGCACTGC	Rd1-rev	CAGTGCTTTCTGCTGTTCTTCCGG
L65X	Rd1-fwd	NNKTTTTTCGCACTGCCGATGGAAGAAC	Rd1-rev	TTTGGTACAATCCAGTGCTTTCTGCTG
A68X	Rd1-fwd	NNKCTGCCGATGGAAGAACGTATGGAAG	Rd1-rev	GAAAAACAGTTTGGTACAATCCAGTGC
M75X	Rd1-fwd	NNKGAAGTTTGGATTGGTAAAAGCATGGG	Rd1-rev	ACGTTCTTCCATCGGCAGTGC
E76X	Rd1-fwd	NNKGTTTGGATTGGTAAAAGCATGGG	Rd1-rev	CATACGTTCTTCCATCGGCAGTGC
W78X	Rd1-fwd	NNKATTGGTAAAAGCATGGGTAAAAGC	Rd1-rev	AACTTCCATACGTTCTTCCATCGGC
P93X	Rd1-fwd	NNKGGTATTCAGACCCATCATGAAGGTCTG	Rd1-rev	CGGTTCATAACCACGAAAGCTTTTACC
	Rd3-fwd	NNKGAGATTCAGACCCATCATGAAGGTC		
G94X	Rd1-fwd	NNKATTCAGACCCATCATGAAGGTCTGC	Rd1-rev	AGGCGGTTCATAACCACGAAAGC
I95X	Rd1-fwd	NNKCAGACCCATCATGAAGGTCTGCTG	Rd1-rev	ACCAGGCGGTTCATAACCACG
			Rd3-rev	CTCAGGCGGTTCATAACCACG
Q96X	Rd1-fwd	NNKACCCATCATGAAGGTCTGCTGC	Rd1-rev	AATACCAGGCGGTTCATAACCACG
			Rd3-rev	AATCTCAGGCGGTTCATAACCACG
	Rd1-fwd	NNKCATCATGAAGGTCTGCTGCCGG	Rd1-rev	CTGAATACCAGGCGGTTCATAACCAC

T97X			Rd3-rev	CTGAATCTCAGGCGGTTCATAACC
H98X	Rd1-fwd	NNKCATGAAGGTCTGCTGCCGGATAC	Rd1-rev	GGTCTGAATACCAGGCGGTTCATAAC
			Rd3-rev	GGTCTGAATCTCAGGCGGTTCATAAC
H99X	Rd1-fwd	NNKGAAGGTCTGCTGCCGGATACC	Rd1-rev	ATGGGTCTGAATACCAGGCGG
			Rd3-rev	ATGGGTCTGAATCTCAGGCGG
E100X	Rd1-fwd	NNKGGTCTGCTGCCGGATACCAAAG	Rd1-rev	ATGATGGGTCTGAATACCAGGCG
			Rd3-rev	ATGATGGGTCTGAATCTCAGGCG
G101X	Rd1-fwd	NNKCTGCTGCCGGATACCAAAGAAACC	Rd1-rev	TTCATGATGGGTCTGAATACCAGGC
			Rd3-rev	TTCATGATGGGTCTGAATCTCAGGC
L103X	Rd1-fwd	NNKCCGGATACCAAAGAAACCTTTATGG	Rd1-rev	CAGACCTTCATGATGGGTCTGAATACC
			Rd3-rev	CAGACCTTCATGATGGGTCTGAATC
K107X	Rd1-fwd	NNKGAAACCTTTATGGTTGGTGCCGAAG	Rd1-rev	GGTATCCGGCAGCAGACCTTC
T109X	Rd1-fwd	NNKTTTATGGTTGGTGCCGAAGTTCCG	Rd1-rev	TTCTTTGGTATCCGGCAGCAGAC
M111X	Rd1-fwd	NNKGTTGGTGCCGAAGTTCCGG	Rd1-rev	AAAGGTTTCTTTGGTATCCGGCAGC
E115X	Rd1-fwd	NNKGTTCCGGCAGATGATCCGGATG	Rd1-rev	GGCACCAACCATAAAGGTTTCTTTGG
P117X	Rd1-fwd	NNKGCAGATGATCCGGATGCAGGC	Rd1-rev	AACTTCGGCACCAACCATAAAGGTTTC
A118X	Rd1-fwd	NNKGATGATCCGGATGCAGGCACC	Rd1-rev	CGGAACTTCGGCACCAACCATAAAG
D119X	Rd1-fwd	NNKGATCCGGATGCAGGCACCTTTAG	Rd1-rev	TGCCGGAACTTCGGCACC
D120X	Rd1-fwd	NNKCCGGATGCAGGCACCTTTAGC	Rd1-rev	ATCTGCCGGAACTTCGGCAC
K135X	Rd1-fwd	NNKAGCCTGCCGGATGAAGAATTTCG	Rd1-rev	CGGCCACAGATTCGGACCG
P138X	Rd1-fwd	NNKGATGAAGAATTTCGTACACCGGTTATG	Rd1-rev	CAGGCTTTTCGGCCACAGATTC
E148X	Rd1-fwd	NNKTATCAGGCCAAAATGGTTGAACTGG	Rd1-rev	CATAACCGGTGTACGAAATTCTTCATC
A151X	Rd1-fwd	NNKAAAATGGTTGAACTGGTTAAAGTGC	Rd1-rev	CTGATATTCCATAACCGGTGTACG
K152X	Rd1-fwd	NNKATGGTTGAACTGGTTAAAGTGCTGCTG	Rd1-rev	GGCCTGATATTCCATAACCGGTGTAC
E155X	Rd1-fwd	NNKCTGGTTAAAGTGCTGCTGAAAATTCTG	Rd1-rev	AACCATTTTGGCCTGATATTCCATAAC
K158X	Rd1-fwd	NNKGTGCTGCTGAAAATTCTGGCACG	Rd1-rev	AACCAGTTCAACCATTTTGGCCTG
K162X	Rd1-fwd	NNKATTCTGGCACGTGGTCTGCC	Rd1-rev	CAGCAGCACTTTAACCAGTTCAACC
R166X	Rd1-fwd	NNKGGTCTGCCGAAAGAATGGAATTGTCC	Rd1-rev	TGCCAGAATTTTCAGCAGCACTTTAAC
E171X	Rd1-fwd	NNKTGGAATTGTCCGCCTGATGTTTTTG	Rd1-rev	TTTCGGCAGACCACGTGCC
N173X	Rd1-fwd	NNKTGTCCGCCTGATGTTTTTGATGAACTG	Rd1-rev	CCATTCTTTCGGCAGACCACGTG
D177X	Rd1-fwd	NNKGTTTTTGATGAACTGGCAGTTAATCCG	Rd1-rev	AGGCGGACAATTCCATTCTTTCGG
E181X	Rd1-fwd	NNKCTGGCAGTTAATCCGAGCATTCCG	Rd1-rev	ATCAAAAACATCAGGCGGACAATTCC
P189X	Rd1-fwd	NNKATGCGTCTGCTGCATTATGCAC	Rd1-rev	AATGCTCGGATTAACTGCCAGTTCATC
M190X	Rd1-fwd	NNKCGTCTGCTGCATTATGCACCG	Rd1-rev	CGGAATGCTCGGATTAACTGCCAG
R191X	Rd1-fwd	NNKCTGCTGCATTATGCACCGCAG	Rd1-rev	CATCGGAATGCTCGGATTAACTGCC
L193X	Rd1-fwd	NNKCATTATGCACCGCAGCCGG	Rd1-rev	CAGACGCATCGGAATGCTCGG
D202X	Rd1-fwd	NNKGATCGTCAGTTTGGTGTTGCAGATC	Rd1-rev	ACGAACCGGCTGCGGTG
	Rd2-fwd	NNKGATCGTCAGTTTGGTGTTGCAGG		
	Rd3-fwd	NNKGATCGTCAGATGGGTGTTGCAG		
D203X	Rd1-fwd	NNKCGTCAGTTTGGTGTTGCAGATCATACC	Rd1-rev	ATCACGAACCGGCTGCGG
	Rd2-fwd	NNKCGTCAGTTTGGTGTTGCAGGG		
	Rd3-fwd	NNKCGTCAGATGGGTGTTGCAGG		
R204X	Rd1-fwd	NNKCAGTTTGGTGTTGCAGATCATACCG	Rd1-rev	ATCATCACGAACCGGCTGCG
	Rd2-fwd	NNKCAGTTTGGTGTTGCAGGGCATAC		
	Rd3-fwd	NNKCAGATGGGTGTTGCAGGGC		
F206X	Rd1-fwd	NNKGGTGTTGCAGATCATACCGATTTTGG	Rd1-rev	CTGACGATCATCACGAACCGGC
	Rd2-fwd	NNKGGTGTTGCAGGGCATACCG		
V208X	Rd1-fwd	NNKGCAGATCATACCGATTTTGGTTGC	Rd1-rev	ACCAAACTGACGATCATCACGAACC
	Rd2-fwd	NNKGCAGGGCATACCGATTTTGG	Rd3-rev	ACCCATCTGACGATCATCACG
D210X	Rd1-fwd	NNKCATACCGATTTTGGTTGCATTACCATTC	Rd1-rev	TGCAACACCAAACTGACGATCATCAC
			Rd3-rev	TGCAACACCCATCTGACGATC
	Rd1-fwd	NNKGATTTTGGTTGCATTACCATTCTGCTG	Rd1-rev	ATGATCTGCAACACCAAACTGACGATC

T212X			Rd2-rev	ATGCCCTGCAACACCAAACTG
			Rd3-rev	ATGCCCTGCAACACCCATC
F214X	Rd1-fwd	NNKGGTTGCATTACCATTCTGCTGCAAG	Rd1-rev	ATCGGTATGATCTGCAACACCAAACTG
			Rd2-rev	ATCGGTATGCCCTGCAACAC
G215X	Rd1-fwd	NNKTGCATTACCATTCTGCTGCAAGAACC	Rd1-rev	AAAATCGGTATGATCTGCAACACC
			Rd2-rev	AAAATCGGTATGCCCTGCAACAC
I217X	Rd1-fwd	NNKACCATTCTGCTGCAAGAACCGG	Rd1-rev	GCAACCAAAATCGGTATGATCTGCAAC
			Rd2-rev	GCAACCAAAATCGGTATGCCCTG
Q222X	Rd1-fwd	NNKGAACCGGGTACAAAAGGTCTGGAAG	Rd1-rev	CAGCAGAATGGTAATGCAACCAAAATC
K227X	Rd1-fwd	NNKGGTCTGGAAGTGTGGTATCCGC	Rd1-rev	TGTACCCGGTTCTTGCAGCAG
E230X	Rd1-fwd	NNKGTGTGGTATCCGCCTACCGAAAC	Rd1-rev	CAGACCTTTTGTACCCGGTTCTTGC
W232X	Rd1-fwd	NNKTATCCGCCTACCGAAACCTGGATTC	Rd1-rev	CACTTCCAGACCTTTTGTACCCGG
Y233X	Rd1-fwd	NNKCCGCCTACCGAAACCTGGATTC	Rd1-rev	CCACACTTCCAGACCTTTTGTACCC
P235X	Rd1-fwd	NNKACCGAAACCTGGATTCCGGTTC	Rd1-rev	CGGATACCACACTTCCAGACCTTTTG
E237X	Rd1-fwd	NNKACCTGGATTCCGGTTCCGG	Rd1-rev	GGTAGGCGGATACCACACTTCC
T238X	Rd1-fwd	NNKTGGATTCCGGTTCCGGTTAAAGAAG	Rd1-rev	TTCGGTAGGCGGATACCACACTTC
P241X	Rd1-fwd	NNKGTTCCGGTTAAAGAAGGTGCATACG	Rd1-rev	AATCCAGGTTTCGGTAGGCGG
P243X	Rd1-fwd	NNKGTTAAAGAAGGTGCATACGTTATTAACATG	Rd1-rev	AACCGGAATCCAGGTTTCGGTAG
V244X	Rd1-fwd	NNKAAAGAAGGTGCATACGTTATTAACATGG	Rd1-rev	CGGAACCGGAATCCAGGTTTCG
K245X	Rd1-fwd	NNKGAAGGTGCATACGTTATTAACATGGGC	Rd1-rev	AACCGGAACCGGAATCCAGG
V250X	Rd1-fwd	NNKATTAACATGGGCGACATGATGCAG	Rd1-rev	GTATGCACCTTCTTTAACCGGAACCG
M253X	Rd1-fwd	NNKGGCGACATGATGCAGAAATGGAC	Rd1-rev	GTTAATAACGTATGCACCTTCTTTAACC
Q258X	Rd1-fwd	NNKAAATGGACCGGTGGTTATTATCGTAGC	Rd1-rev	CATCATGTCGCCCATGTTAATAACG
K259X	Rd1-fwd	NNKTGGACCGGTGGTTATTATCGTAGCG	Rd1-rev	CTGCATCATGTCGCCCATGTTAATAAC
G263X	Rd1-fwd	NNKTATTATCGTAGCGCACGTCATCGTG	Rd1-rev	ACCGGTCCATTTCTGCATCATGTC
Y264X	Rd1-fwd	NNKTATCGTAGCGCACGTCATCGTG	Rd1-rev	ACCACCGGTCCATTTCTGCATC
R269X	Rd1-fwd	NNKCATCGTGTTATTACCAGCAGCGATAAAC	Rd1-rev	TGCGCTACGATAATAACCACCGG
V272X	Rd1-fwd	NNKATTACCAGCAGCGATAAACATCGTTATAG	Rd1-rev	ACGATGACGTGCGCTACGATAATAAC
I273X	Rd1-fwd	NNKACCAGCAGCGATAAACATCGTTATAGC	Rd1-rev	AACACGATGACGTGCGCTACG
D277X	Rd1-fwd	NNKAAACATCGTTATAGCGTTGCATTTTTC	Rd1-rev	GCTGCTGGTAATAACACGATGACGTG
K278X	Rd1-fwd	NNKCATCGTTATAGCGTTGCATTTTTCCTG	Rd1-rev	ATCGCTGCTGGTAATAACACGATGAC
Y281X	Rd1-fwd	NNKAGCGTTGCATTTTTCCTGAACGG	Rd1-rev	ACGATGTTTATCGCTGCTGGTAATAAC
A284X	Rd1-fwd	NNKTTTTTCCTGAACGGTAACCTGAAACTG	Rd1-rev	AACGCTATAACGATGTTTATCGCTGC
L287X	Rd1-fwd	NNKAACGGTAACCTGAAACTGAAATGTAAAG	Rd1-rev	GAAAAATGCAACGCTATAACGATGTTTATC
N288X	Rd1-fwd	NNKGGTAACCTGAAACTGAAATGTAAAGCC	Rd1-rev	CAGGAAAAATGCAACGCTATAACGATG
L293X	Rd1-fwd	NNKAAATGTAAAGCCCTGGATGGTAGCG	Rd1-rev	TTTCAGGTTACCGTTCAGGAAAAATGC
K296X	Rd1-fwd	NNKGCCCTGGATGGTAGCGGTG	Rd1-rev	ACATTTCAGTTTCAGGTTACCGTTCAG
G300X	Rd1-fwd	NNKAGCGGTGCAGAAACCGTTG	Rd1-rev	ATCCAGGGCTTTACATTTCAGTTTCAG
I311X	Rd1-fwd	NNKCGTCAGCGTCTGATTGAAACCATGG	Rd1-rev	ATGTTCACCAACAACGGTTTCTGC
R312X	Rd1-fwd	NNKCAGCGTCTGATTGAAACCATGGGTG	Rd1-rev	AATATGTTCACCAACAACGGTTTCTGC
Q313X	Rd1-fwd	NNKCGTCTGATTGAAACCATGGGTGAAACC	Rd1-rev	ACGAATATGTTCACCAACAACGGTTTC
R314X	Rd1-fwd	NNKCTGATTGAAACCATGGGTGAAACCGG	Rd1-rev	CTGACGAATATGTTCACCAACAACGG
E317X	Rd1-fwd	NNKACCATGGGTGAAACCGGTAAAATGC	Rd1-rev	AATCAGACGCTGACGAATATGTTCACC

Table S6. Primers used for CombiSDM of Anc1 Combi-Rd2 and Combi-Rd3, and reversion of E25G mutation on Anc1Rd3.

Mutation	Library/Purpose	Primer (5'-3')
E25G	Combi-Rd2	CCCGAATGCAAGCGAAGGGGCAAAACAGCAGGTTGTTG
F206M	Combi-Rd2	GTTCGTGATGATCGTCAGATGGGTGTTGCAGGGCATAC
F206S	Combi-Rd2	GTTCGTGATGATCGTCAGAGTGGTGTTGCAGGGCATAC
G94E	Combi-Rd2	CGTGGTTATGAACCGCCTGAGATTCAGACCCATCATGAAG
Q28G	Combi-Rd2	GCAAGCGAAGAAGCAAAAGGGCAGGTTGTTGAAGCAATGC
E230T	Combi-Rd2	GAACCGGGTACAAAAGGTCTGACGGTGTGGTATCCGCCTAC
F206K	Combi-Rd2	CGGTTCGTGATGATCGTCAGAAGGGTGTTGCAGGGCATACCG
R204E	Combi-Rd2	CCGCAGCCGGTTCGTGATGATGAGCAGTTTGGTGTTGCAG
N21R	Combi-Rd3	GCATGGCTGAGCCCGAGGGCAAGCGAAGG
Q28G	Combi-Rd3	CAAGCGAAGGGGCAAAAGGGCAGGTTGTTGAAGCAATG
G94F	Combi-Rd3	GTAGCTTTCGTGGTTATGAACCGCCTTTTATTCAGACCCATCATGAAG
P138A	Combi-Rd3	GCCGAAAAGCCTGGCGGATGAAGAATTTCGTAC
R166G	Combi-Rd3	CTGAAAATTCTGGCAGGTGGTCTGCCGAAAGAATG
R204E	Combi-Rd3	GCAGCCGGTTCGTGATGATGAGCAGATGGGTGTTGCAG
E230T	Combi-Rd3	GAACCGGGTACAAAAGGTCTGACGGTGTGGTATCCGCCTAC
G25E	E25G reversion	CCCGAATGCAAGCGAAGAAGCAAAACAGCAGGTTGTTG

Protein library production in 96-well culture plates. Chemically competent BL21(DE3) cells were transformed with the DNA library generated from PCR containing the mutated *nhi* genes, the template gene for each round, and a pET28a empty vector using standard heat shock procedures. The transformations were used to directly inoculate 500 µL LB containing 50 µg/mL kanamycin in a 96-deep well plate and grown overnight at 37 ºC, 350 rpm. In each plate, eight biological replicates of the template for each round of evolution were included as positive controls and two replicates of the empty vector as a negative control. Additionally, two wells were not inoculated to serve as a cross-contamination control. Overnight cultures (25 µL each) were used to inoculate 475 µL of TB containing 4% glycerol (v/v) and 50 µg/mL kanamycin. Also, 50 µL of each overnight culture was combined with 50 µL of 50% glycerol to prepare glycerol stocks for future use which were immediately stored at –80 ºC. The cultures were incubated at 37 ºC, 350 rpm until reaching an optical density at 600 nm of ~0.6–0.8. IPTG was added to 0.5 mM to induce expression. Cultures were incubated at 20 ºC, 350 rpm for 20 h and harvested by centrifugation at 1,000 x g at 4 ºC for 15 min. Plates containing harvested cells were used fresh or stored at –80 ºC before lysing for reactions.

Protein production for validation experiments in 96-well culture plates. To validate primary library screen hits, sterile toothpicks were used to scratch glycerol stocks of the wells containing the hits from the primary screen to inoculate three individual cultures (3 biological replicates) of 500 µL LB containing 50 µg/mL kanamycin in a 96-well plate. In each plate, eight biological replicates of the template for each round of evolution were included as positive controls and two replicates of the empty vector as a negative control. Additionally, two wells were not inoculated to serve as a cross-contamination control. The inoculated cultures were grown overnight at 37 ºC, 350 rpm. Overnight cultures (25 µL each) were used to inoculate 475 µL of TB containing 4% glycerol (v/v) and 50 µg/mL kanamycin. The cultures were incubated at 37 ºC, 350 rpm until reaching an optical density at 600 nm (OD600) of ~0.6–0.8. IPTG was added to 0.5 mM to induce expression. Cultures were incubated at 20 ºC, 350 rpm for 20 h and harvested by centrifugation at 1,000 x g at 4 ºC for 15 min. Plates containing harvested cells were used fresh or stored at –80 ºC before lysing for reactions.

IV. Biocatalytic Reactions

Stock solutions. Stock solutions of each substrate (100 mM) were prepared by dissolving the substrate in DMSO (analytical grade). Stock solutions of NADP+ (100 mM) and sodium phosphite (Na_2HPO_3, 500 mM) were prepared and stored at –20 ºC until further use. Phosphite dehydrogenase (PTDH) was expressed and purified according to previously reported protocols.[7] Aliquots of PTDH (500 µM) were stored at –80 ºC until needed. Stock solutions of α-ketoglutaric acid (α-KG, 100 mM) were freshly prepared before each reaction, stored on ice, and used within 3 h.

Figure S7. General reaction scheme of telescoped enzymatic sequence to produce tropolones.

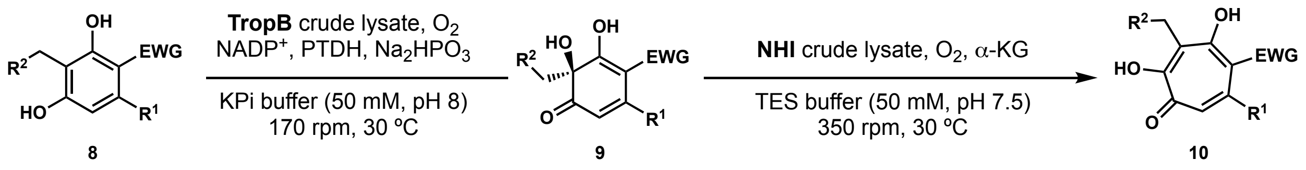

Oxidative dearomatization with TropB for library experiments. Each reaction contained 50 mM KPi buffer pH 8.0 (6 mL of a 1 M solution), 1.7 g/L substrate (12 mL, 100 mM), 3 mM NADP+ (3.6 mL, 100 mM), 20 mM sodium phosphite (4.8 mL, 500 mM), 3 µM PTDH (720 µL, 500 µM), 40 % v/v TropB lysate (48 mL) and Milli-Q water to a final volume of 120 mL. Reactions were carried out at 30 ºC and 170 rpm shaking for 4 h. Precipitated biomolecules were pelleted by centrifugation (4,000 x g, 10 min) before adding to the ring expansion reaction. Reaction progress was monitored by UPLC-DAD using a Phenomenex Kinetex (1.7 µm C18, 2.1 x 30 mm) column under the following conditions: mobile phase (Solvent A: Milli-Q water + 0.1% formic acid; Solvent B: acetonitrile + 0.1% formic acid) 5% solvent B for 0.5 min, 5% to 100% solvent B over 1.5 min, 100% solvent B for 0.5 min, 100% to 5% solvent B over 0.5 min; flow rate: 0.75 mL/min.

NHI lysate reactions for library screening and validations. *Protocol A:* 96-well plates containing freshly harvested cells were lysed following standard freeze-thaw protocols. 100 µL of reaction mix (1.2 g/L dearomatized substrate from TropB reaction and 14 mM α-KG in 50 mM TES pH 7.5) were added to each well. Reactions were incubated at 30 ºC for 1 h and then quenched with 100 µL of 1 M HCl. After 10 min, 900 µL methanol containing 100 µM 2,6-dihydroxyacetophenone (internal standard) were added to dilute reactions and the plates were centrifuged at 1,000 x g for 30 min at 4 ºC before taking aliquots for analysis plates. *Protocol B:* 96-well plates containing freshly harvested cells were resuspended in 250 µL of plate lysis buffer (50 mM TES pH 7.5, 2 mg/mL lysozyme, 0.5 mg/mL polymyxin B sulfate, 100 µM PMSF, and 1 U/mL DNaseI). The lysed plates were then heat treated at 40–50 ºC for 1 h shaking at 350 rpm before centrifuging at 1,000 x g for 30 min at 4 ºC. The clarified lysate (0.5–10 µL) was transferred to a new plate containing the reaction mix (1.2 g/L dearomatized substrate from TropB reaction and 14 mM α-KG) to a final volume of 100 µL in 50 mM TES pH 7.5. Reactions were incubated at 30 ºC for 1 h and then quenched with 100 µL of 1 M HCl. After 10 min, 900 µL methanol containing 100 µM 2,6-dihydroxyacetophenone (internal standard) were added to dilute reactions and the plates were centrifuged at 1,000 x g for 30 min at 4 ºC before taking aliquots for analysis plates.

Calculating Relative Percent Conversion. Relative conversion to stipitaldehyde was determined by analysis of each reaction after 1 h. RF-MS analysis was performed on an Agilent Rapid Fire 400 coupled to a TOF Mass Spectrometer. Relative conversion to tropolone product was calculated as:

$$\text{eq. 1} \qquad \frac{\frac{\text{PeakArea}_{ring\ expansion\ product}}{\text{PeakArea}_{internal\ standard}}}{\frac{\text{PeakArea}_{ring\ expansion\ product} + \text{PeakArea}_{dearomatized\ substrate}}{\text{PeakArea}_{internal\ standard}}}.$$

In vitro reactions were performed and analyzed in biological triplicates with reported conversions as an average of those trials.

High throughput RapidFire-MS method. Reactions were analyzed for enhanced tropolone formation in an optimized high-throughput screen using an Agilent RapidFire 400 High throughput Mass Spectrometry System linked to an Agilent 6545 TOF-MS. Samples for analysis were prepared by diluting 5 µL of quenched reactions in 500 µL of 50% MeOH followed by a 1:10 dilution with 50% MeOH in shallow 96-well plates (analysis plate, Axygen) unless otherwise noted. The analysis plates were heat-sealed with pierceable aluminum covers (Agilent). Each RapidFire injection was programmed as follows: 0.6 s to aspirate the sample, 3 s to load the sample onto the C4 solid phase extraction (SPE) cartridge with water buffered with 5 mM ammonium formate pH 8 and 0.1% v/v acetic acid (1.5 mL/min), 5 s to elute the sample from the cartridge using 80% ethyl acetate, 20% isopropanol buffered with 5 mM ammonium formate pH 8 and 0.1% v/v acetic acid (0.8 mL/min), and 0.5 s to reequilibrate. One to two blank injections were included after each sample injection to reduce carry-over. The SPE cartridges were replaced after 2500 injections.

The peak areas for the extracted ion chromatograms for internal standard, substrate, and tropolone product were collected for each sample using the MassHunter Qualitative Analysis 10.0 software. The relative percent conversions to tropolone products were calculated. For library screens, the average percent conversion resulting from the template reactions for each plate (n=8) was set to 1.0 to compare with all variants. The conversions for the variant reactions were normalized to the template reactions, giving fold improvement scores for each variant.

Figure S8. Relative activity of XenC towards tropolone formation at 30 ºC compared to 25 ºC. Reactions were performed in triplicates using crude lysate of XenC (10% v/v), 1.2 g/L substrate **1**, and 14 mM α-KG in 50 mM TES buffer, pH 7.5 in a final volume of 100 µL. Reactions were quenched by addition of 100 µL of methanol containing 0.5 mM benzophenone as internal standard. by UPLC-DAD using a Phenomenex Kinetex (1.7 µm C18, 2.1 x 30 mm) column under the following conditions: mobile phase (Solvent A: Milli-Q water + 0.1% formic acid; Solvent B: acetonitrile + 0.1% formic acid) 5% solvent B for 0.5 min, 5% to 100% solvent B over 1.5 min, 100% solvent B for 0.5 min, 100% to 5% solvent B over 0.5 min; flow rate: 0.75 mL/min. The ratio of stipitaldehyde and internal standard at 30 ºC was normalized by the ratio of stipitaldehyde and internal standard at 25 ºC to determine relative activity

	25 ºC	30 ºC
**Response**	0.82 ± 0.04	0.75 ± 0.06
**Relative Activity (%)**	100 ± 4	91.70 ± 7

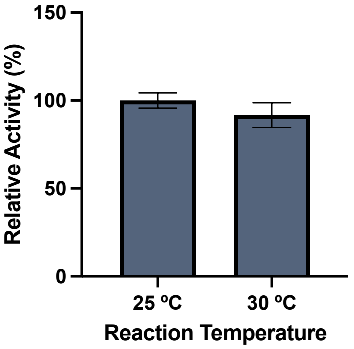

Figure S9. Stipitaldehyde calibration curve to determine sensitivity of RF-MS Method.

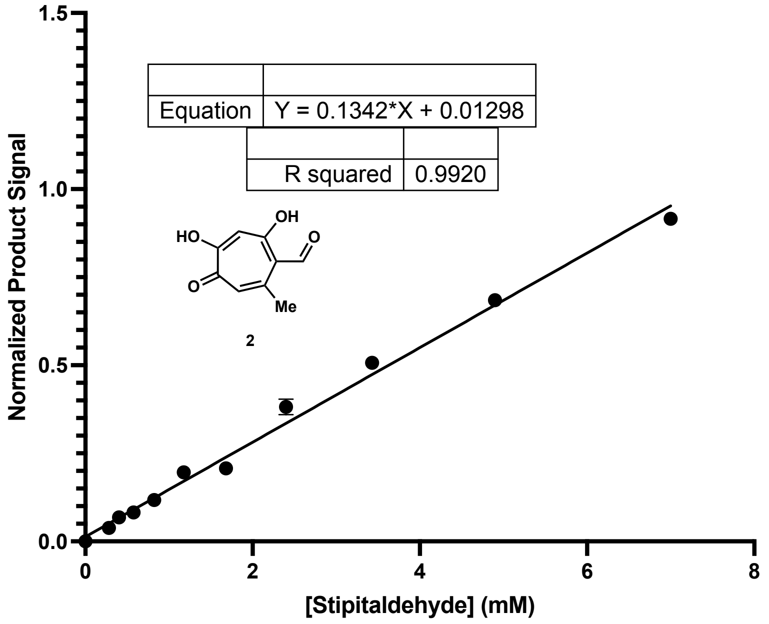

Figure S10. Representative LC-TOF extracted ion chromatogram (EIC) of a stipitaldehyde authentic standard (m/z = 179.0350) that demonstrates tropolone carryover. Chromatography conditions: Phenomenex Kinetex (1.7 µm C18, 2.1 x 30 mm) column; Solvent A: Milli-Q water + 0.1% formic acid; Solvent B: acetonitrile + 0.1% formic acid; 5% solvent B for 0.5 min, 5% to 100% solvent B over 1.5 min, 100% solvent B for 0.5 min, 100% to 5% solvent B over 0.5 min; flow rate: 0.75 mL/min.

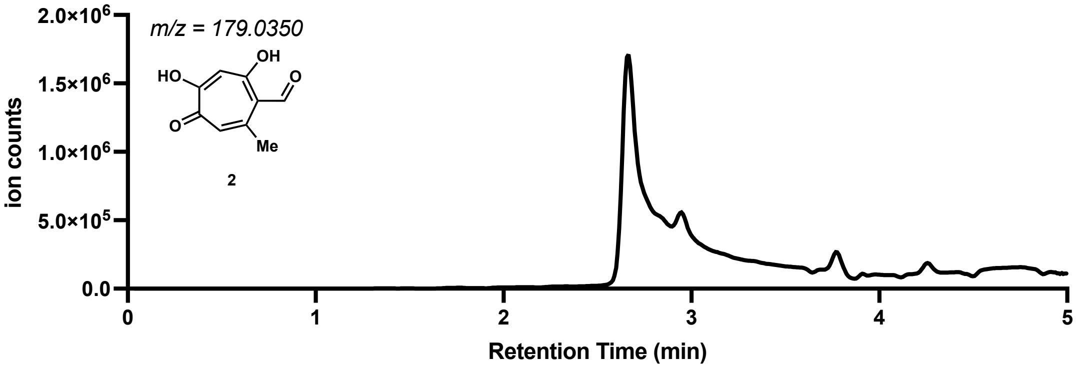

Figure S11. Waterfall plots of primary screens of each successful round of directed evolution. Template distribution (gray) shown first followed by library distribution (blue).

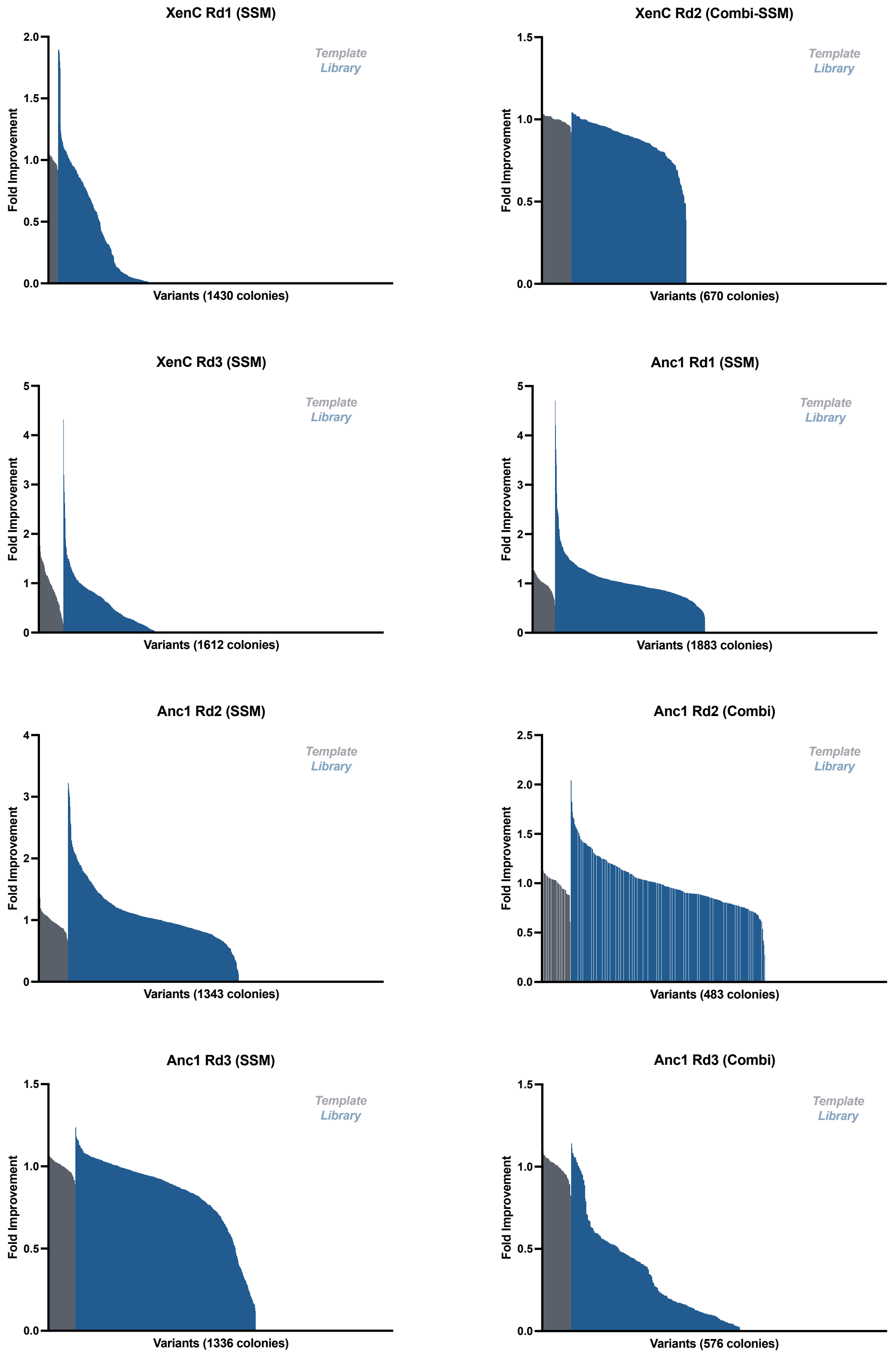

Table S7. Screening details and percentage of total active variants for each round of evolution.

Template	Colonies Screened	Library	Screening Protocol	Evolution Pressure	Active Variants
XenC	1430	SSM_Rd1	Protocol A	High Substrate	28%
XenCRd1	670	CombiSSM_Rd2	Protocol A	High Substrate	36%
XenCRd2	1612	SSM_Rd3	Protocol B	High Substrate, High Temperature, Low Lysate	29%
Anc1	1883	SSM_Rd1	Protocol B	High Substrate, High Temperature, Low Lysate	46%
Anc1Rd1	1343	SSM_Rd2	Protocol B	High Substrate, High Temperature, Low Lysate	54%
Anc1Rd1	483	Combi_Rd2	Protocol B	High Substrate, High Temperature, Low Lysate	61%
Anc1Rd2	1338	SSM_Rd3	Protocol B	High Substrate, High Temperature, Low Lysate	57%
Anc1Rd2	576	Combi_Rd3	Protocol B	High Substrate, High Temperature, Low Lysate	53%

In vitro analytical-scale NHI lysate reactions with variants. 96-well plates containing freshly harvested cells were resuspended in 250 µL of plate lysis buffer (50 mM TES pH 7.5, 2 mg/mL lysozyme, 0.5 mg/mL polymyxin B sulfate, 100 µM PMSF, and 1 U/mL DNaseI). The cells were lysed by incubation at 25 ºC, 350 rpm for 2 h. The lysis plates were then centrifuged at 1,000 x g for 30 min at 4 ºC. The clarified lysate (50 µL) was heat treated at 37 ºC for 15 min using a thermocycler before transferring 5 µL of heat-treated lysate to a new plate with each well containing 95 µL reaction mix (final concentration of 1.2 g/L dearomatized substrate from TropB reaction and 14 mM α-KG) in 50 mM TES pH 7.5. Reactions were incubated at 30 ºC for 1 h and then quenched with 100 µL of 1 M HCl. After 10 min, 900 µL methanol containing 100 µM 2,6-dihydroxyacetophenone (internal standard) were added to dilute reactions and the plates were centrifuged at 1,000 x g for 30 min at 4 ºC before aliquots for analysis were taken.

Table S8. Averaged conversion to product with final variants and respective fold improvement over wt-XenC.

Enzyme	Average Relative Conversion (%)	Standard Deviation (%)	Fold Improvement
**wt-XenC**	7.39	1.20	1.00
**XenCRd1**	10.23	0.76	1.38
**XenCRd2**	12.73	1.25	1.72
**XenCRd3**	13.43	0.81	1.82
**Anc1**	7.42	1.22	1.00
**Anc1Rd1**	15.37	1.84	2.07
**Anc1Rd2**	42.08	1.52	5.69
**Anc1Rd3**	44.55	1.49	6.03
**pET28a**	n.d.	n/a	0

Figure S12. Extracted ion chromatograms (EIC) for reactions (triplicate) with XenC variants and pET28 empty vector control with substrate **1**. Each peak corresponds to each sample injection. Product mass peak area is reported over each peak. Traces scaled to the largest peak.

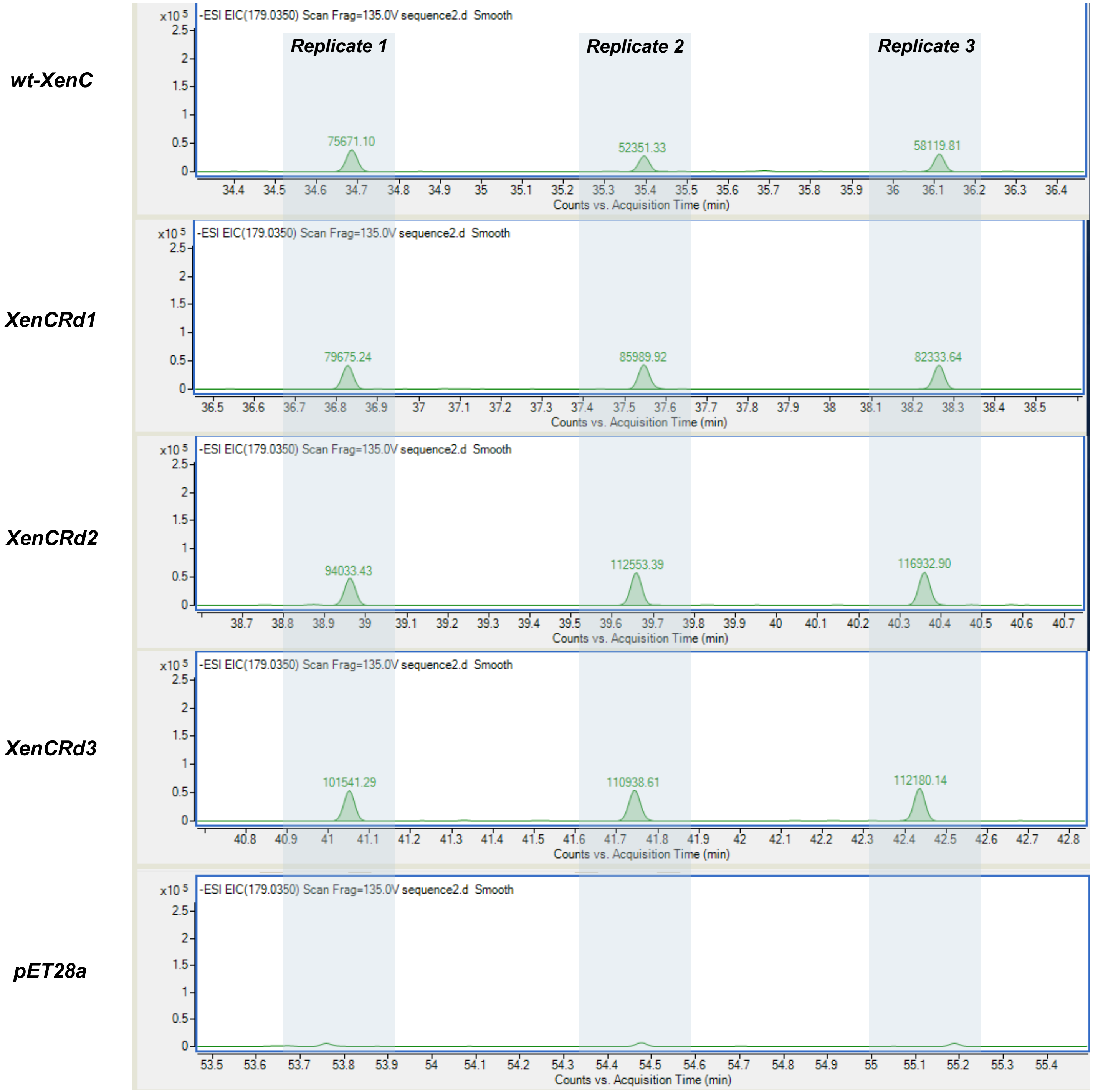

Figure S13. Extracted ion chromatograms (EIC) for reactions (triplicate) with Anc1 variants and pET28 empty vector control with substrate **1**. Each peak corresponds to each sample injection. Product mass peak area is reported over each peak. Traces scaled to the largest peak

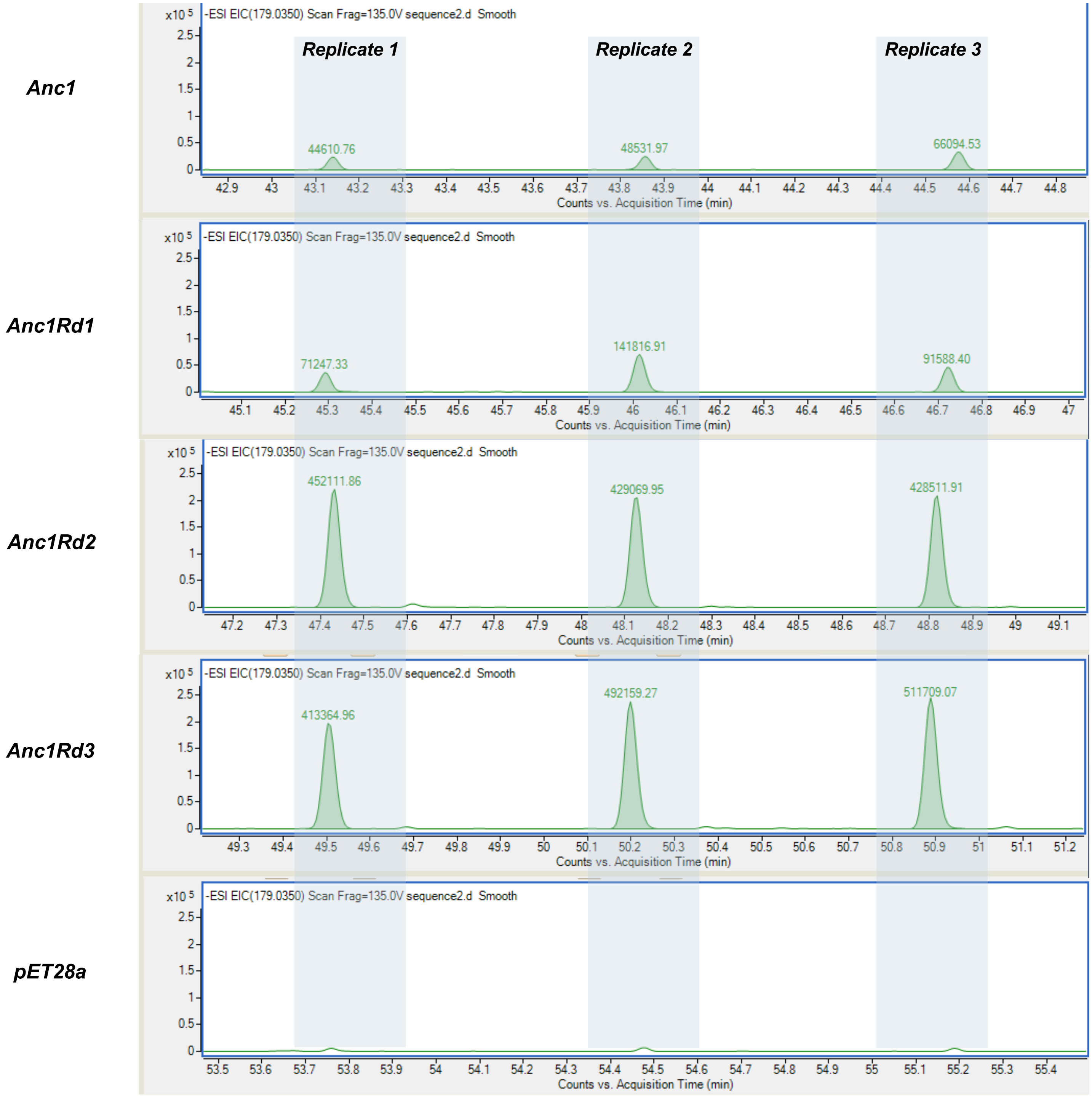

Figure S14. Relative reactivity of variant with E25G reversion (Anc1Rd3_E25G rev) as compared to Anc1Rd3. Spectra are scaled to the highest peak area. Each major peak represents a sample injection followed by two wash injections for three biological replicates (R1–3).

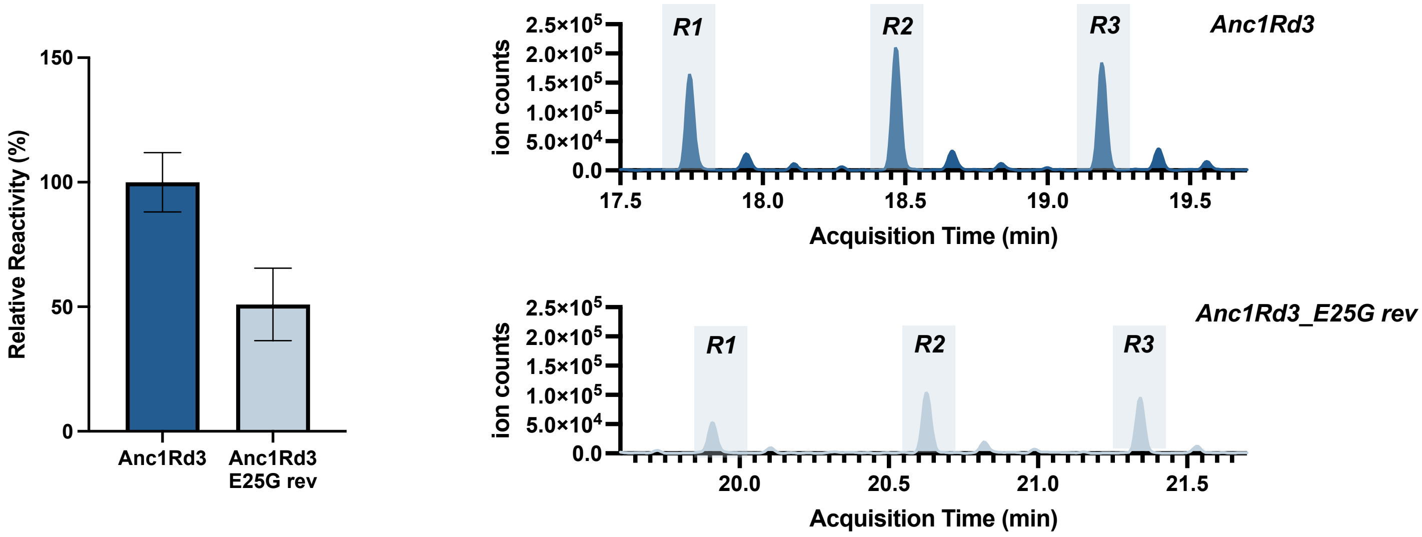

Time course reaction. Each reaction contained 50 mM TES buffer pH 7.5 14 mM α-KG, 4 mM sodium ascorbate, 0.2 mM $FeSO_4 \cdot 7H_2O$, 1.2 g/L substrate **1**, 2 µM purified NHI enzyme and Milli-Q water to a final volume of 100 µL. Reaction duplicates were incubated at 30 ºC with 110 rpm shaking for a total of 3 h, quenching at 0, 5, 10, 20, 30, 60, 120, and 180 by addition of 100 µL of 1 M HCl. After 10 min, 900 µL methanol containing 50 µM 2,6-dihydroxyacetophenone (internal standard) were added to dilute reactions and the plates were centrifuged at 1,000 x g for 30 min at 4 ºC. Aliquots (10 µL) were taken from each quenched reaction well and were diluted in 490 µL of 50% v/v methanol in Milli-Q water. Then, 25 µL of diluted samples were taken and diluted further in 225 µL of methanol in Milli-Q water for analysis using the optimized RapidFire-MS method. The relative conversion was plotted against time to produce the time course curves.

Figure S15. Time course reactions with XenC and Anc1Rd3. A no enzyme control (NEC) was used as a negative control.

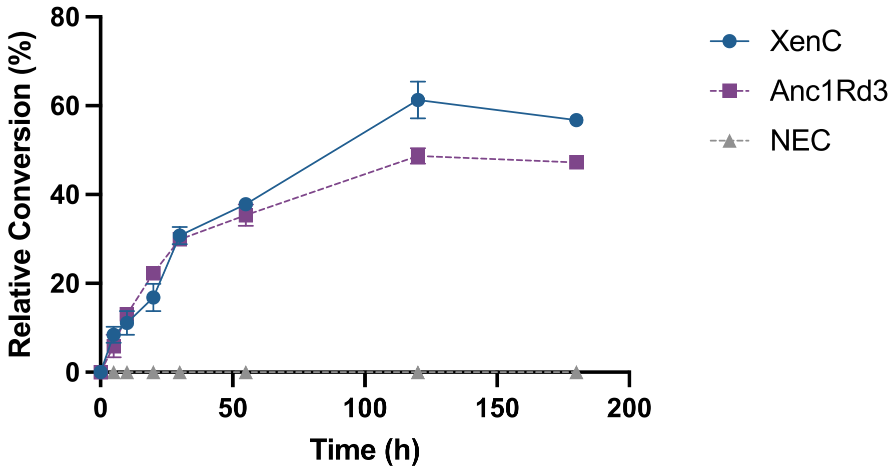

Initial Rate Determination. A master mix containing final concentrations of 100 mM TES buffer pH 7.5, 28 mM α-KG, 8 mM sodium ascorbate, 0.4 mM $FeSO_4 \cdot 7H_2O$, and Milli-Q water to a final volume of 2988 μL was prepared for each unique enzyme reaction. To each individual master mix, 12 μL of a 25 μM stock of purified XenC or Anc1Rd3 was added to reach a final volume of 3 mL. To determine the initial rate of Anc1, a 12.5 μM pure enzyme stock was used. A no enzyme control master mix was prepared by replacing the pure enzyme by storage buffer. Immediately after adding the enzyme, 500 μL of each master mix was combined with 500 μL of 500 μM substrate **1** at room temperature to initiate the reactions. The final concentrations of all reaction components were as follows: 50 mM TES pH 7.5, 250 μM substrate **1**, 14 mM α-KG, 4 mM sodium ascorbate, 0.2 mM $FeSO_4 \cdot 7H_2O$ and 50 nM XenC and Anc1Rd3, or 25 nM Anc1. Multiple time points were taken within 1 minute of reaction initiation from duplicate reactions by taking 10 μL aliquots from each reaction and quenching in 90 μL of methanol that contained 0.08 μM 2,6-dihydroxyacetophenone and 2 mM HCl. For the t = 0 s time point, 5 μL of the enzyme-containing master mix was added directly into the quenching solution which contained 5 μL of the 500 μM substrate stock. The quenched reactions were directly analyzed using the optimized RapidFire-MS method and a calibration curve of product was used to determine product concentrations.

Figure S16. Calibration curve used to determine product concentration in initial rate experiment. Normalized product signal corresponds to the peak area of the product peak divided by internal standard peak area.

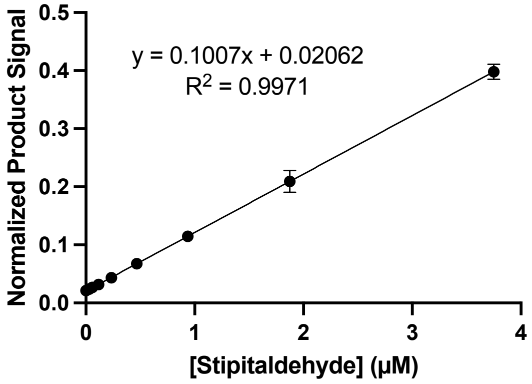

Figure S17. Progress curves used to determine the initial rates of XenC, Anc1 and Anc1Rd3 and summary of initial rate data.

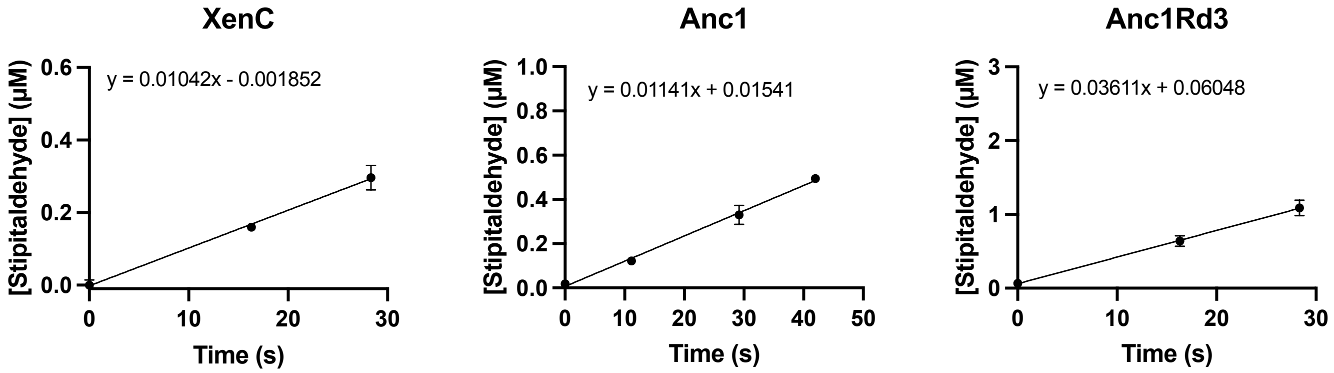

Enzyme	Slope (μM/s)	[Enzyme] (μM)	Initial Rate (s^{-1})
**XenC**	0.0104 ± 0.0008	0.05	0.21 ± 0.02
**Anc1**	0.0114 ± 0.0008	0.025	0.46 ± 0.03
**Anc1Rd3**	0.036 ± 0.002	0.05	0.72 ± 0.05

Analytical-scale Heat Challenge Experiments. 96-well plates containing freshly harvested cells were resuspended in 250 µL of plate lysis buffer (50 mM TES pH 7.5, 2 mg/mL lysozyme, 0.5 mg/mL polymyxin B sulfate, 100 µM PMSF, and 1 U/mL DNaseI). The cells were lysed by incubation at 25 ºC, 350 rpm for 2 h. The lysis plates were then centrifuged at 1,000 x g for 30 min at 4 ºC. The clarified lysate (50 µL) was heat treated at 50 ºC for 15 min using a thermocycler before transferring 2 µL of heat-treated lysate to a new plate with each well containing 98 µL reaction mix (final concentration of 1.2 g/L dearomatized substrate from TropB reaction and 14 mM α-KG) in 50 mM TES pH 7.5. Reactions were incubated at 30 ºC for 2 h and then quenched with 100 µL of 1 M HCl. After 10 min, 900 µL methanol containing 50 µM 2,6-dihydroxyacetophenone (internal standard) were added to dilute reactions and the plates were centrifuged at 1,000 x g for 30 min at 4 ºC. Aliquots (10 µL) were taken from each quenched reaction well and were diluted in 490 µL of 50% v/v methanol in Milli-Q water. Then, 25 µL of diluted samples were taken and diluted further in 225 µL of methanol in Milli-Q water for analysis using the optimized RapidFire-MS method. The product concentration was determined using a fresh calibration curve of stipitaldehyde.

Figure S18. Calibration curve used to determine product concentration in heat challenge experiments. Normalized product signal corresponds to the peak area of the product peak divided by internal standard peak area.

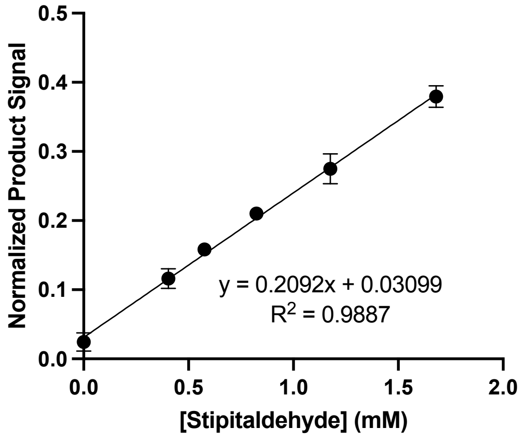

Table S9. Average calculated concentrations of stipitaldehyde for reactions performed with XenC and Anc1Rd3 after exposure to different preincubation temperatures. For wells where no product signal was detected, the notation “n.d.” has been used.

Enzyme	25 ºC	50 ºC
XenC	0.48 ± 0.06 mM	n.d.
Anc1Rd3	0.75 ± 0.06 mM	0.71 ± 0.02 mM

Analytical-scale oxidative dearomatization of substrate panel with TropB. Each reaction contained 50 mM KPi buffer pH 8.0 (250 µL of a 1 M solution), 0.8–1.4 g/L substrate (250 µL, 100 mM), 1 mM NADP+ (50 µL, 100 mM), 10 mM sodium phosphite (100 µL, 500 mM), 1 µM PTDH (10 µL, 500 µM), 20% v/v TropB (1 mL) and Milli-Q water to a final volume of 5 mL. Reactions were carried out at 30 ºC and 170 rpm shaking for 4 h in a 24-deep well plate. Precipitated biomolecules were pelleted by centrifugation (1,000 x g, 10 min) before adding to the ring expansion reaction master mix. Reaction progress was monitored by UPLC-DAD using a Phenomenex Kinetex (1.7 µm C18, 2.1 x 30 mm) column under the following conditions: mobile phase (Solvent A: Milli-Q water + 0.1% formic acid; Solvent B: acetonitrile + 0.1% formic acid) 5% solvent B for 0.5 min, 5% to 100% solvent B over 1.5 min, 100% solvent B for 0.5 min, 100% to 5% solvent B over 0.5 min; flow rate: 0.75 mL/min.

Analytical-scale NHI lysate reactions with substrate panel. 96-well plates containing freshly harvested cells were resuspended in 250 µL of plate lysis buffer (50 mM TES pH 7.5, 2 mg/mL lysozyme, 0.5 mg/mL polymyxin B sulfate, 100 µM PMSF, and 1 U/mL DNaseI). The cells were lysed by incubation at 25 ºC, 350 rpm for 2 h. The lysis plates were then centrifuged at 1,000 x g for 30 min at 4 ºC. The clarified lysate (50 µL) was heat treated at 37 ºC for 15 minutes using a thermocycler before transferring 50 µL to a new plate with each well containing 50 µL reaction mix, reaching a final concentration of 0.3–0.6 g/L dearomatized substrate from TropB reaction and 4 mM α-KG in 50 mM TES pH 7.5. Reactions were incubated at 37 ºC for 1 h and then quenched with 100 µL of 1 M HCl. After 10 min, 200 µL methanol containing 1 µM 2,6-dihydroxyacetophenone (internal standard) was added and plates were centrifuged at 1,000 x g for 30 min at 4 ºC to prepare analysis plates. The peak area of tropolone product was normalized by the peak area of internal standard.

Figure S19. Dearomatized compounds used for substrate scope investigation.

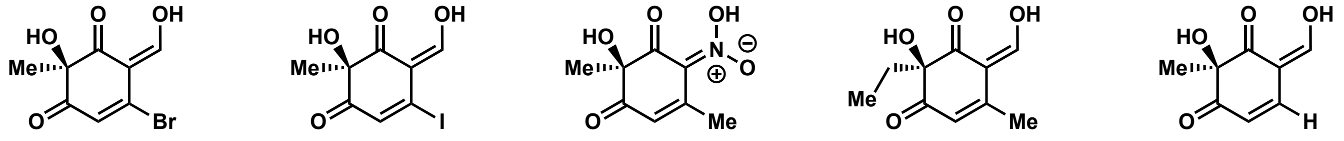

3 4 5 6 S6

Table S10. Normalized average product signals of unnatural substrates for ring expansion with wt-XenC and XenC variants. For wells where no product signal was detected, the notation "n.d." has been used.

Substrate	wt-XenC	XenCRd1	XenCRd2	XenCRd3	Empty Vector
**2**	0.015 ± 0.002	0.0200 ± 0.0006	0.015 ± 0.001	0.017 ± 0.001	n.d.
**3**	0.041 ± 0.002	0.048 ± 0.004	0.035 ± 0.002	0.0402 ± 0.0003	n.d.
**4**	0.09 ± 0.01	0.12 ± 0.01	0.107 ± 0.008	0.110 ± 0.006	n.d.
**5**	0.031 ± 0.002	0.048 ± 0.002	0.031 ± 0.002	0.0273 ± 0.0002	n.d.
**S6**	0.0073 ± 0.0003	0.011 ± 0.001	0.0055 ± 0.0006	0.0061 ± 0.0008	n.d.

Table S11. Normalized average product signals of unnatural substrates for ring expansion with Anc1 and Anc1 variants. For wells where no product signal was detected, the notation "n.d." has been used.

Substrate	Anc1	Anc1Rd1	Anc1Rd2	Anc1Rd3	Empty Vector
**2**	0.042 ± 0.001	0.052 ± 0.006	0.061 ± 0.002	0.057 ± 0.002	n.d.
**3**	0.090 ± 0.008	0.08 ± 0.01	0.077 ± 0.005	0.081 ± 0.004	n.d.
**4**	0.21 ± 0.01	0.199 ± 0.008	0.19 ± 0.03	0.18 ± 0.02	n.d.
**5**	0.10 ± 0.01	0.123 ± 0.006	0.054 ± 0.006	0.0631 ± 0.0009	n.d.
**S6**	0.071 ± 0.003	0.0901 ± 0.0007	0.113 ± 0.006	0.107 ± 0.007	n.d.

Figure S20. Extracted ion chromatograms from RF-MS of triplicate reactions (R1–3) with XenC and Anc1 variants on substrate **2.** UV-Vis spectra for tropolone product depicted at the bottom. Product mass peak area is reported over each peak and the chromatograms have been scaled to the highest peak area.

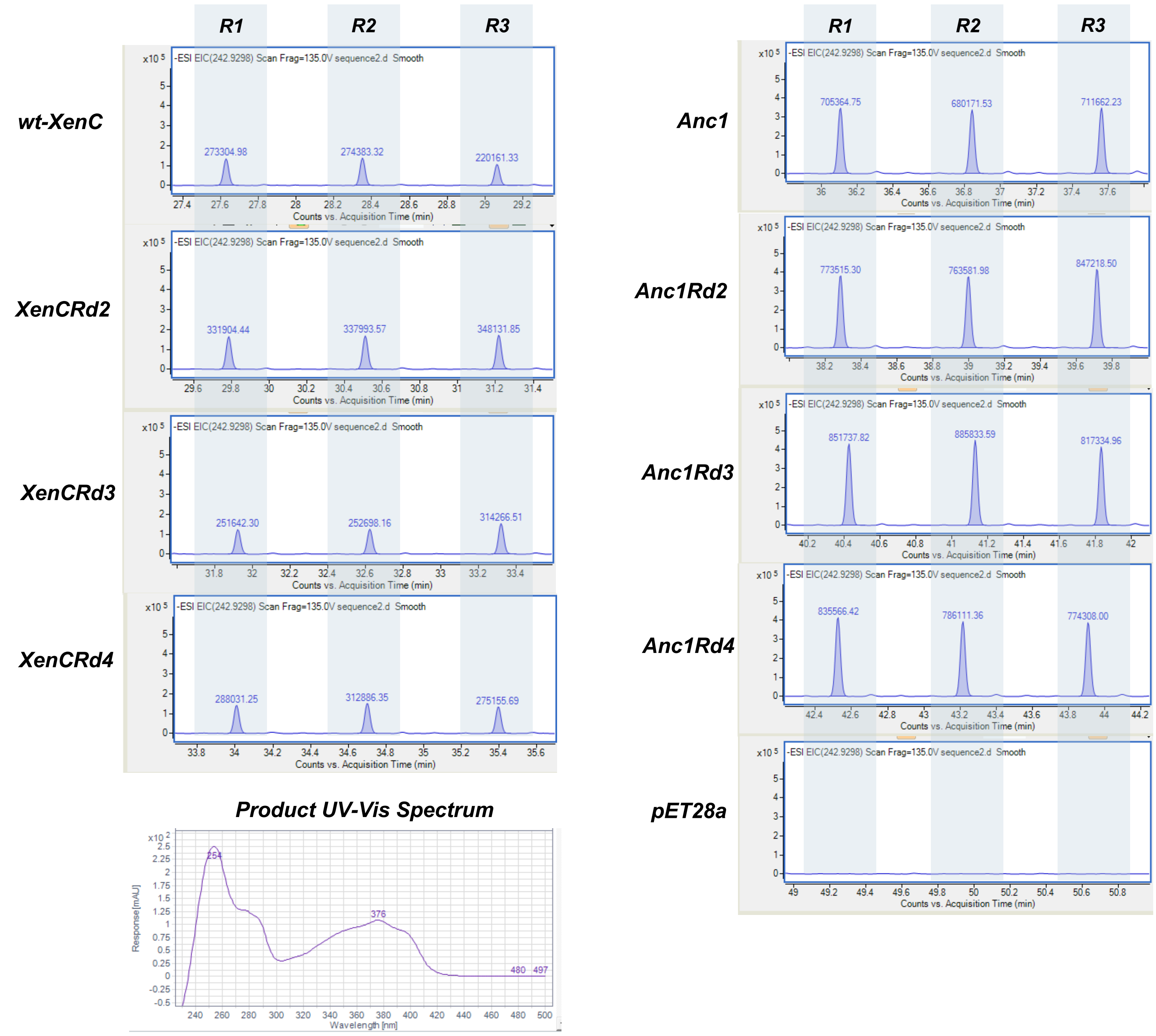

Figure S21. Extracted ion chromatograms from RF-MS of triplicate reactions (R1–3) with XenC and Anc1 variants on substrate **3.** UV-Vis spectra for tropolone product depicted at the bottom. Product mass peak area is reported over each peak and the chromatograms have been scaled to the highest peak area.

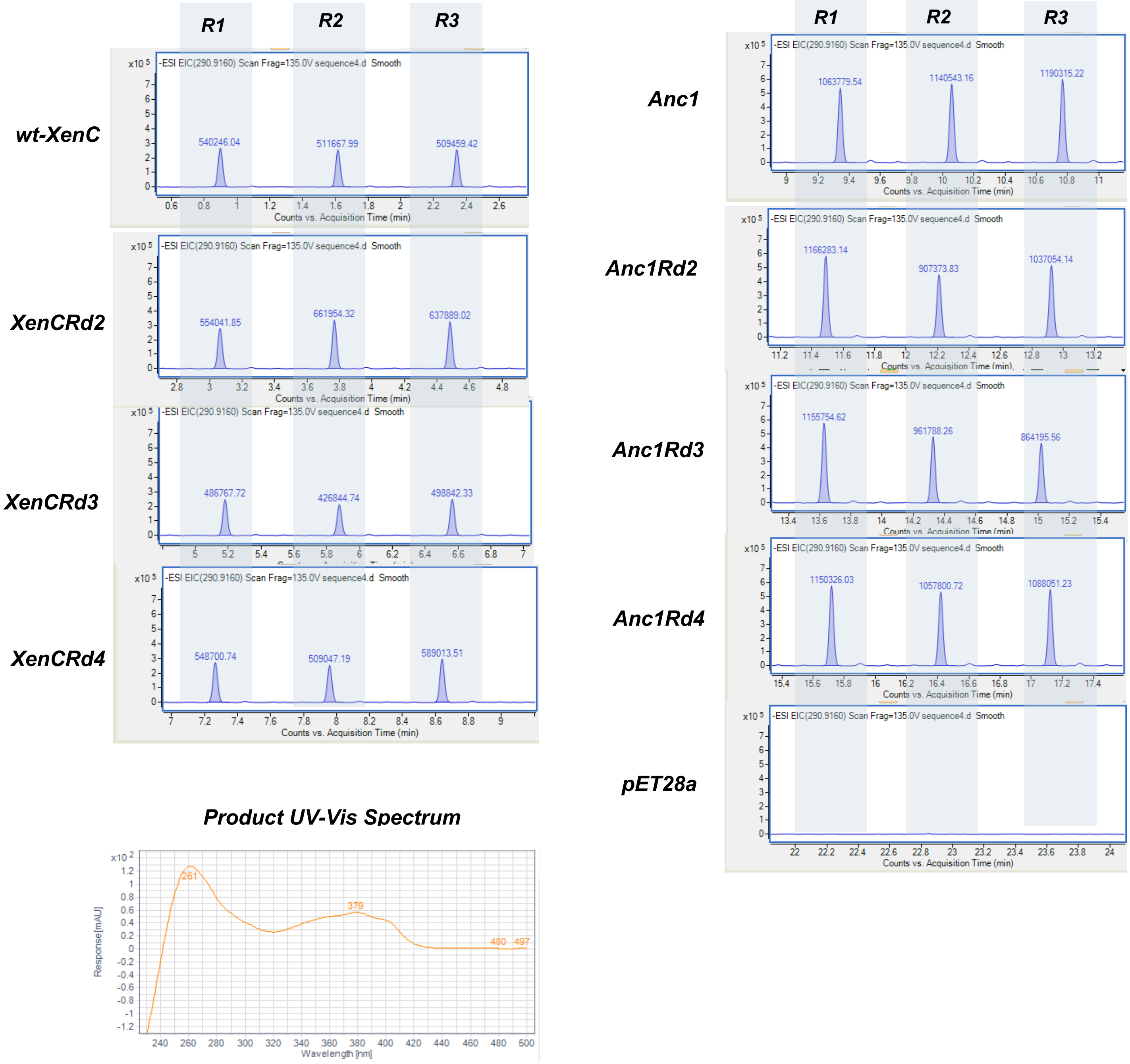

Figure S22. Extracted ion chromatograms from RF-MS of triplicate reactions (R1–3) with XenC and Anc1 variants on substrate **4.** UV-Vis spectra for tropolone product depicted at the bottom. Product mass peak area is reported over each peak and the chromatograms have been scaled to the highest peak area.

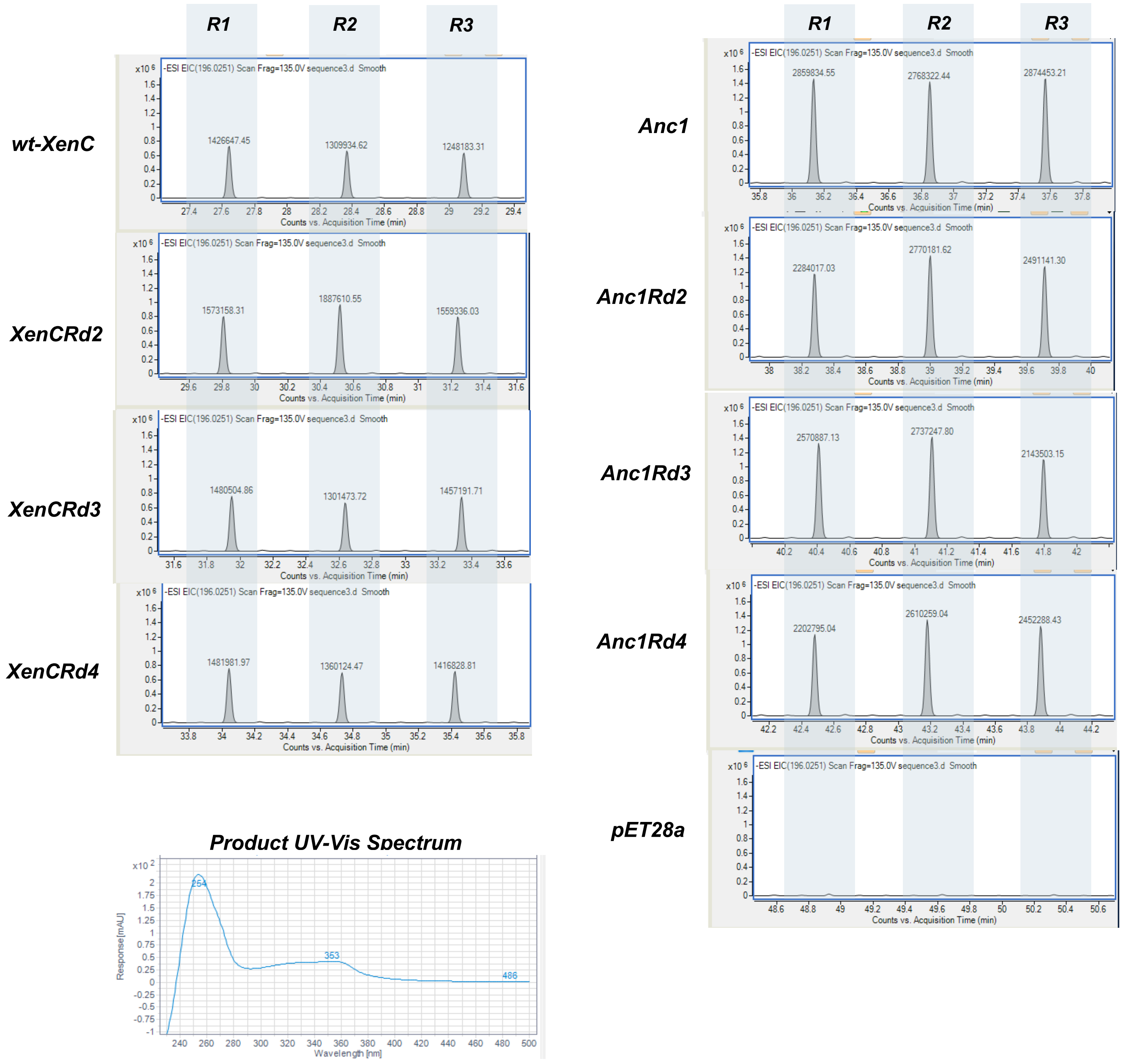

Figure S23. Extracted ion chromatograms from RF-MS of triplicate reactions (R1–3) with XenC and Anc1 variants on substrate **5.** UV-Vis spectra for tropolone product depicted at the bottom. Product mass peak area is reported over each peak and the chromatograms have been scaled to the highest peak area.

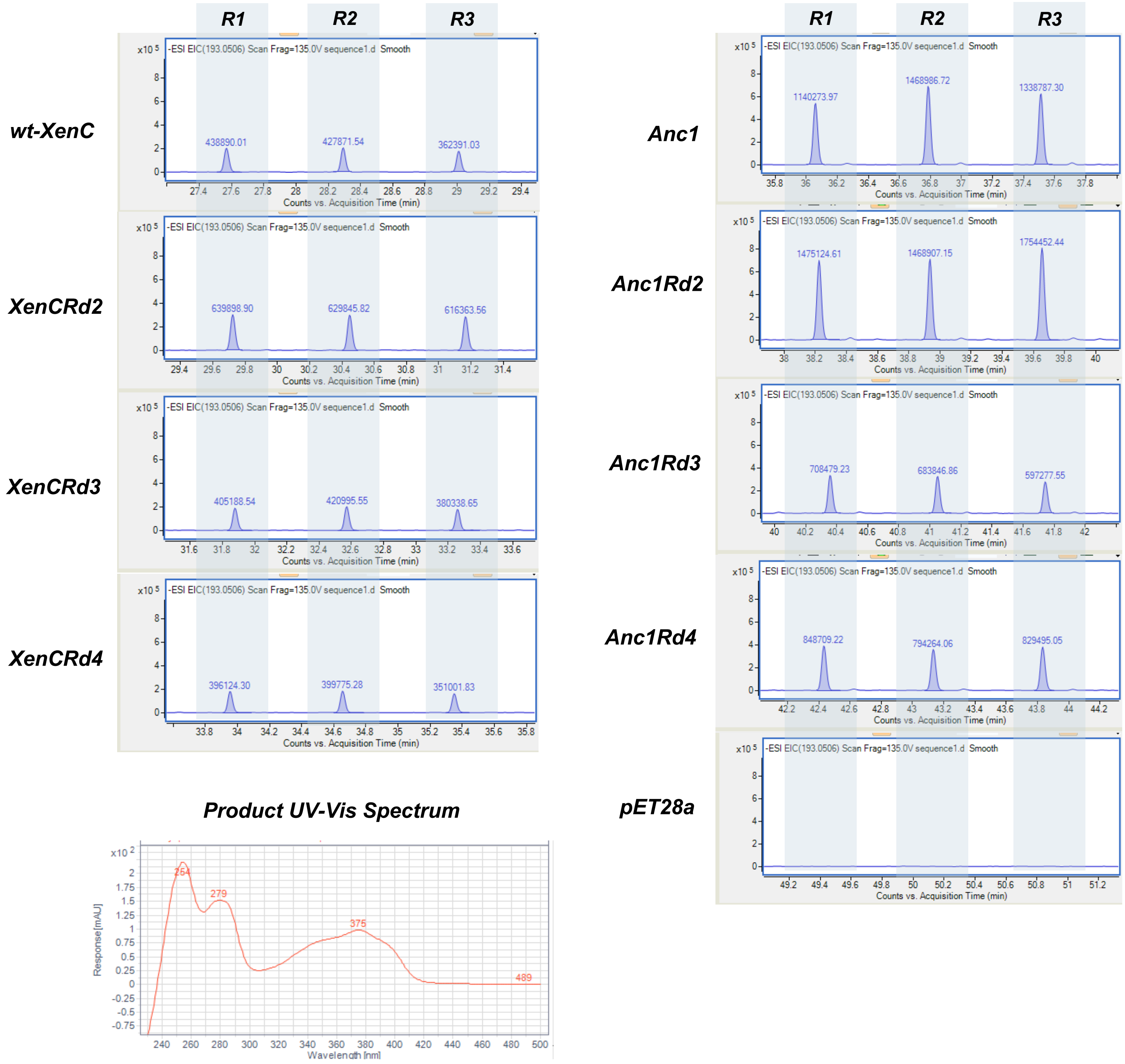

Figure S24. Extracted ion chromatograms from RF-MS of triplicate reactions (R1–3) with XenC and Anc1 variants on substrate **S6.** UV-Vis spectra for tropolone product depicted at the bottom. Product mass peak area is reported over each peak and the chromatograms have been scaled to the highest peak area.

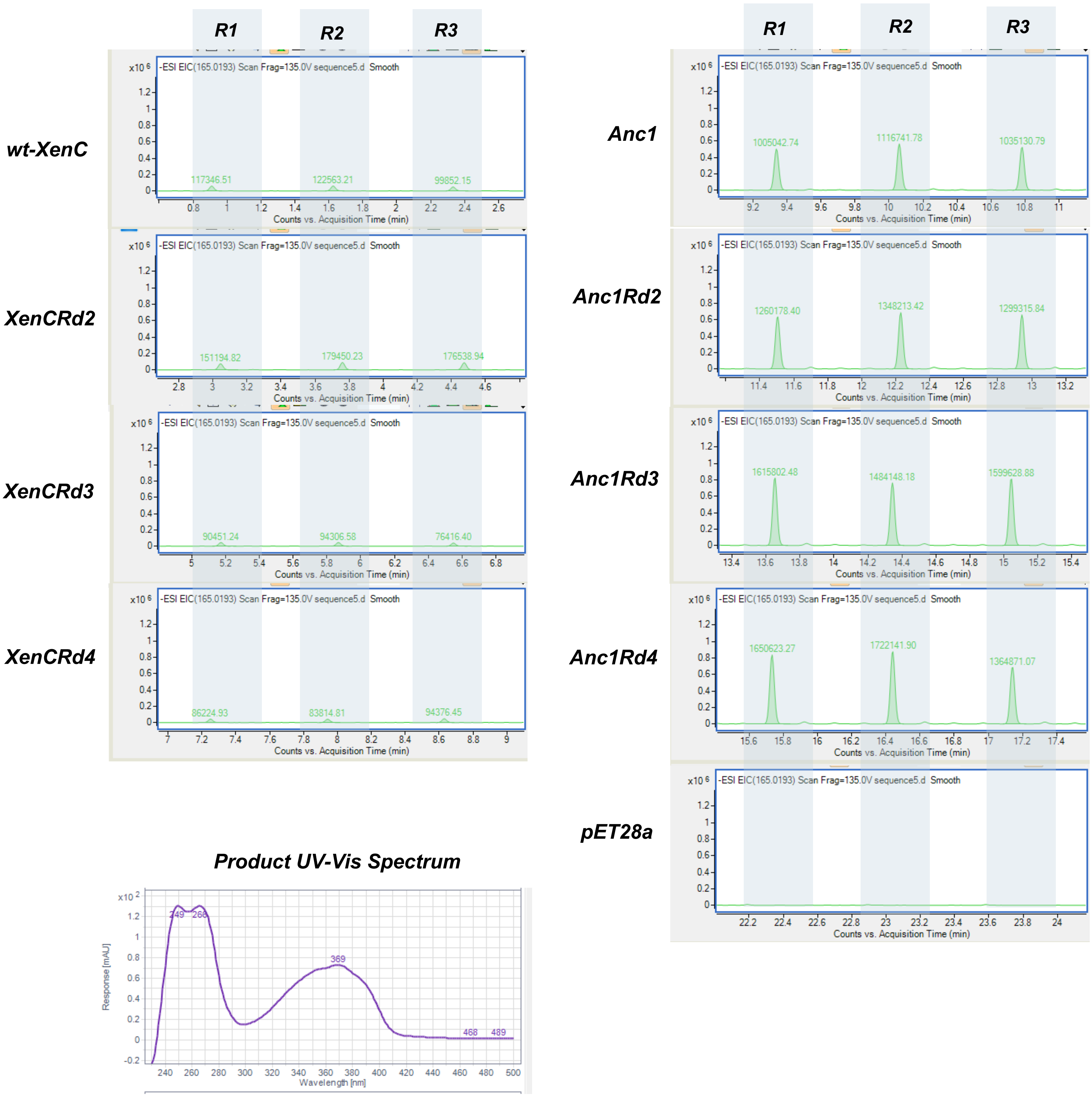

Determination of T_{50}. 96-well plates containing freshly harvested cells were resuspended in 250 µL of plate lysis buffer (50 mM TES pH 7.5, 2 mg/mL lysozyme, 0.5 mg/mL polymyxin B sulfate, 100 µM PMSF, and 1 U/mL DNaseI). The cells were lysed by incubation at 25 ºC, 350 rpm for 2 h and then centrifuged at 1,000 x g for 30 min at 4 ºC. The clarified lysate (50 µL) was heat treated in duplicates at a range of 30–55 ºC in 5 ºC intervals for 15 min using a thermocycler before transferring 20 µL of heat-treated lysate to a new plate with each well containing 80 µL reaction mix to reach a final concentration of 1.2 g/L dearomatized substrate from TropB reaction and 14 mM α-KG in 50 mM TES pH 7.5. Reactions were incubated at 30 ºC for 1 h and then quenched with 100 µL of 1 M HCl. After 10 min, 900 µL methanol containing 100 µM 2,6-dihydroxyacetophenone (internal standard) was added and plates were centrifuged at 1,000 x g for 30 min at 4 ºC to prepare analysis plates. Reactions were diluted as previously described for analysis using the optimized RF-MS method used in the library screening. The average of the highest activity was used as 100% residual activity to normalize the rest of the heat-treated samples. The T_{50} was determined as the approximate temperature were 50% residual activity remained after the incubation.

Figure S25. Residual activity curves after heat treatment of all the variants generated from the XenC and Anc1 evolutions to determine approximate T_{50} values.

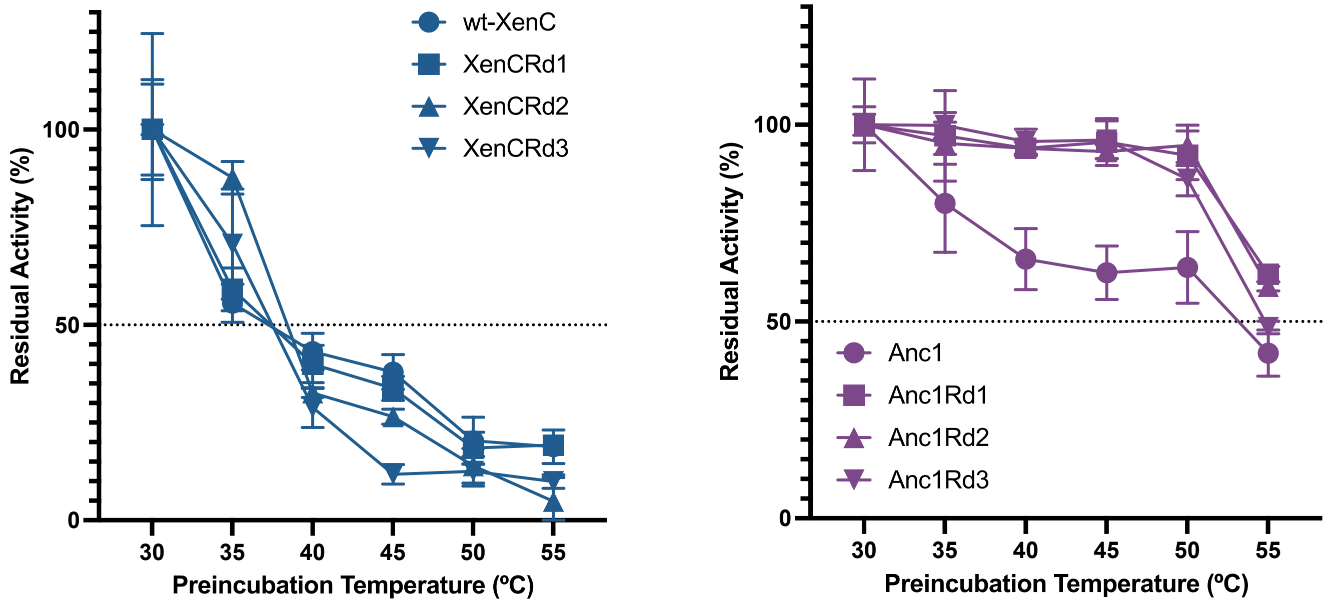

Table S12. Estimated T_{50} values for the enzymes investigated in this study based on T_{50} curves.

Enzyme	T_{50} (ºC)
**wt-XenC**	35 ± 2
**XenCRd1**	37.5 ± 0.7
**XenCRd2**	38.5 ± 0.7
**XenCRd3**	38.5 ± 1.1
**Anc1**	53.5 ± 0.7
**Anc1Rd1**	>55
**Anc1Rd2**	>55
**Anc1Rd3**	55.0 ± 0.7

Preparative-scale ring expansion reactions. Clarified lysate from freshly harvested *E. coli* pellet harboring the NHI of interest was heated at 50 ºC for 15 min on a heat block before centrifuging at 40,000 x g for 10 min at 4 ºC. The clarified NHI enzyme solution (18 mL) was added to 11.4 mL of 50 mM TES pH 7.5, 30 mL of 1.0 g/L dearomatized substrate from the crude TropB reaction, and 600 µL of 1 M α-KG to a final volume of 60 mL in a 1 L Erlenmeyer flask. The reaction was incubated at 37 ºC and 300 rpm shaking for 4 h before quenching with 10 mL of 6 M HCl. After an hour, 2 equivalent volumes of ethyl acetate were added to the flask and the product was extracted into the organic phase before trituration with cold water. The solid was filtered and dried under vacuum to afford pure stipitaldehyde (**6**) as a bright orange crystalline solid. **^{1}H NMR** (599 MHz, $CDCl_3$) δ (ppm) 10.06 (s, 1H), 6.92 (s, 1H), 6.89 ppm (s, 1H), 2.64 ppm (s, 3H). **^{13}C NMR** (151 MHz, $CDCl_3$) δ (ppm) 194.7, 176.0, 175.8, 162.8, 149.5, 124.1, 114.2, 109.7, 25.5. All spectra obtained were consistent with literature values.[1,4]

Figure S26. Representative SDS-PAGE gel of precipitate recovered after temperature treatment of XenC and Anc1Rd3 for preparative scale ring expansion reactions.

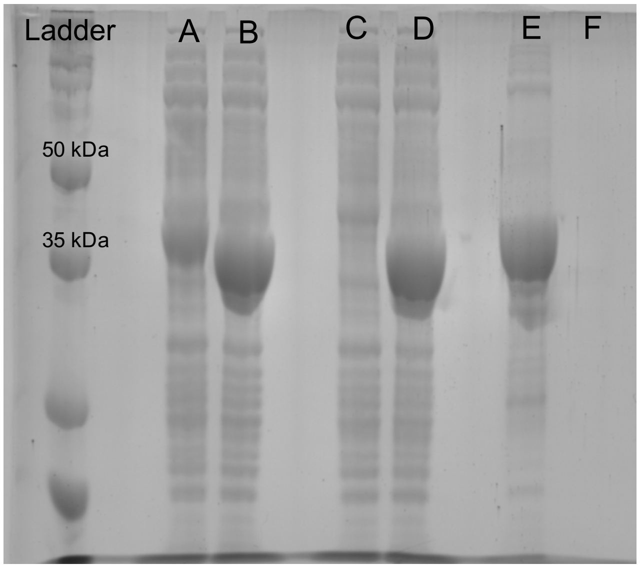

Lane	Sample
A	XenC clarified lysate
B	Anc1Rd3 clarified lysate
C	XenC heat treated lysate
D	Anc1Rd3 heat treated lysate
E	XenC heat treated precipitate
F	50 mM TES, pH 7.5 buffer

V. NMR Spectra

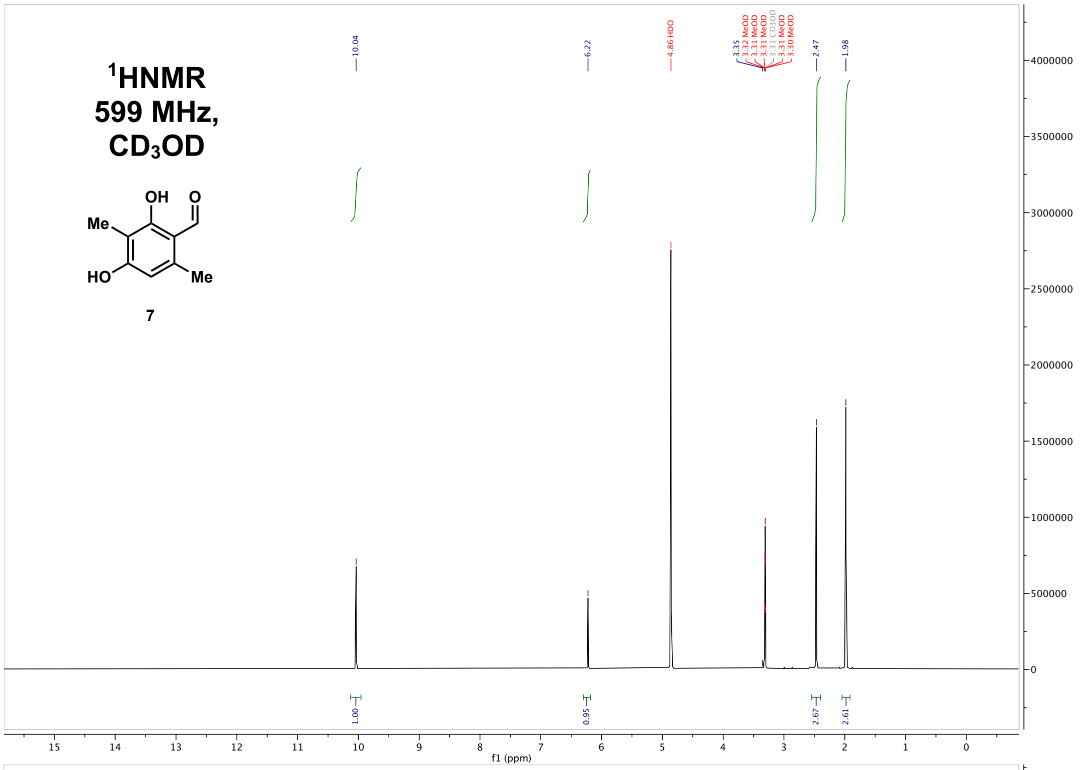

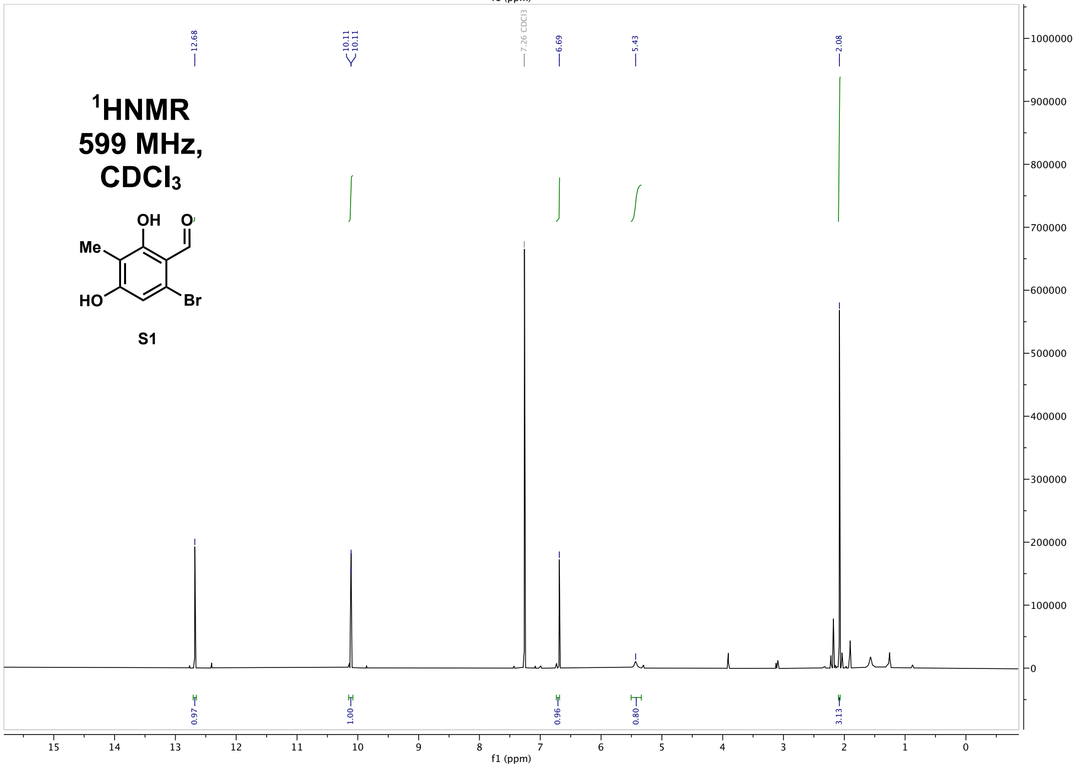

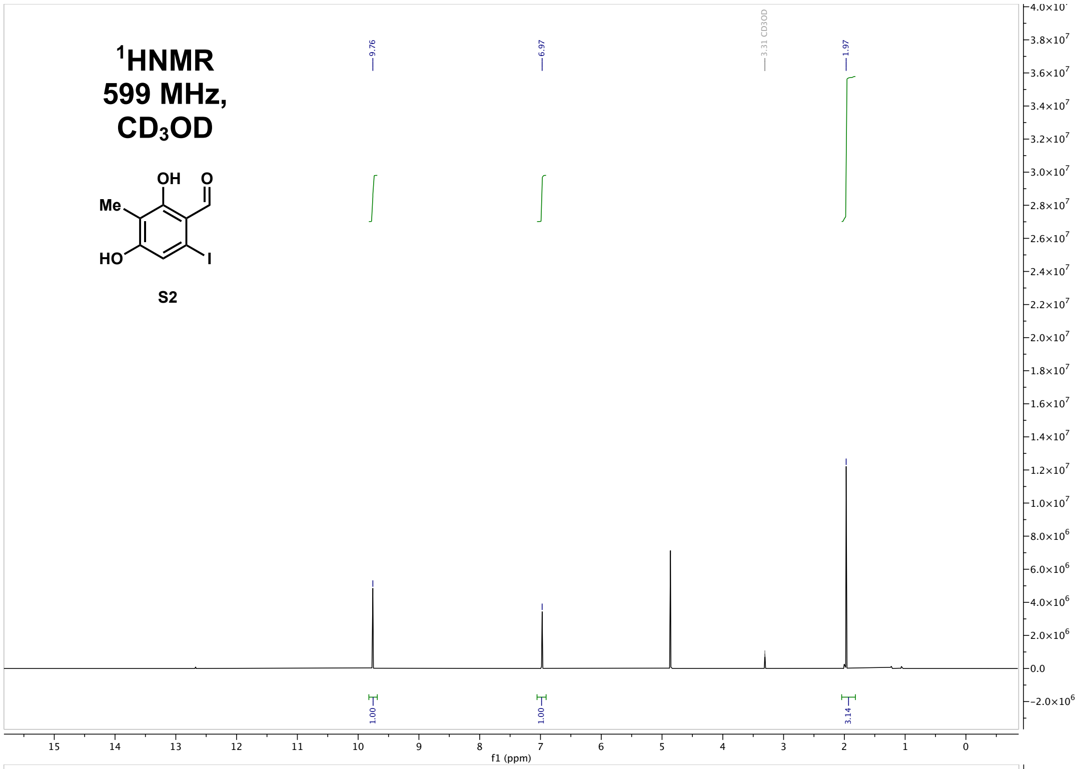
1HNMR
599 MHz,
CD3OD
OH
O
Me
HO
I
S2
9.76
6.97
3.31 CD3OD
1.97
1.00
1.00
3.14
f1 (ppm)

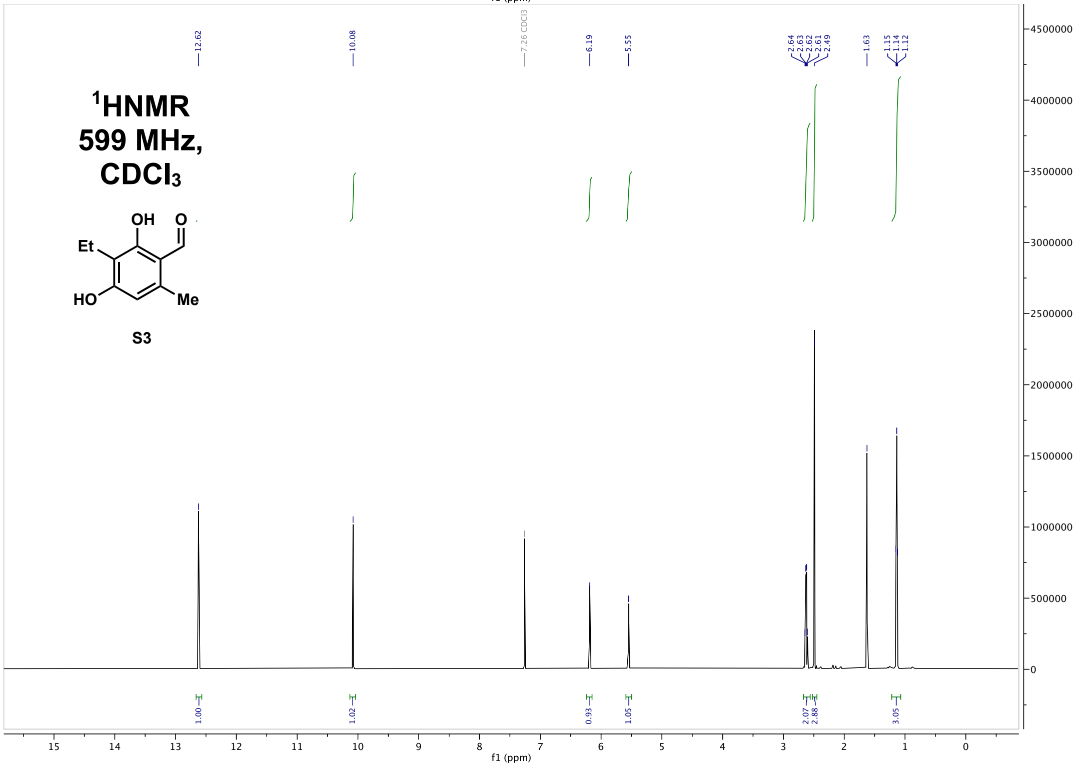
1HNMR
599 MHz,
CDCl3
OH
O
Et
HO
Me
S3
12.62
10.08
7.26 CDCl3
6.19
5.55
2.64
2.63
2.62
2.61
2.49
1.63
1.15
1.14
1.12
1.00
1.02
0.93
1.05
2.07
2.88
3.05
f1 (ppm)

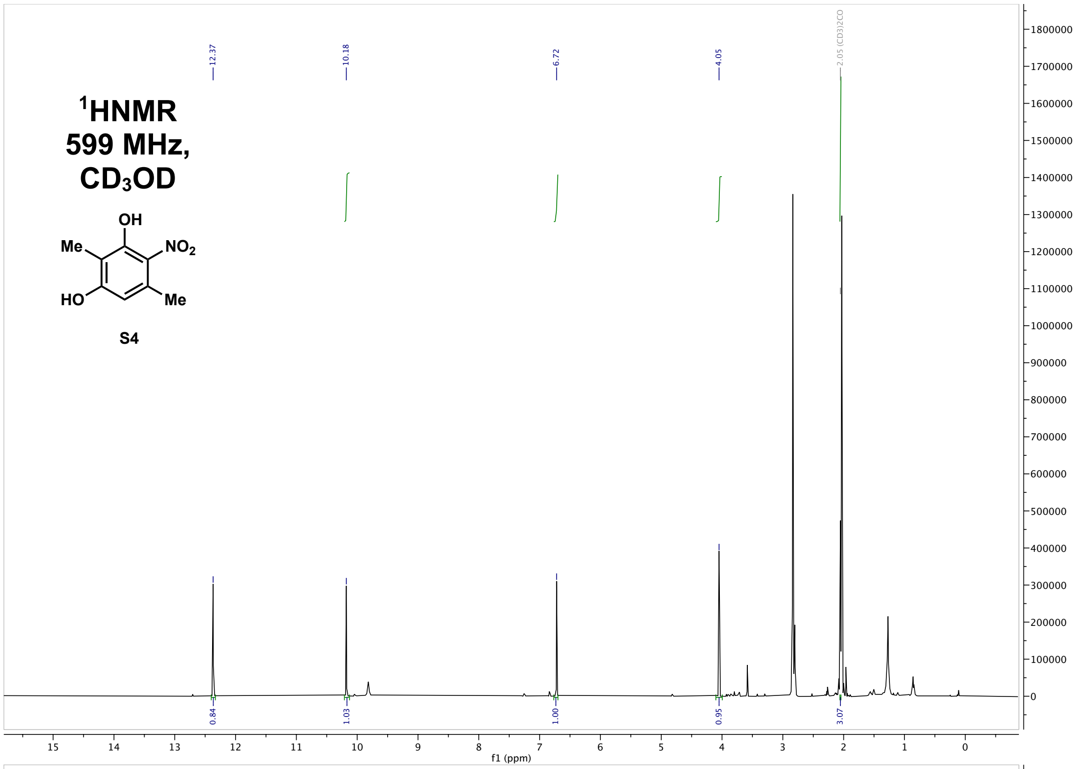

^{1}HNMR
599 MHz,
CD_3OD
OH
Me
NO2
HO
Me
S4
12.37
10.18
6.72
4.05
2.05 (CD3)2CO
0.84
1.03
1.00
0.95
3.07
f1 (ppm)

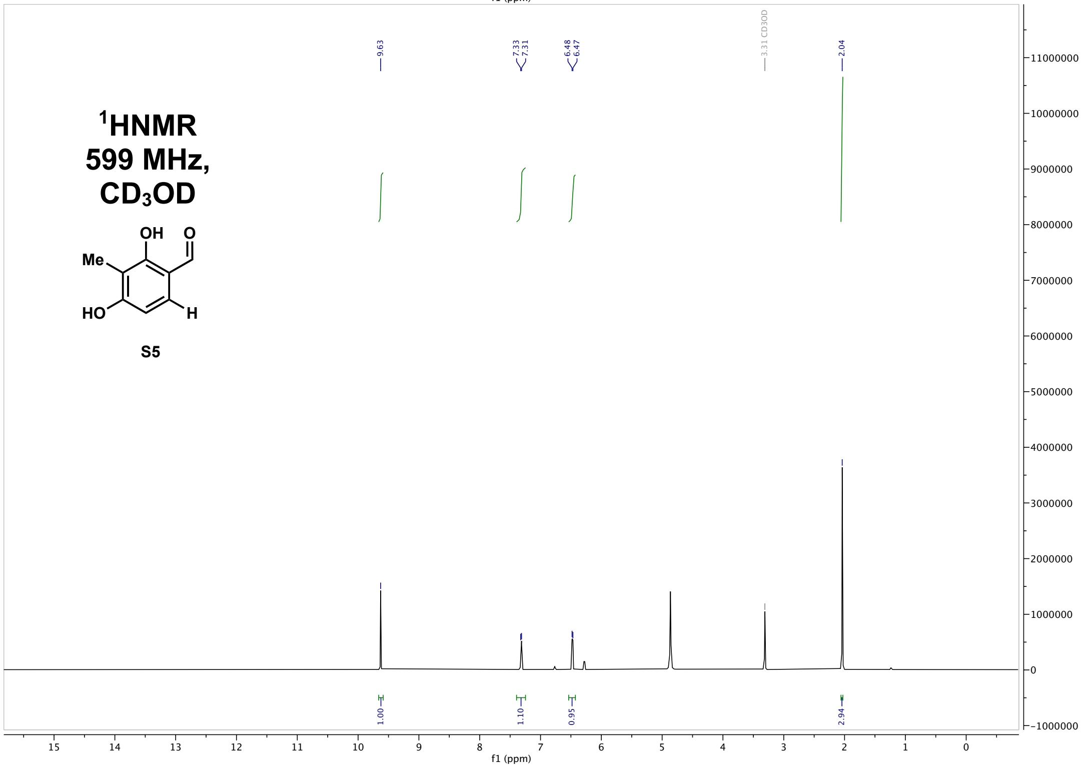

^{1}HNMR
599 MHz,
CD_3OD
OH
O
Me
HO
H
S5
9.63
7.33
7.31
6.48
6.47
3.31 CD3OD
2.04
1.00
1.10
0.95
2.94
f1 (ppm)

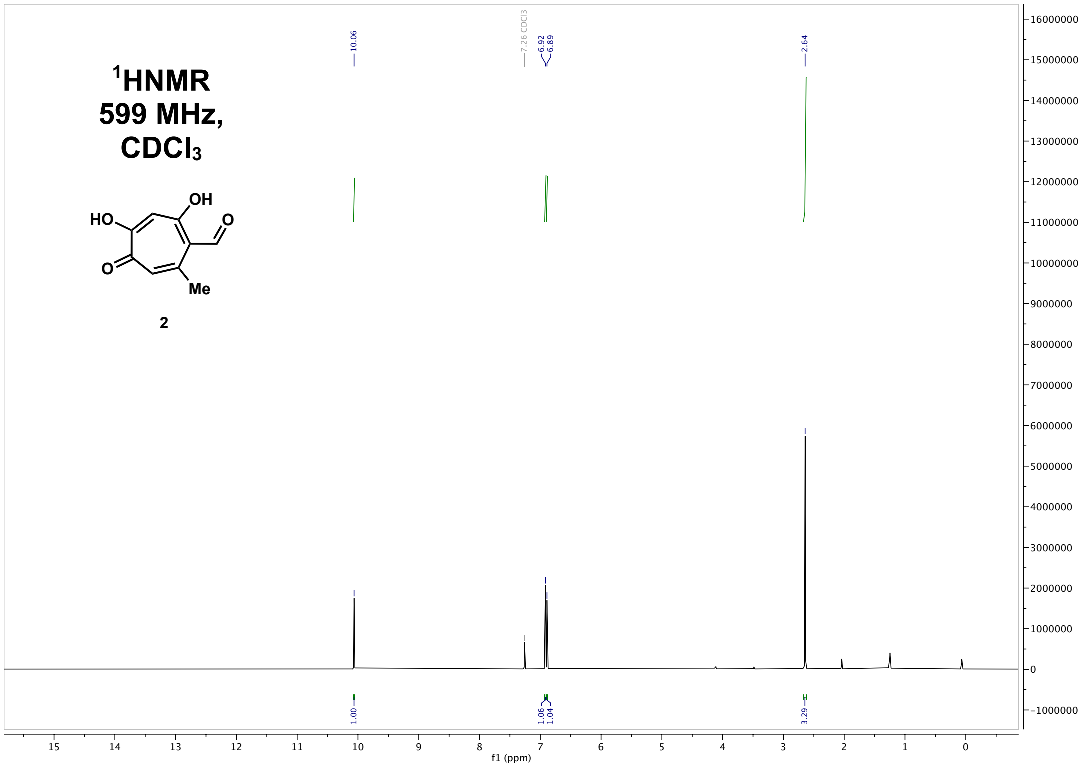

^{1}HNMR
599 MHz,
$CDCl_3$
HO
OH
O
O
Me
2
10.06
7.26 CDCl3
6.92
6.89
2.64
1.00
1.06
1.04
3.29
f1 (ppm)

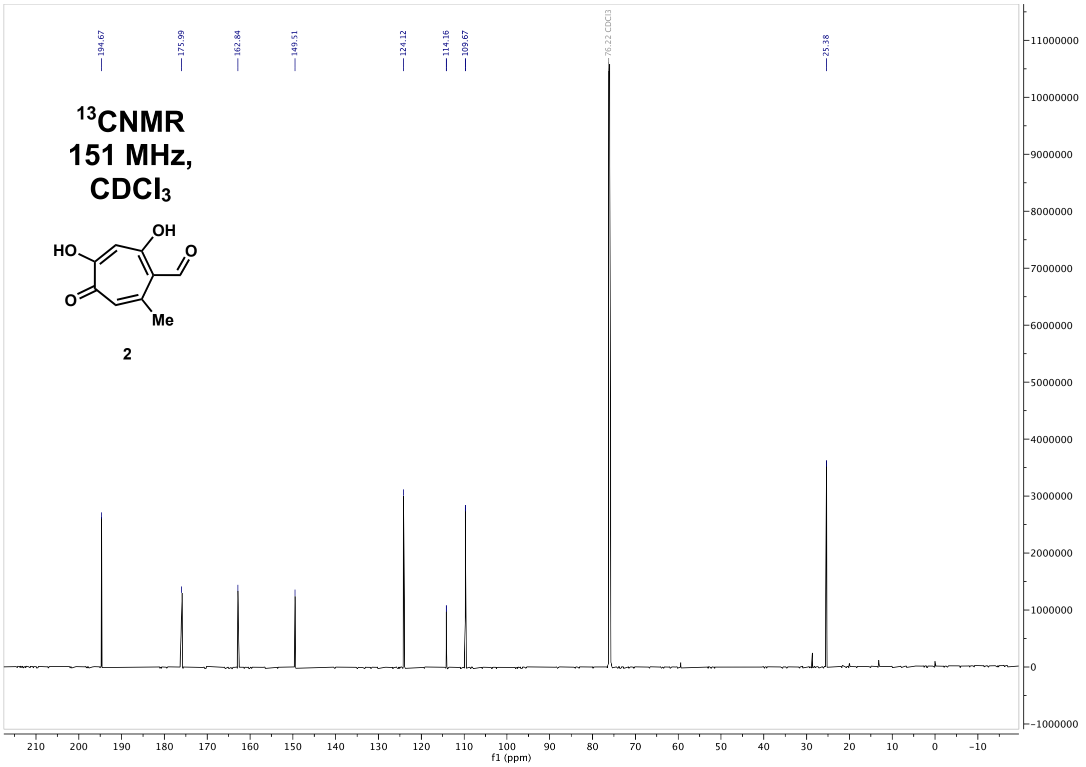

^{13}CNMR
151 MHz,
$CDCl_3$
HO
OH
O
O
Me
2
194.67
175.99
162.84
149.51
124.12
114.16
109.67
76.22 CDCl3
25.38
f1 (ppm)

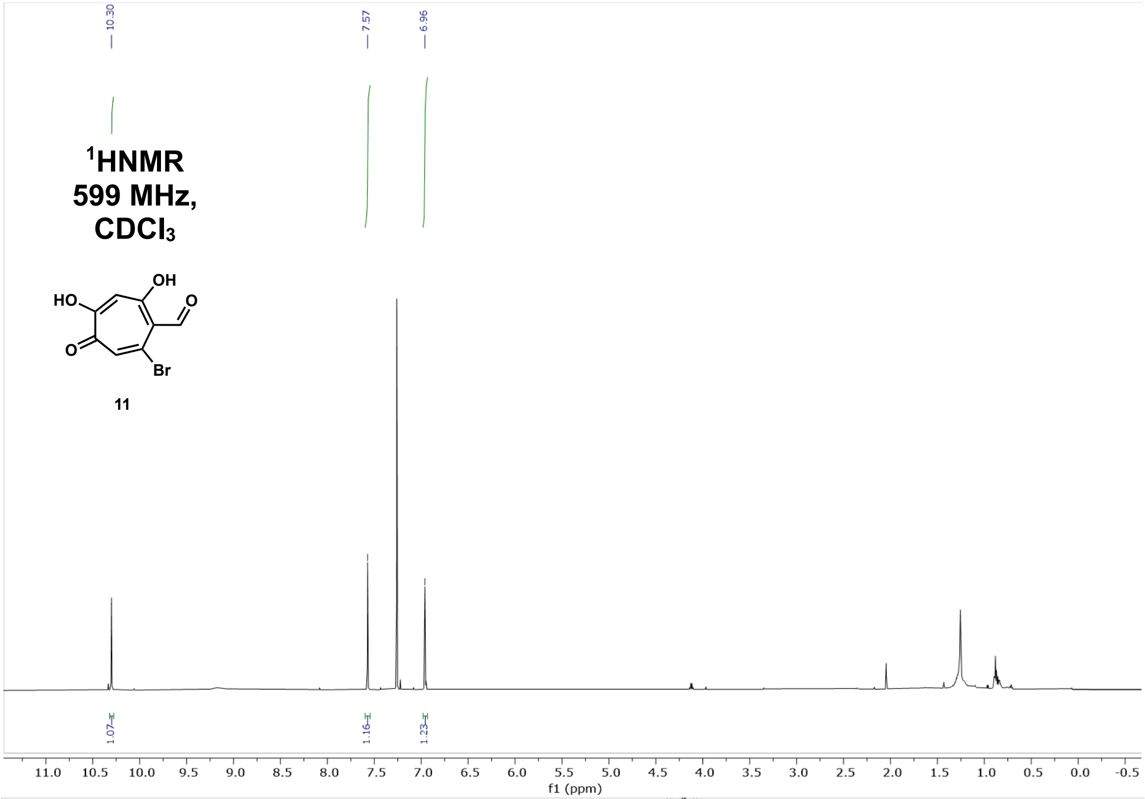

10.30
7.57
6.96
1HNMR
599 MHz,
CDCl3
OH
HO
O
O
Br
11
1.07
1.16
1.23
11.0 10.5 10.0 9.5 9.0 8.5 8.0 7.5 7.0 6.5 6.0 5.5 5.0 4.5 4.0 3.5 3.0 2.5 2.0 1.5 1.0 0.5 0.0 -0.5
f1 (ppm)

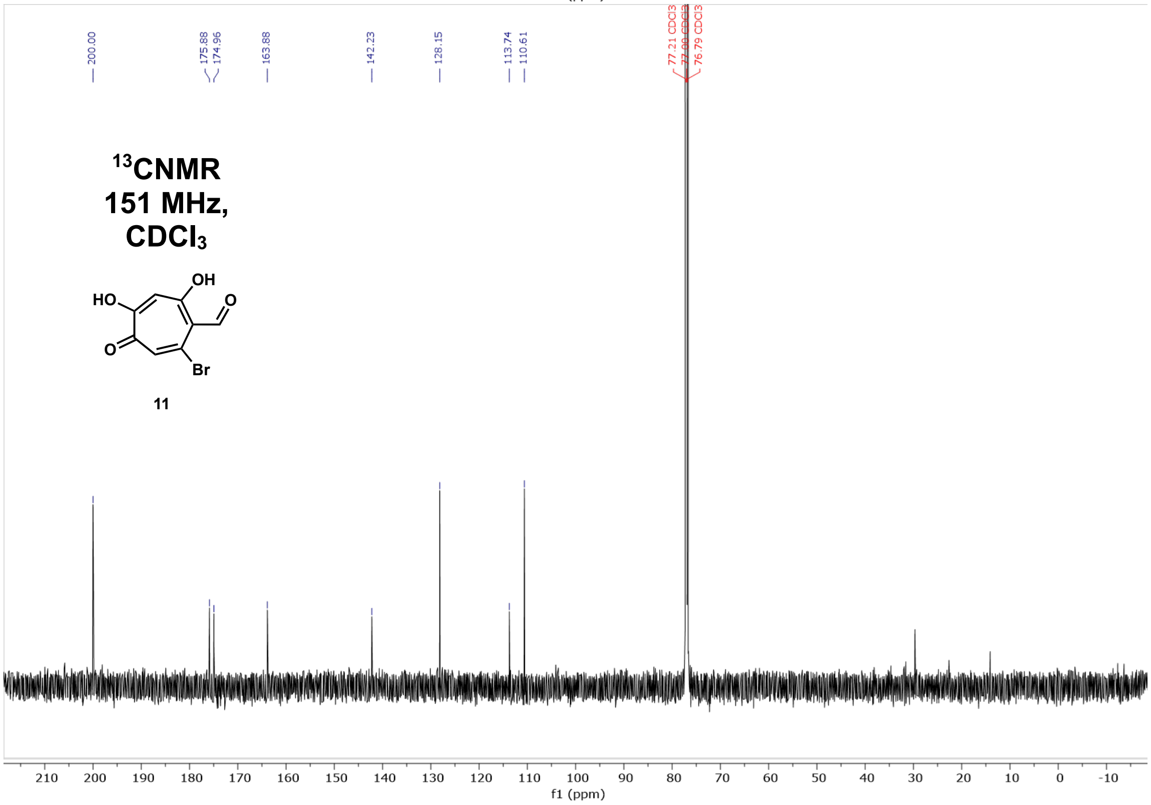

200.00
175.88
174.96
163.88
142.23
128.15
113.74
110.61
77.21 CDCl3
77.00 CDCl3
76.79 CDCl3
13CNMR
151 MHz,
CDCl3
OH
HO
O
O
Br
11
210 200 190 180 170 160 150 140 130 120 110 100 90 80 70 60 50 40 30 20 10 0 -10
f1 (ppm)

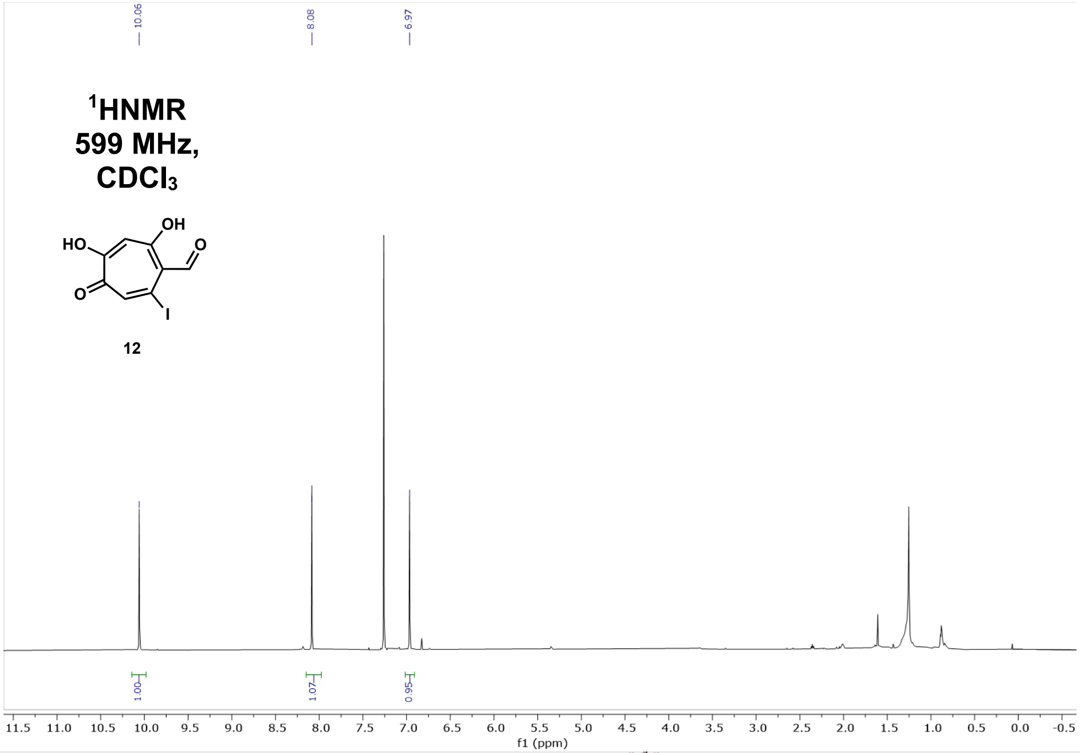

1HNMR
599 MHz,
CDCl3
HO
OH
O
O
I
12
10.06
8.08
6.97
1.00
1.07
0.95
11.5 11.0 10.5 10.0 9.5 9.0 8.5 8.0 7.5 7.0 6.5 6.0 5.5 5.0 4.5 4.0 3.5 3.0 2.5 2.0 1.5 1.0 0.5 0.0 -0.5
f1 (ppm)

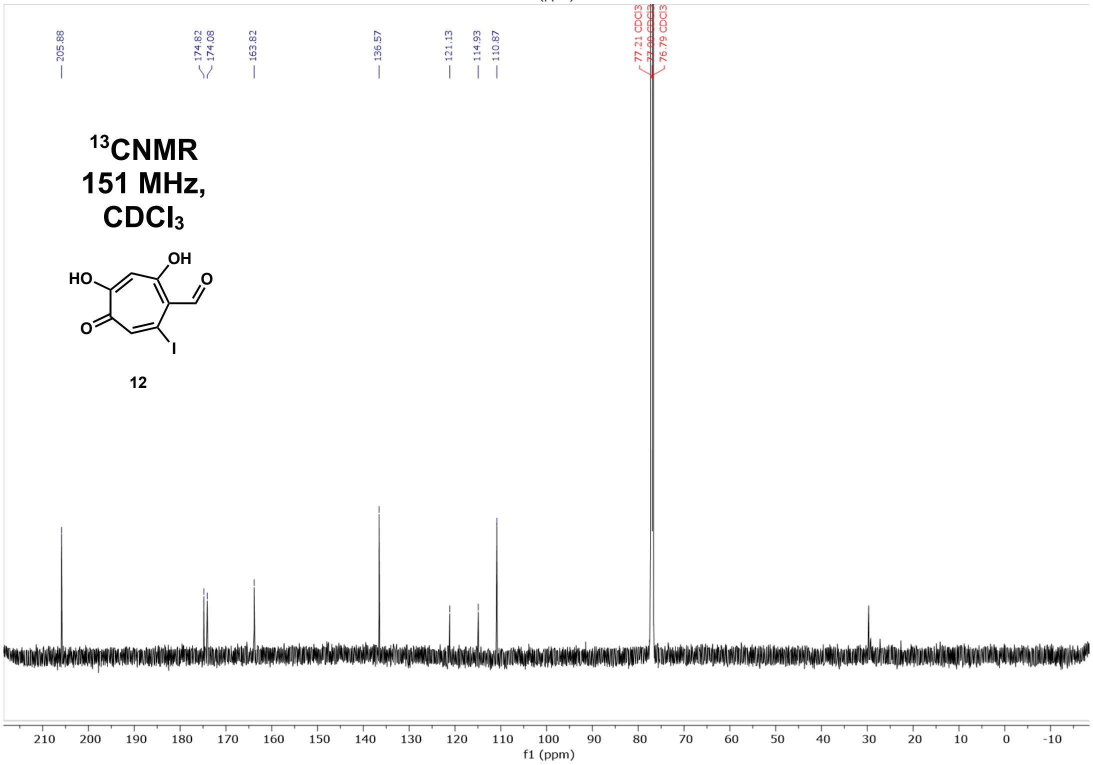

13CNMR
151 MHz,
CDCl3
HO
OH
O
O
I
12
205.88
174.82
174.08
163.82
136.57
121.13
114.93
110.87
77.21 CDCl3
76.79 CDCl3
210 200 190 180 170 160 150 140 130 120 110 100 90 80 70 60 50 40 30 20 10 0 -10
f1 (ppm)

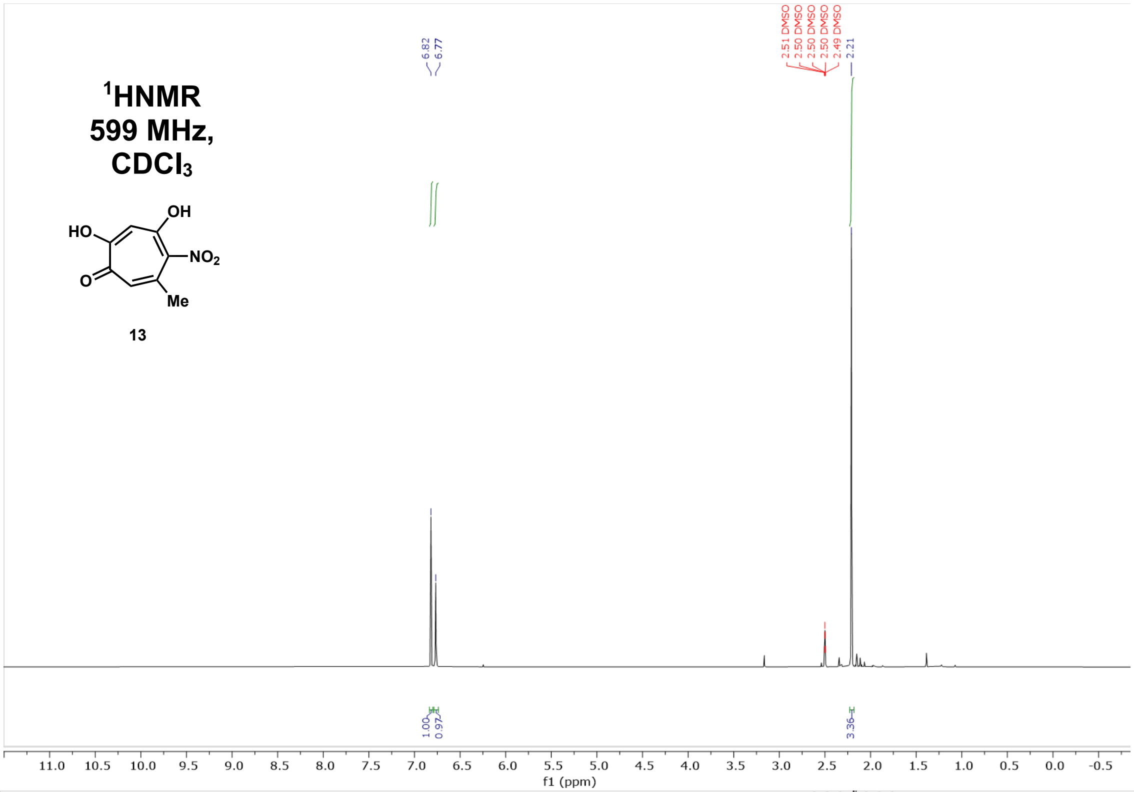

1HNMR
599 MHz,
CDCl3
HO
OH
NO2
O
Me
13
6.82
6.77
2.51 DMSO
2.50 DMSO
2.50 DMSO
2.50 DMSO
2.49 DMSO
2.21
1.00
0.97
3.36
11.0 10.5 10.0 9.5 9.0 8.5 8.0 7.5 7.0 6.5 6.0 5.5 5.0 4.5 4.0 3.5 3.0 2.5 2.0 1.5 1.0 0.5 0.0 -0.5
f1 (ppm)

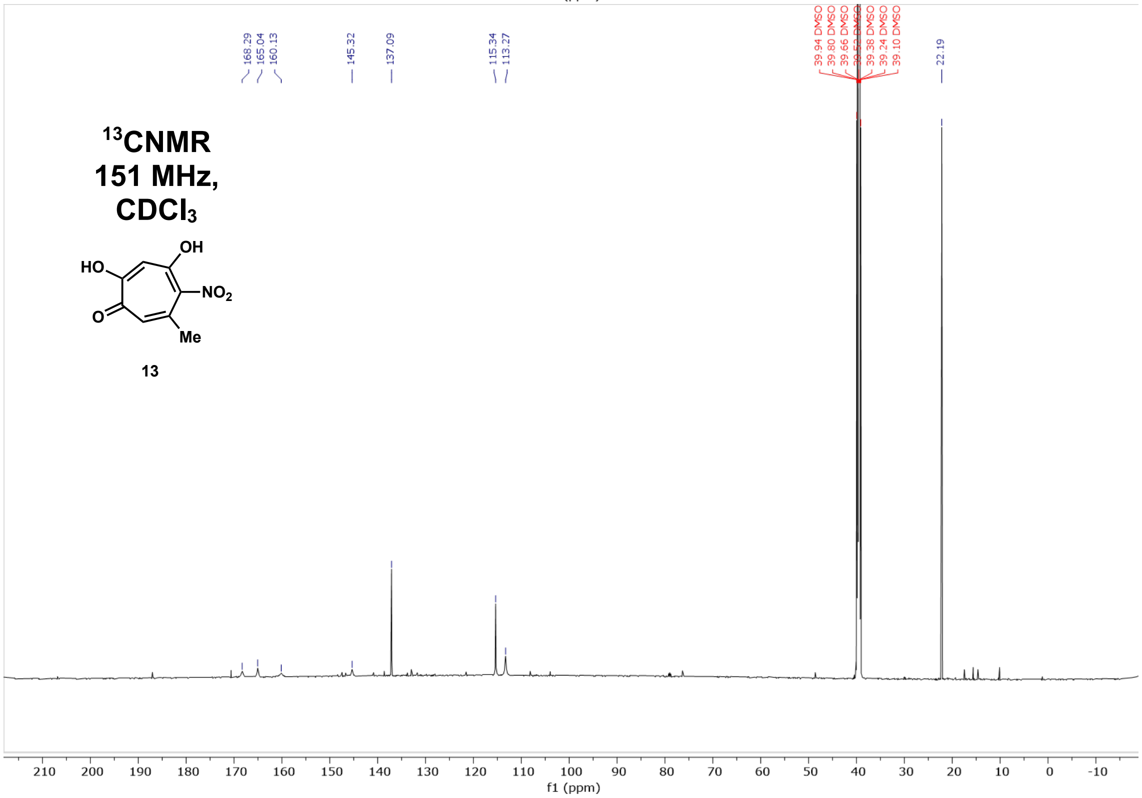

13CNMR
151 MHz,
CDCl3
HO
OH
NO2
O
Me
13
168.29
165.04
160.13
145.32
137.09
115.34
113.27
39.94 DMSO
39.80 DMSO
39.66 DMSO
39.52 DMSO
39.38 DMSO
39.24 DMSO
39.10 DMSO
22.19
210 200 190 180 170 160 150 140 130 120 110 100 90 80 70 60 50 40 30 20 10 0 -10
f1 (ppm)

VI. References

(1) Perkins, J. C.; Golliher, A. E.; Hernández-Meléndez, J. R.; Saucedo, A. T.; Doyon, T. J.; Narayan, A. R. H. Enzyme Library-Enabled Chemoenzymatic Tropolone Synthesis. Ver. 1. *ChemRxiv,* June 13, 2023. DOI: https://doi.org/10.26434/chemrxiv-2023-lvngd.

(2) Baker Dockrey, S. A.; Lukowski, A. L.; Becker, M. R.; Narayan, A. R. H. Biocatalytic Site- and Enantioselective Oxidative Dearomatization of Phenols. *Nat. Chem.* **2018**, *10* (2), 119–125. DOI: https://doi.org/10.1038/nchem.2879.

(3) Doyon, T. J.; Perkins, J. C.; Baker Dockrey, S. A.; Romero, E. O.; Skinner, K. C.; Zimmerman, P. M.; Narayan, A. R. H. Chemoenzymatic O-Quinone Methide Formation. *J. Am. Chem. Soc.* **2019**, *141* (51), 20269–20277. DOI: https://doi.org/10.1021/jacs.9b10474.

(4) Yang, D.; Chiang, C. H.; Wititsuwannakul, T.; Brooks, C. L.; Zimmerman, P. M.; Narayan, A. R. H. Engineering the Reaction Pathway of a Non-Heme Iron Oxygenase Using Ancestral Sequence Reconstruction. *J. Am. Chem. Soc.* **2024**, *146* (50), 34352–34363. DOI: https://doi.org/10.1021/jacs.4c08420.

(5) Mirdita, M.; Schütze, K.; Moriwaki, Y.; Heo, L.; Ovchinnikov, S.; Steinegger, M. ColabFold: Making Protein Folding Accessible to All. *Nat. Methods* **2022**, *19* (6), 679–682. DOI: https://doi.org/10.1038/s41592-022-01488-1.

(6) Doyon, T. J.; Skinner, K. C.; Yang, D.; Mallik, L.; Wymore, T.; Koutmos, M.; Zimmerman, P. M.; Narayan, A. R. H. Radical Tropolone Biosynthesis. Ver. 1. August 1, 2020. DOI: 10.26434/chemrxiv.12780044.v1.

(7) McLachlan, M. J.; Johannes, T. W.; Zhao, H. Further Improvement of Phosphite Dehydrogenase Thermostability by Saturation Mutagenesis. *Biotechnol. Bioeng.* **2008**, *99* (2), 268–274. DOI: https://doi.org/10.1002/bit.21546.
